# Supplementary figures and images for: Active site geometry stabilization of a presenilin homolog by the lipid bilayer promotes intramembrane proteolysis
Source: eLife. 2022 May 17;11:e76090. doi: 10.7554/eLife.76090 (PMC9282858; doi:10.7554/eLife.76090)

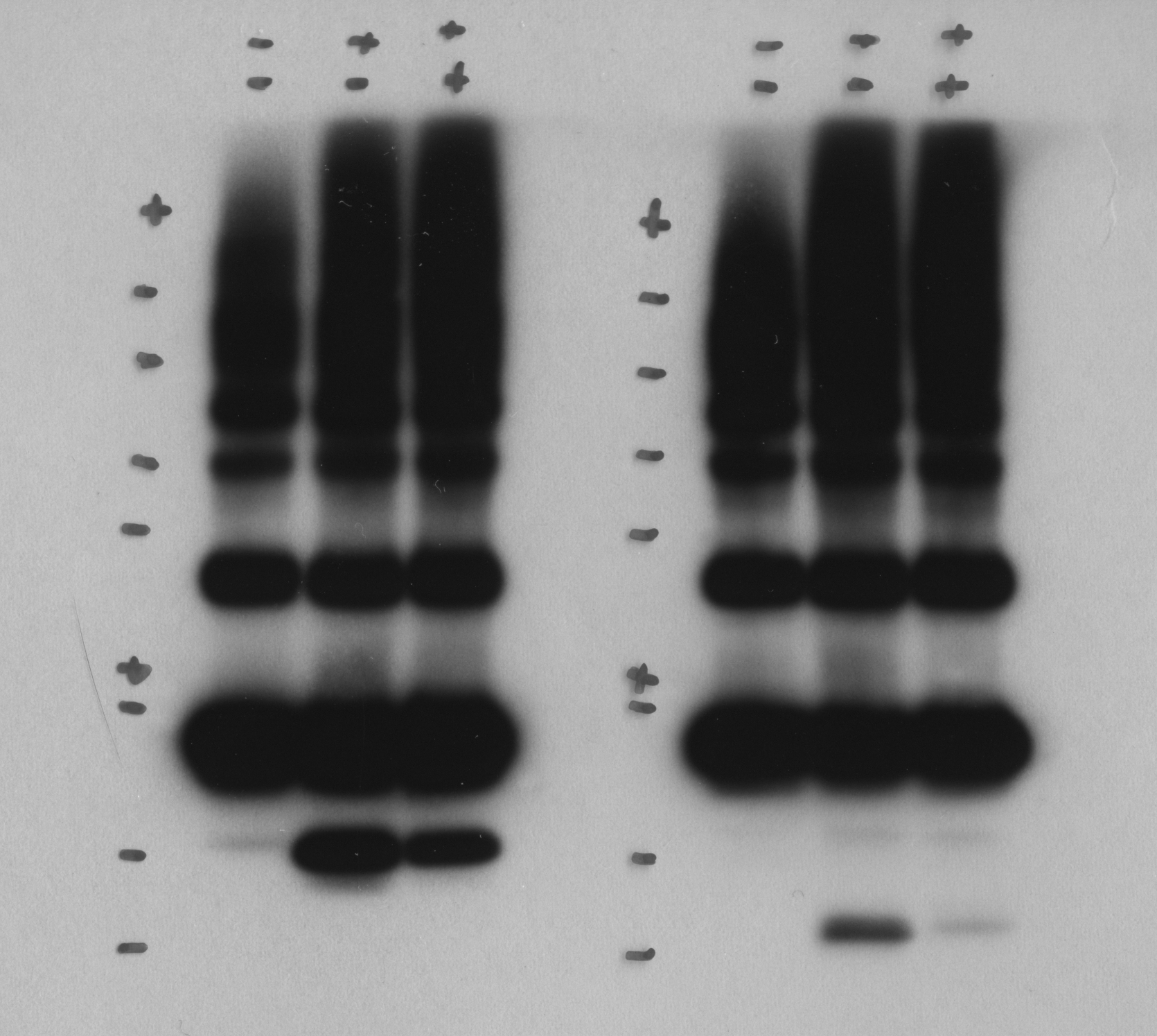

Supplement: Figure 1—source data 1. [file elife-76090-fig1-data1.zip › Figure1-source data1/Figure1B/Figure1B-Abeta 3.tif]

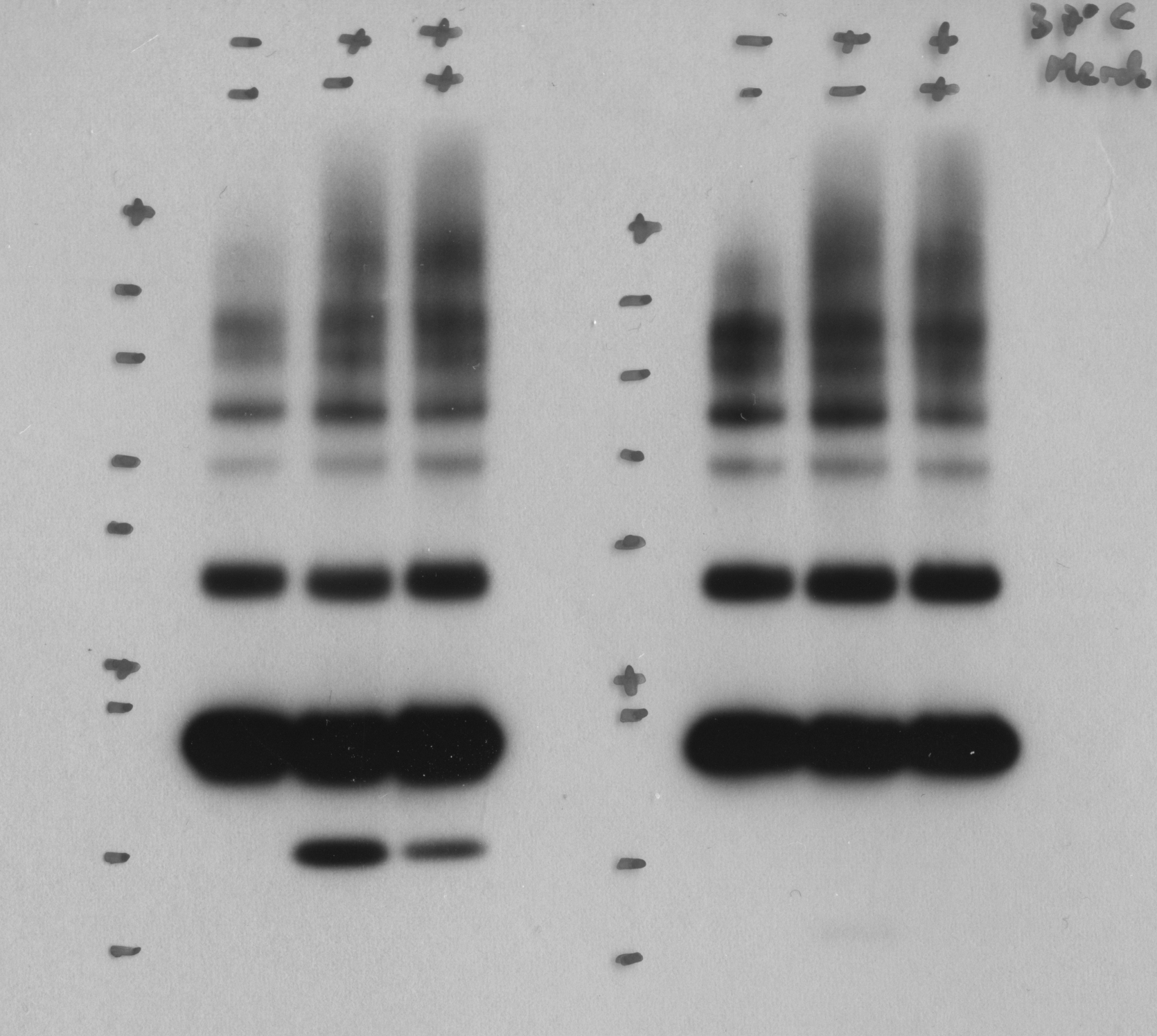

Supplement: Figure 1—source data 1. [file elife-76090-fig1-data1.zip › Figure1-source data1/Figure1B/Figure1B-AICD.tif]

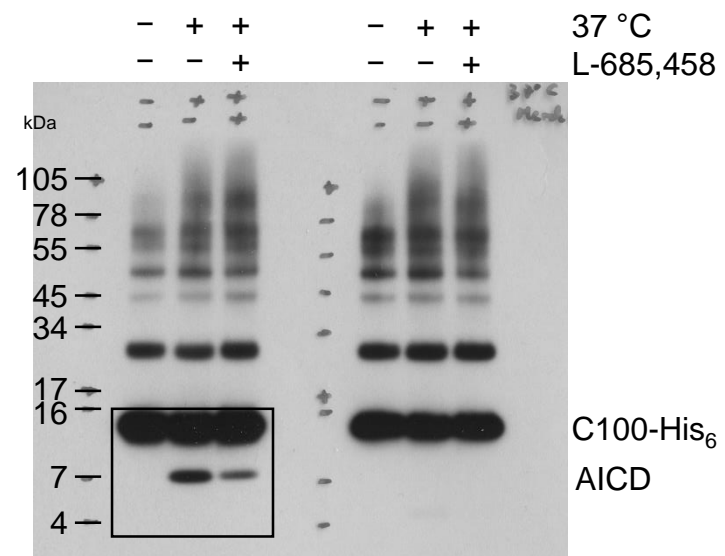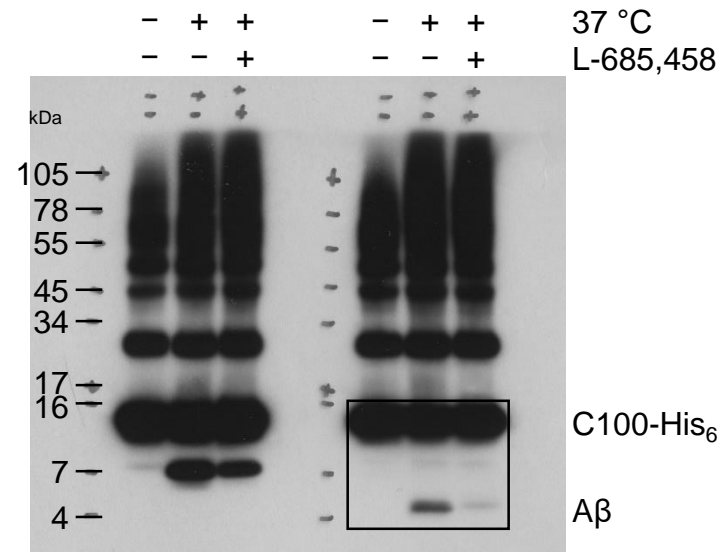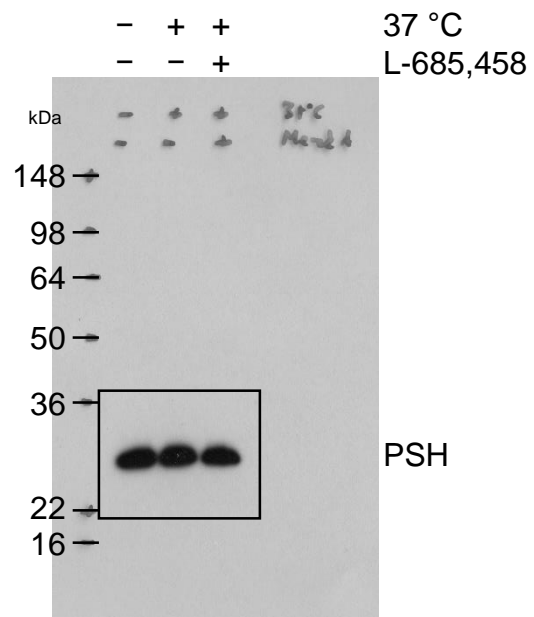

Supplement: Figure 1—source data 1. [file elife-76090-fig1-data1.zip › Figure1-source data1/Figure1B/Figure1B-annotated blots.pdf]

## Slide 1
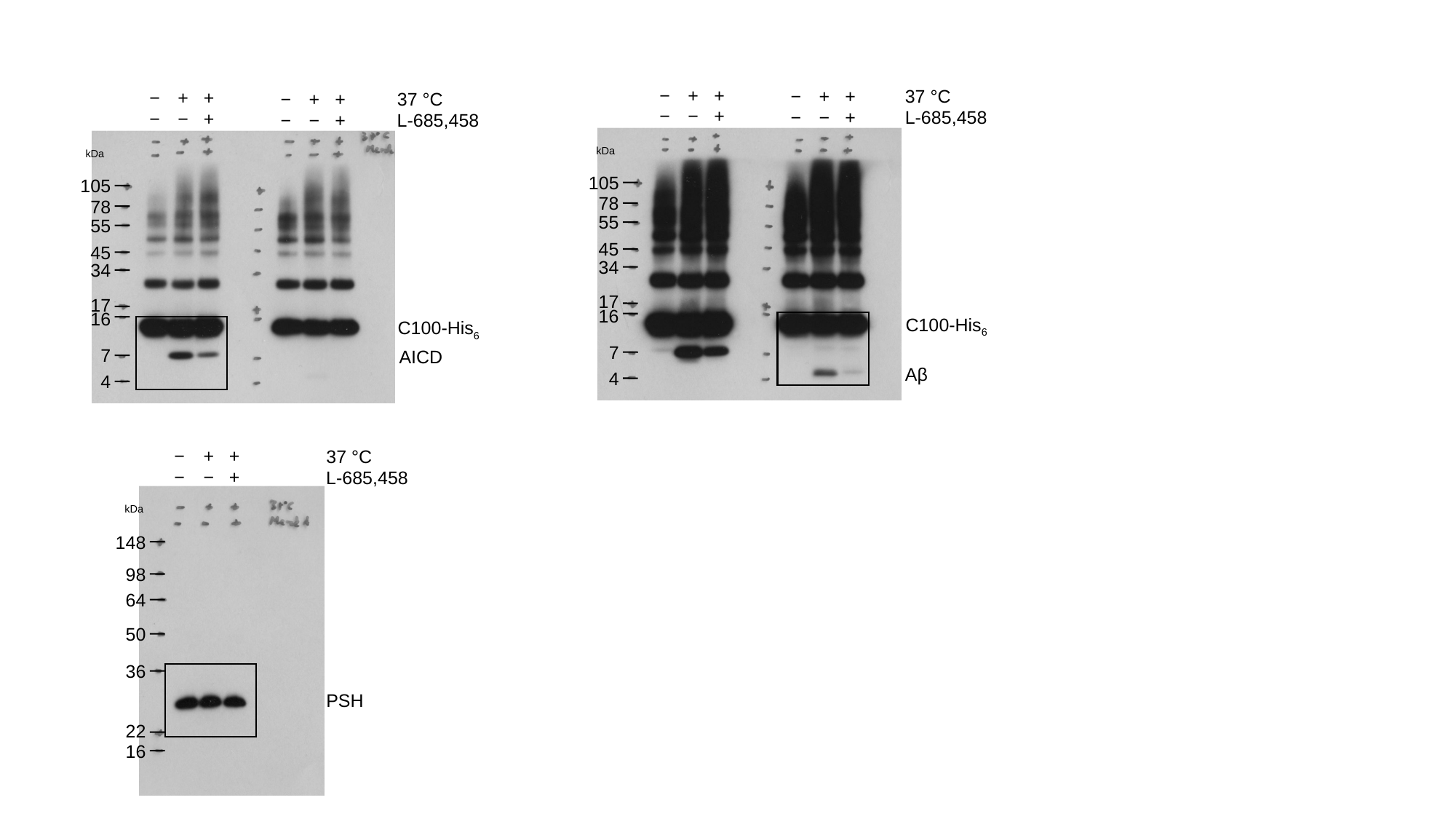

−
+
+
−
+
+
−
+
+
−
+
+
37 °C
37 °C
−
−
+
−
−
+
−
−
+
−
−
+
L-685,458
L-685,458
kDa
kDa
105
105
78
78
55
55
45
45
34
34
17
17
16
16
C100-His6
C100-His6
7
7
AICD
Aβ
4
4
−
+
+
37 °C
−
−
+
L-685,458
kDa
148
98
64
50
36
PSH
22
16

Supplement: Figure 1—source data 1. [file elife-76090-fig1-data1.zip › Figure1-source data1/Figure1B/Figure1B-annotated blots.pptx]

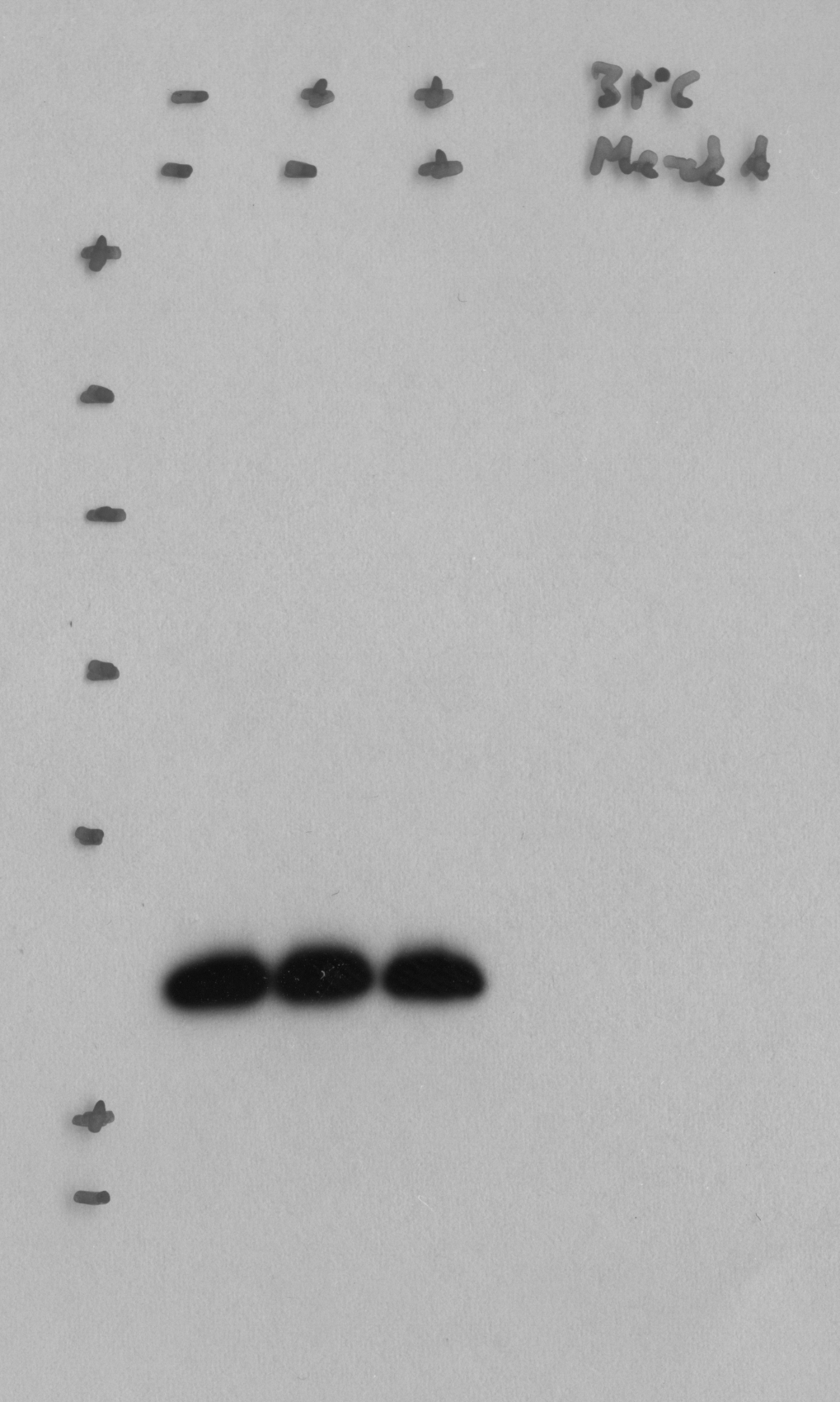

Supplement: Figure 1—source data 1. [file elife-76090-fig1-data1.zip › Figure1-source data1/Figure1B/Figure1B-PSH.tif]

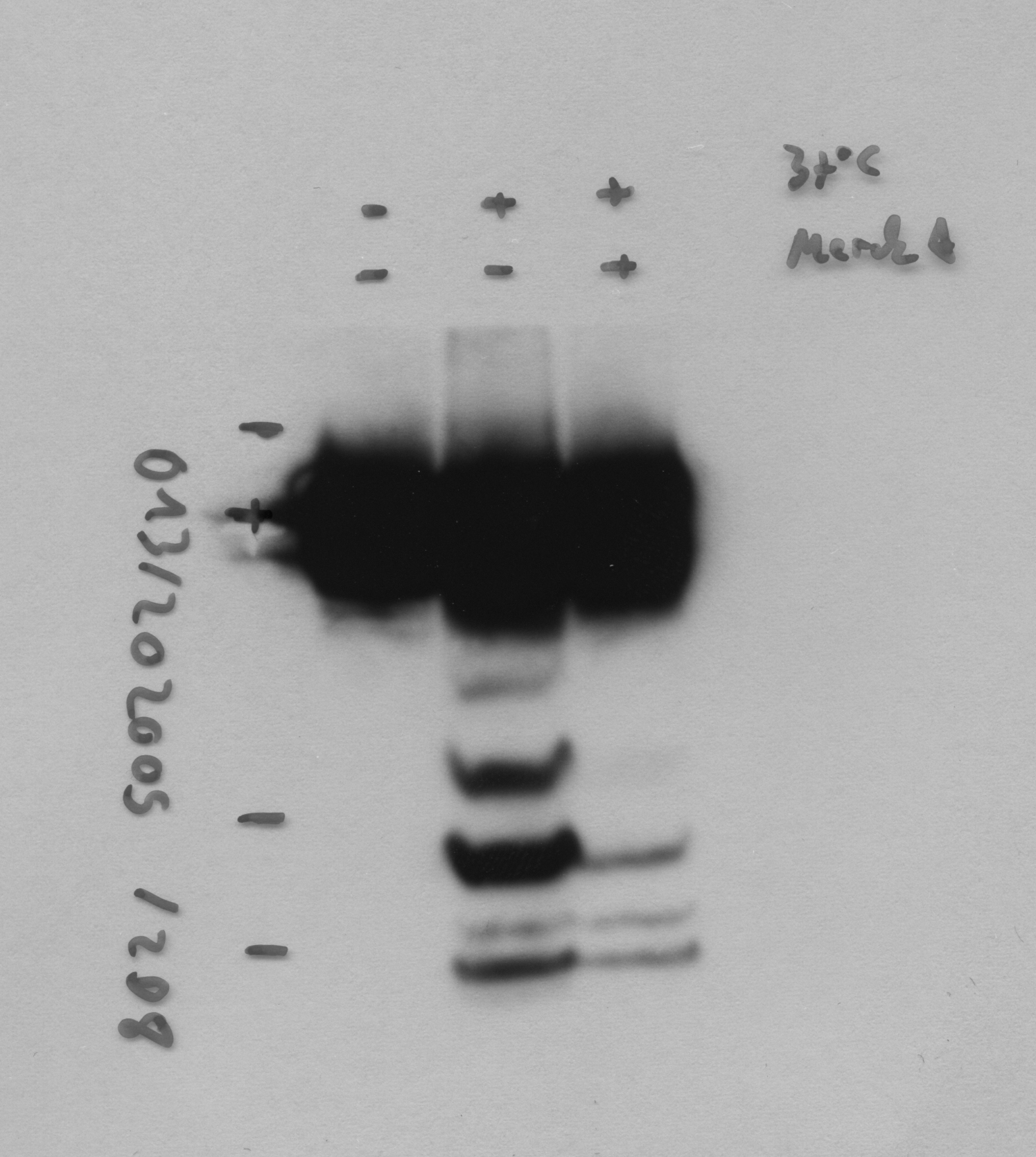

Supplement: Figure 1—source data 1. [file elife-76090-fig1-data1.zip › Figure1-source data1/Figure1C/Figure1C-Ab_species.tif]

- + + 37 °C  
- - + L-685,458

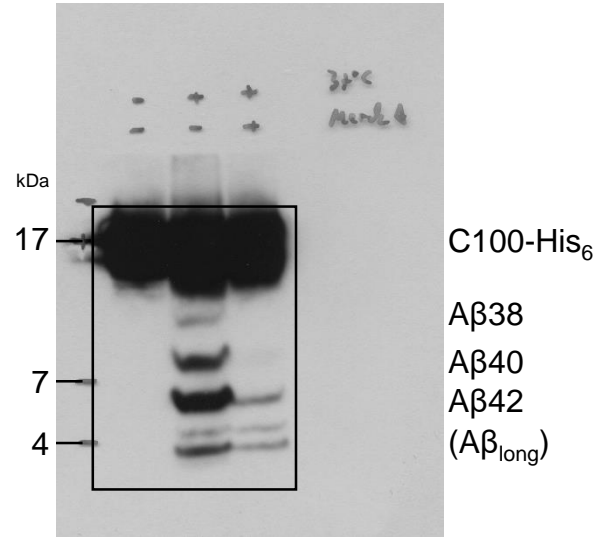

Supplement: Figure 1—source data 1. [file elife-76090-fig1-data1.zip › Figure1-source data1/Figure1C/Figure1C-annotated blots.pdf]

## Slide 1
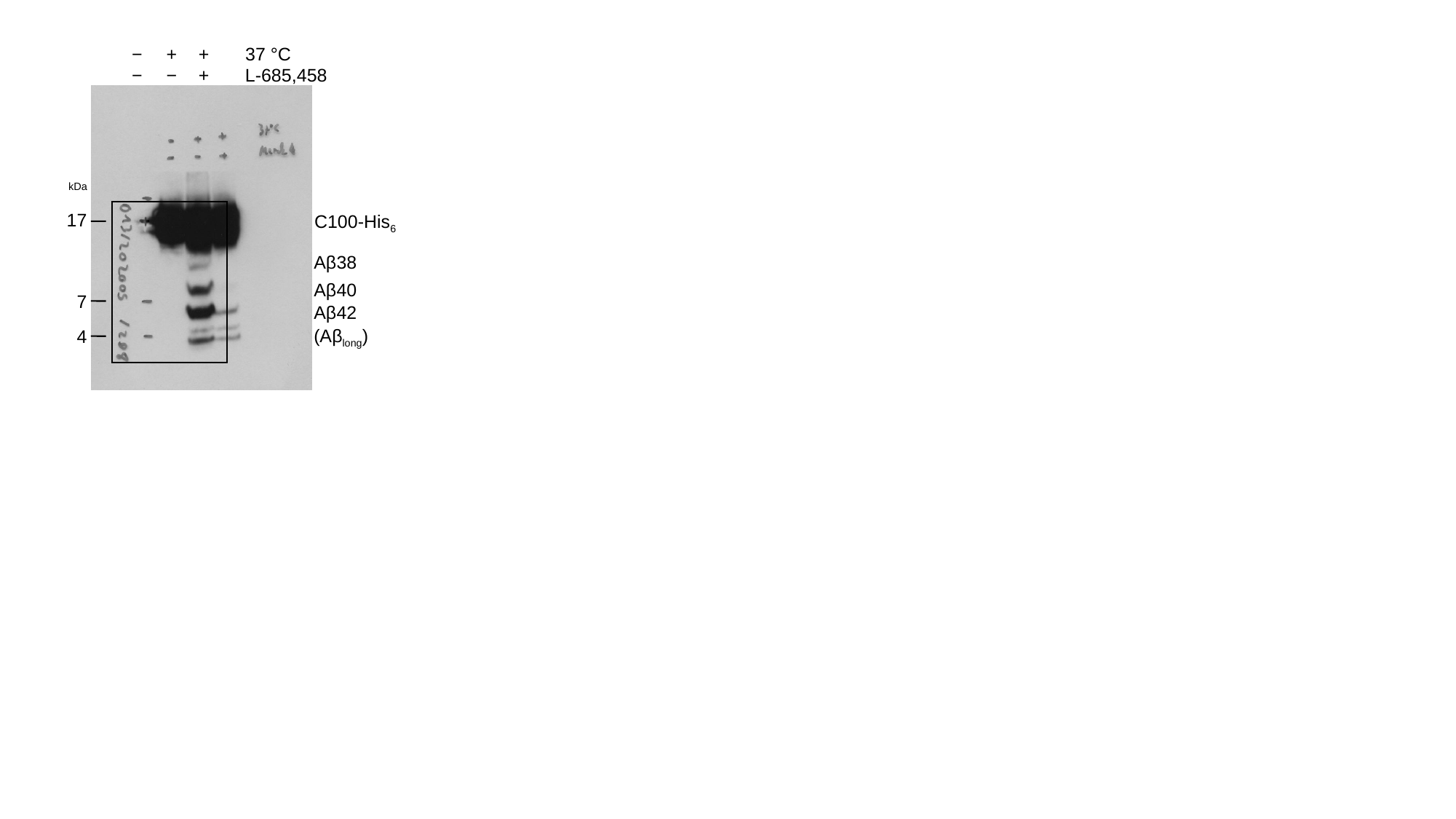

−
+
+
37 °C
−
−
+
L-685,458
kDa
17
C100-His6
Aβ38
Aβ40
7
Aβ42
(Aβlong)
4

Supplement: Figure 1—source data 1. [file elife-76090-fig1-data1.zip › Figure1-source data1/Figure1C/Figure1C-annotated blots.pptx]

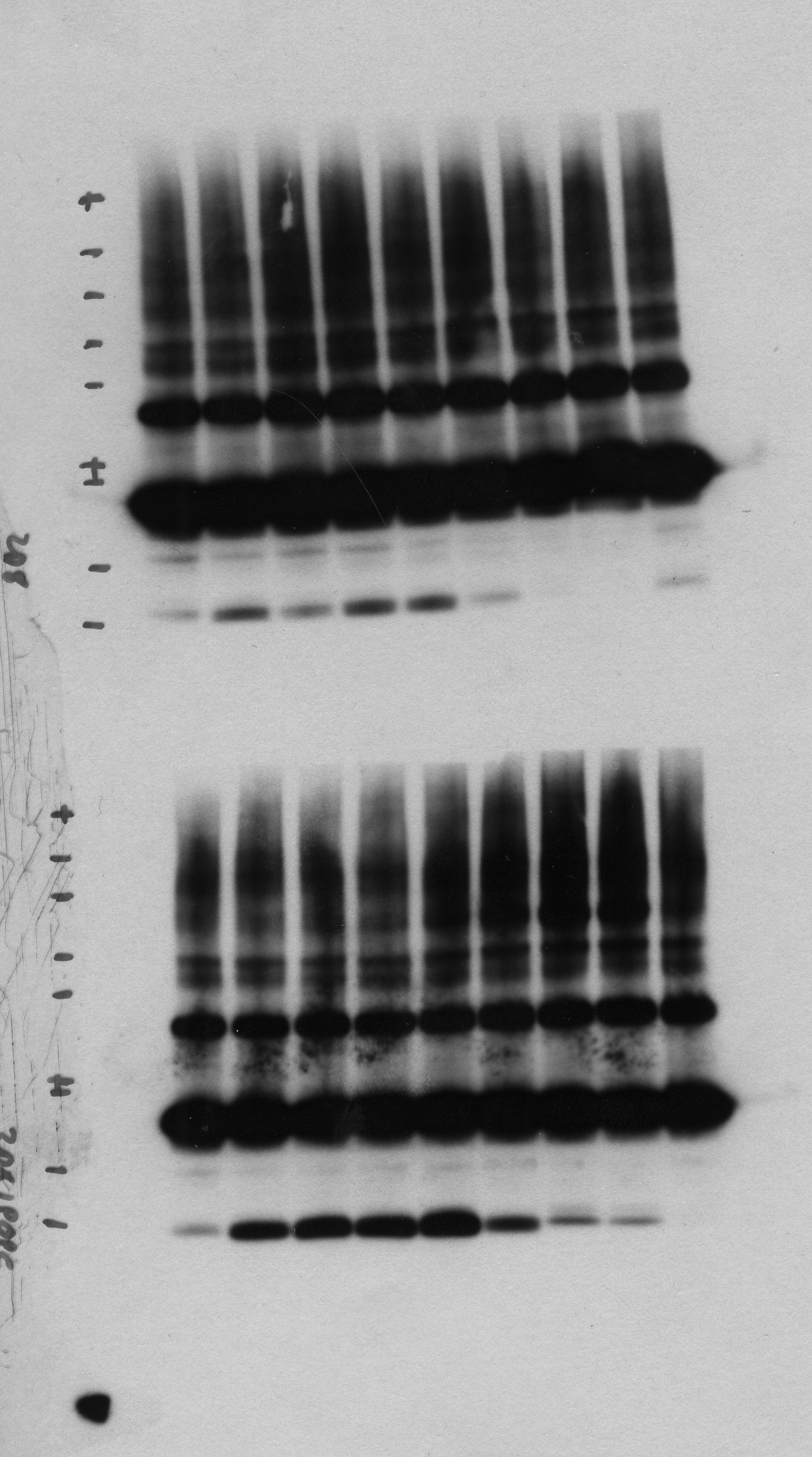

Supplement: Figure 2—source data 1. [file elife-76090-fig2-data1.zip › Figure2-source data1/Figure2A/Figure2A-Abeta.tif]

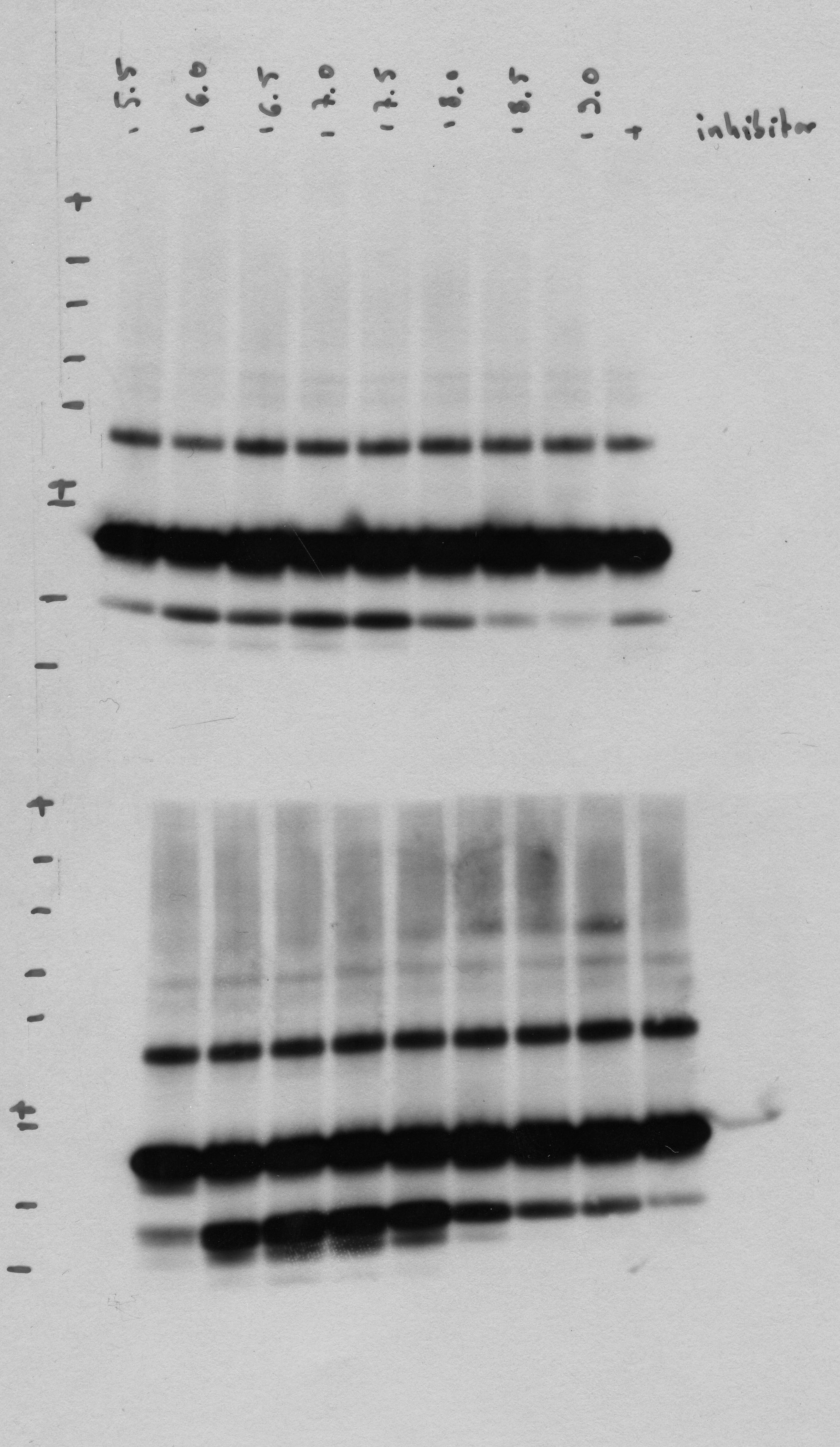

Supplement: Figure 2—source data 1. [file elife-76090-fig2-data1.zip › Figure2-source data1/Figure2A/Figure2A-AICD.tif]

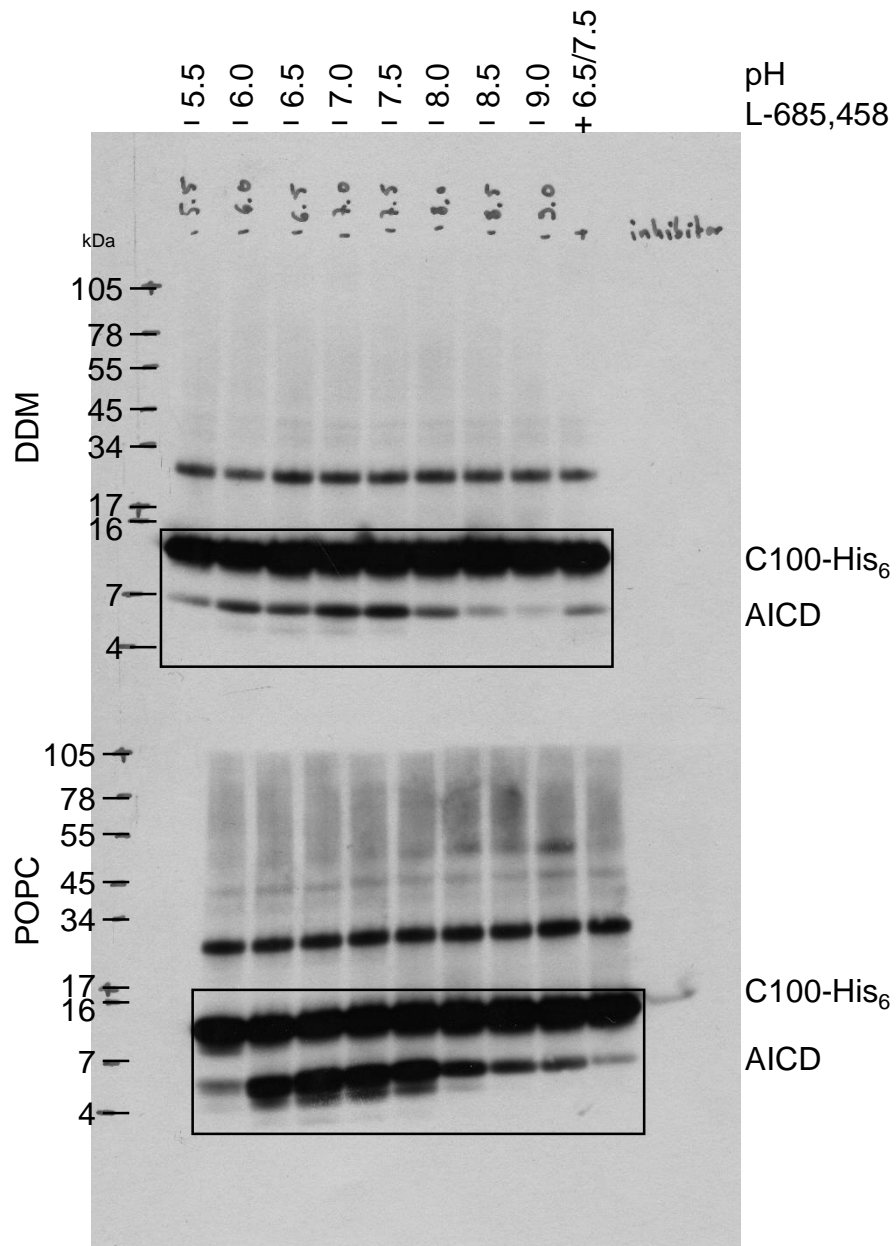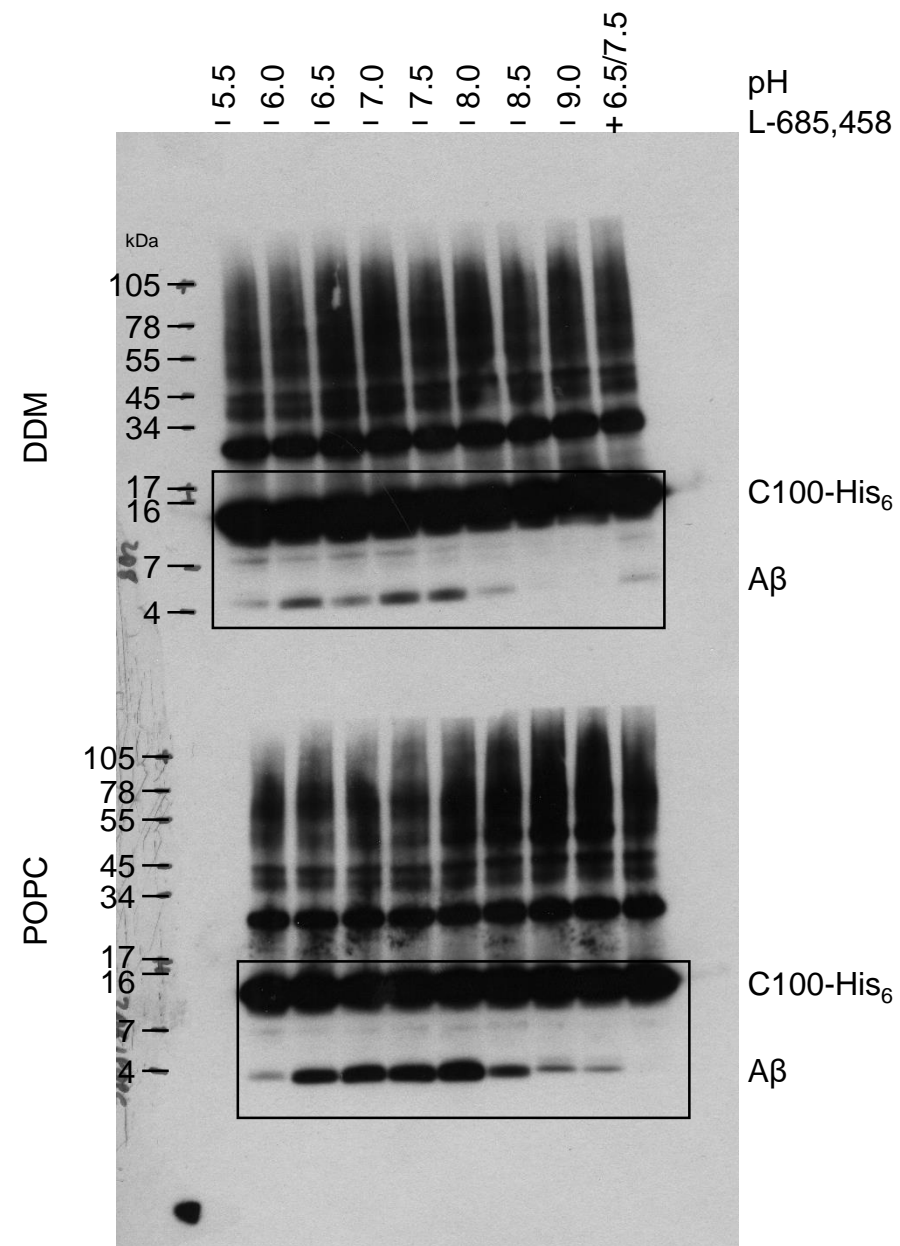

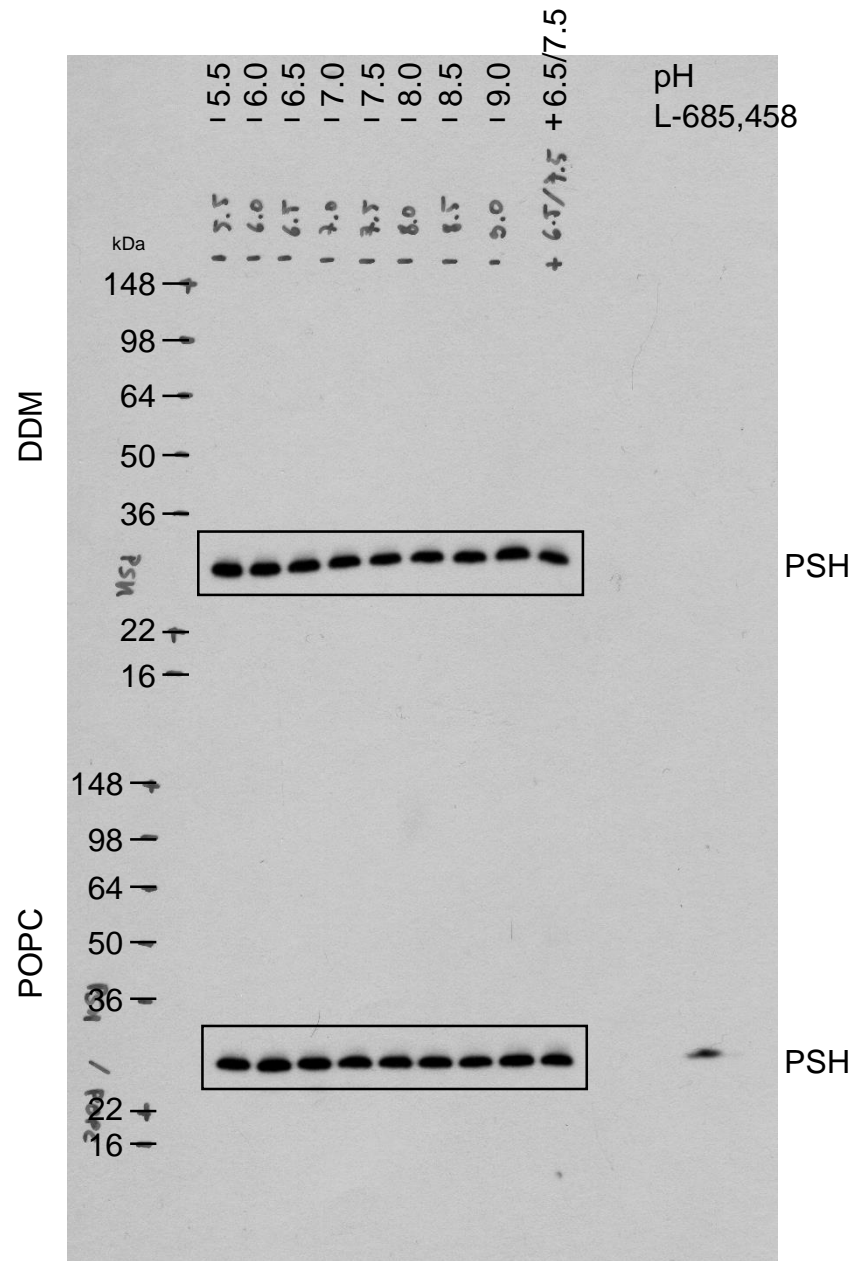

Supplement: Figure 2—source data 1. [file elife-76090-fig2-data1.zip › Figure2-source data1/Figure2A/Figure2A-annotated blots.pdf]

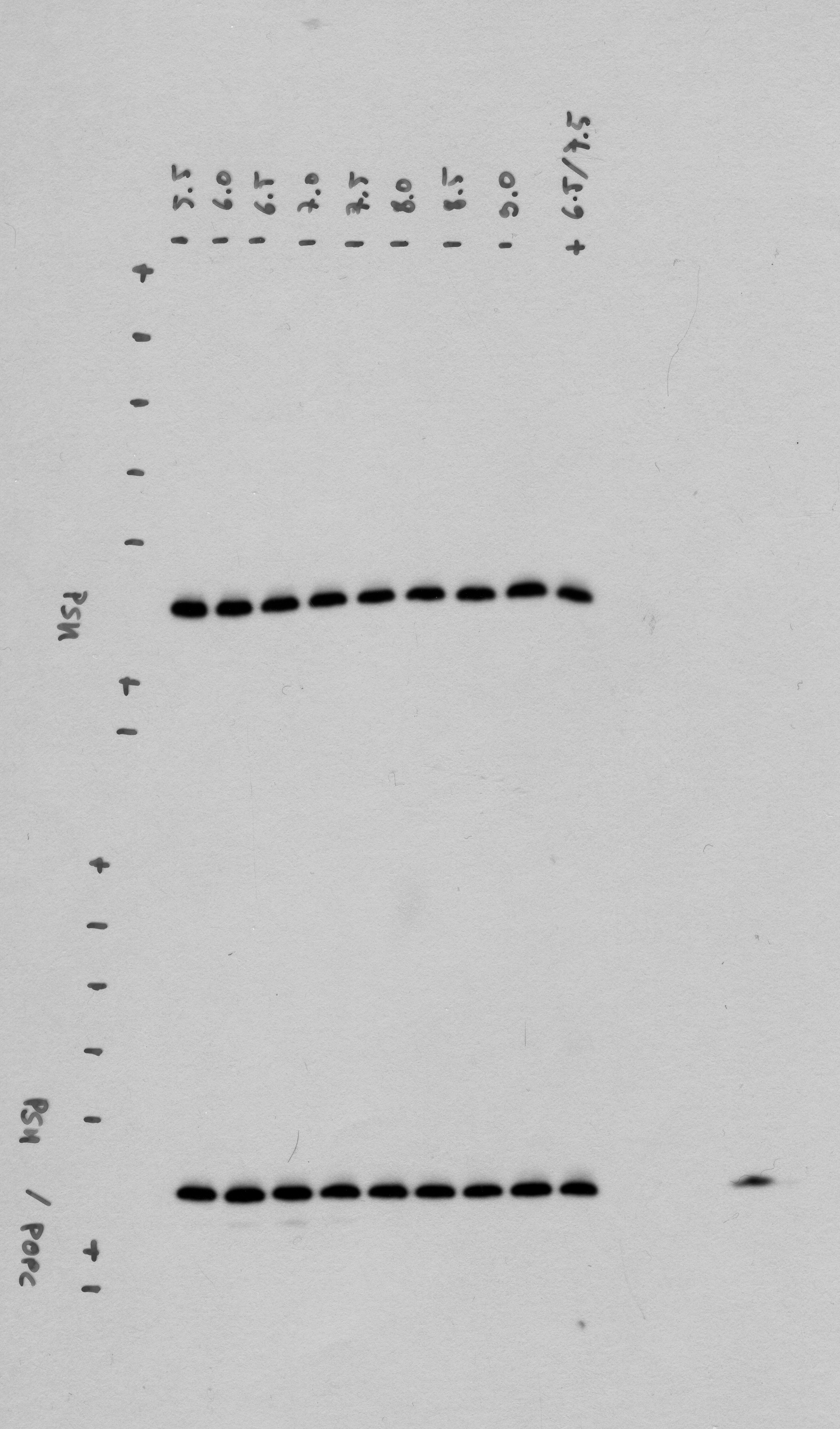

Supplement: Figure 2—source data 1. [file elife-76090-fig2-data1.zip › Figure2-source data1/Figure2A/Figure2A-PSH.tif]

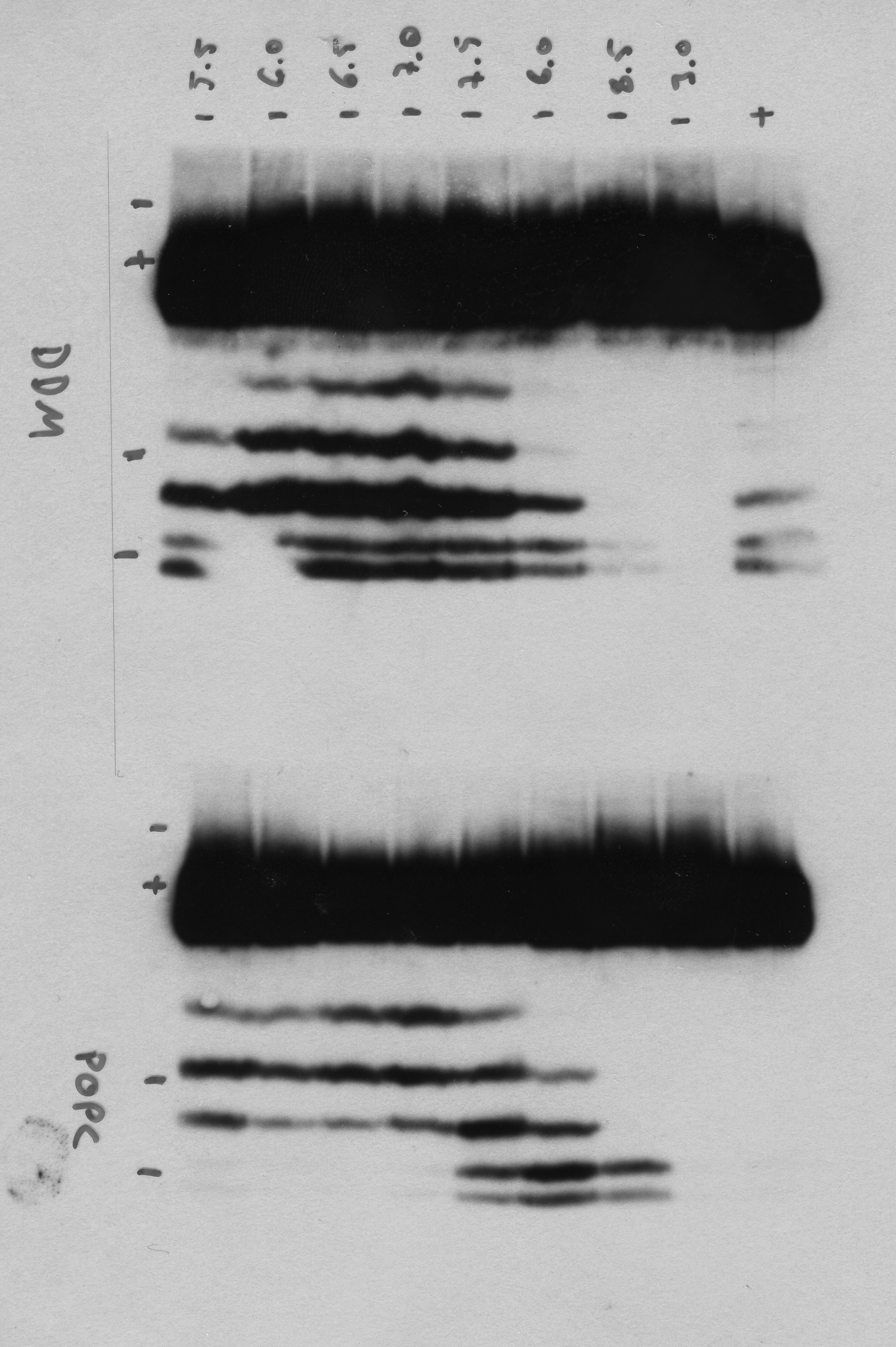

Supplement: Figure 2—source data 1. [file elife-76090-fig2-data1.zip › Figure2-source data1/Figure2B/Figure2B-Abeta_species.tif]

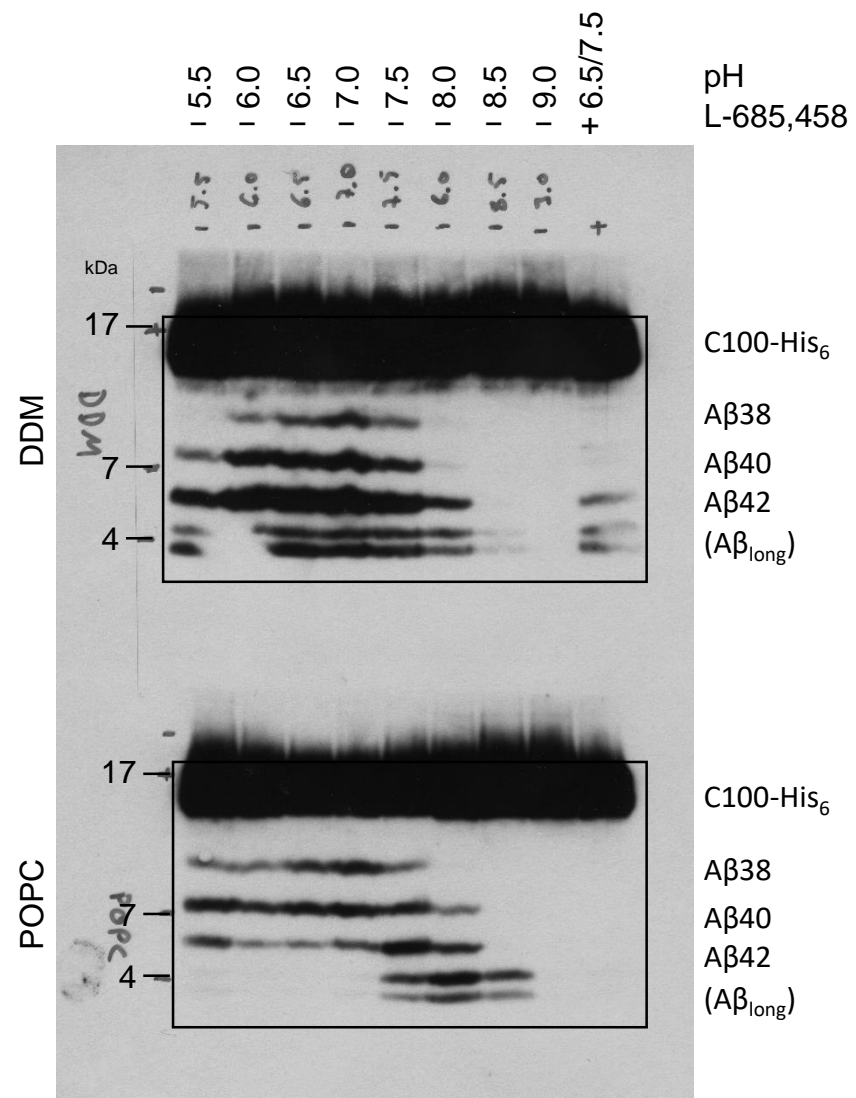

Supplement: Figure 2—source data 1. [file elife-76090-fig2-data1.zip › Figure2-source data1/Figure2B/Figure2B-annotated blots.pdf]

## Slide 1
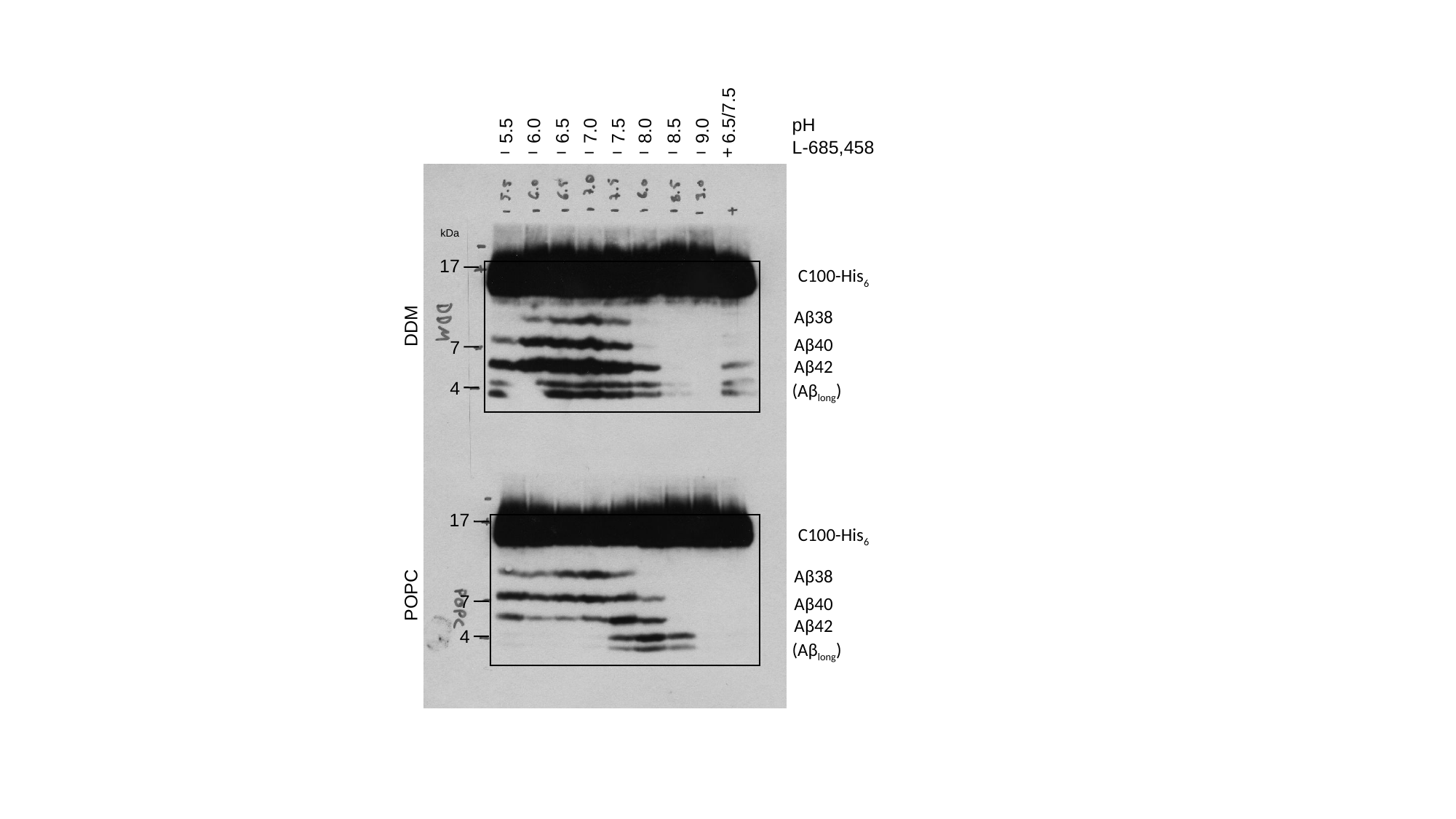

6.5/7.5
pH
5.5
6.0
6.5
7.0
7.5
8.0
8.5
9.0
−
−
−
−
−
−
−
−
+
L-685,458
kDa
17
C100-His6
Aβ38
DDM
Aβ40
7
Aβ42
4
(Aβlong)
17
C100-His6
Aβ38
POPC
7
Aβ40
Aβ42
4
(Aβlong)

Supplement: Figure 2—source data 1. [file elife-76090-fig2-data1.zip › Figure2-source data1/Figure2B/Figure2B-annotated blots.pptx]

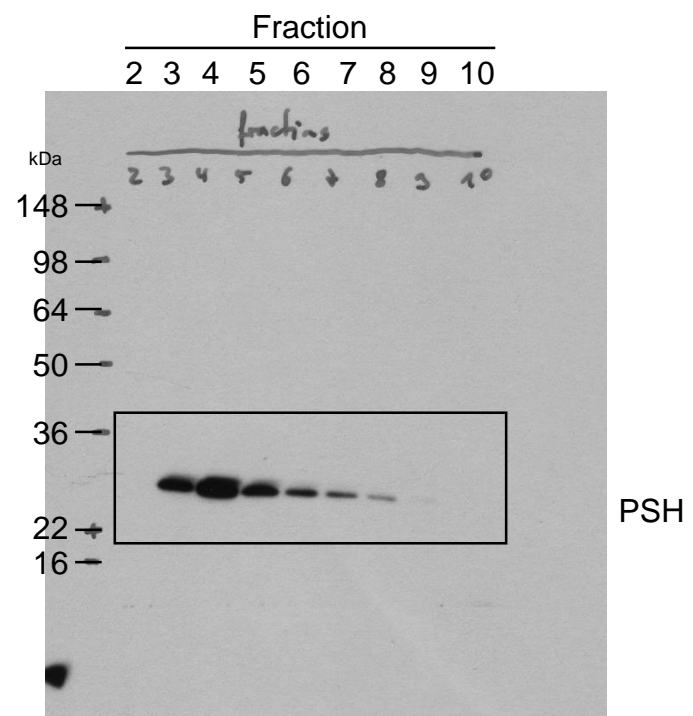

Supplement: Figure 2—figure supplement 1—source data 2. [file elife-76090-fig2-figsupp1-data2.zip › Figure2-figure supplement1-source data2/Figure2-figure supplement1B-annotated blots.pdf]

## Slide 1
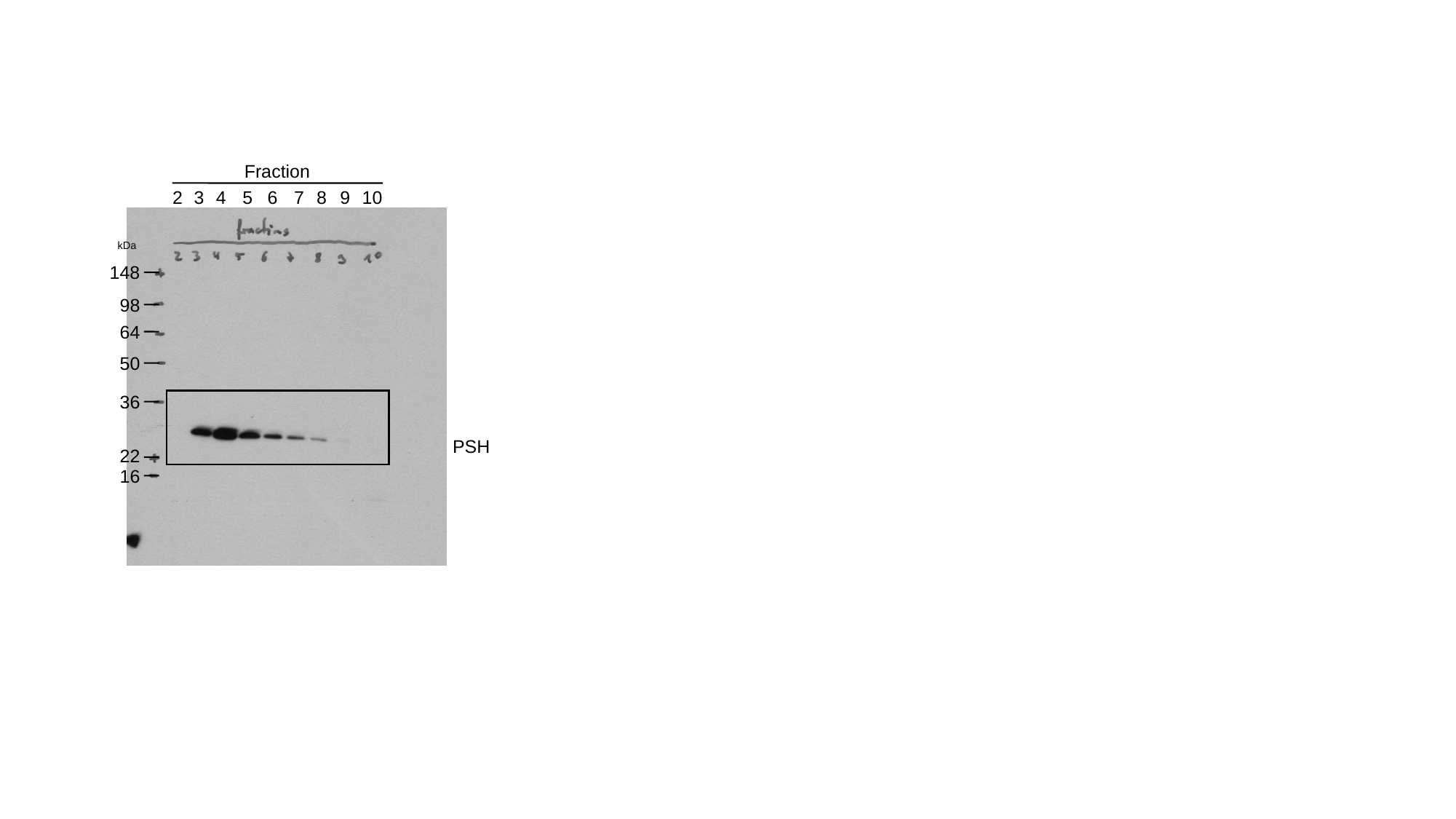

Fraction
2
3
4
5
6
7
8
9
10
kDa
148
98
64
50
36
PSH
22
16

Supplement: Figure 2—figure supplement 1—source data 2. [file elife-76090-fig2-figsupp1-data2.zip › Figure2-figure supplement1-source data2/Figure2-figure supplement1B-annotated blots.pptx]

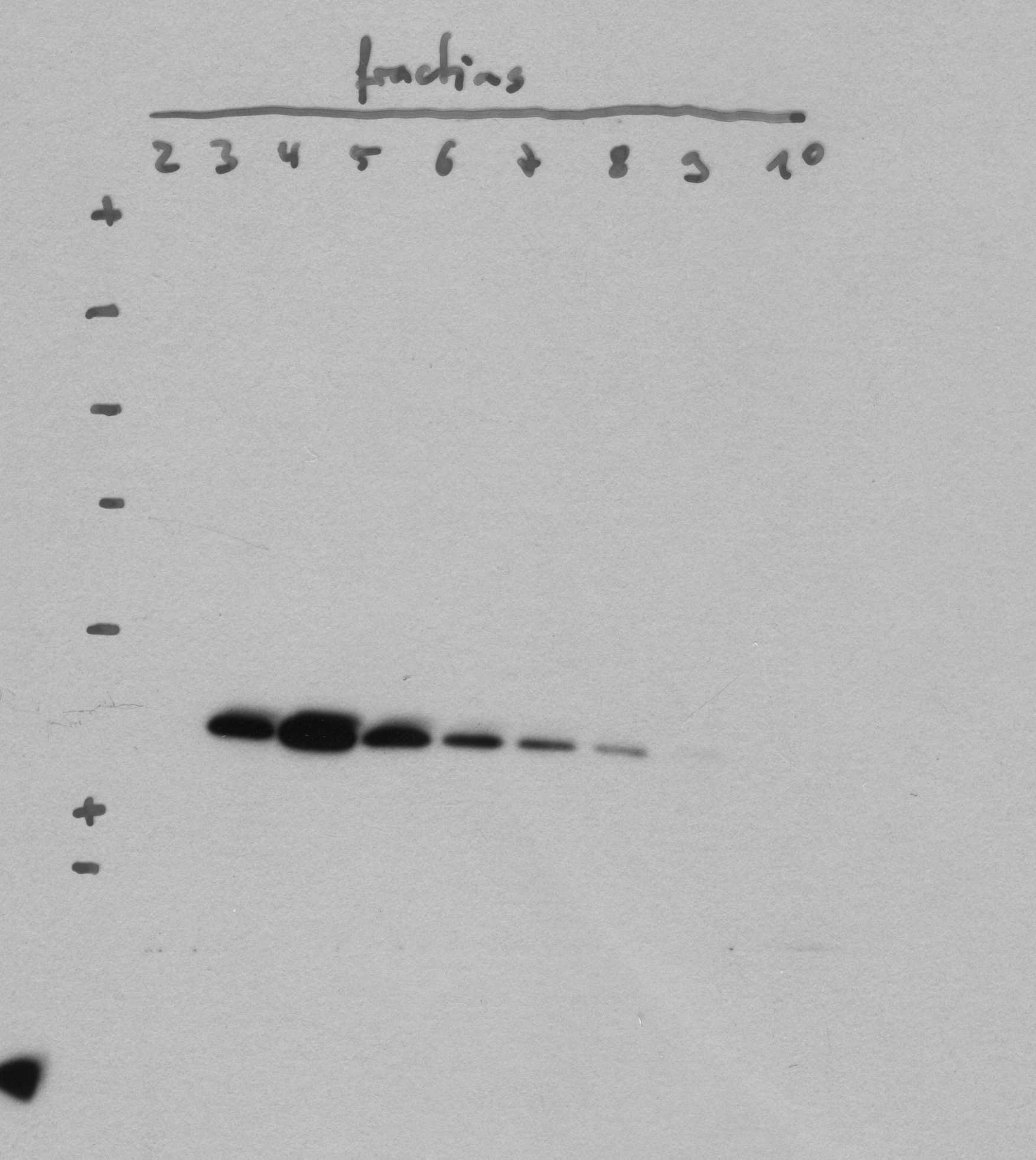

Supplement: Figure 2—figure supplement 1—source data 2. [file elife-76090-fig2-figsupp1-data2.zip › Figure2-figure supplement1-source data2/Figure2-figure supplement1B-PSH.tif]

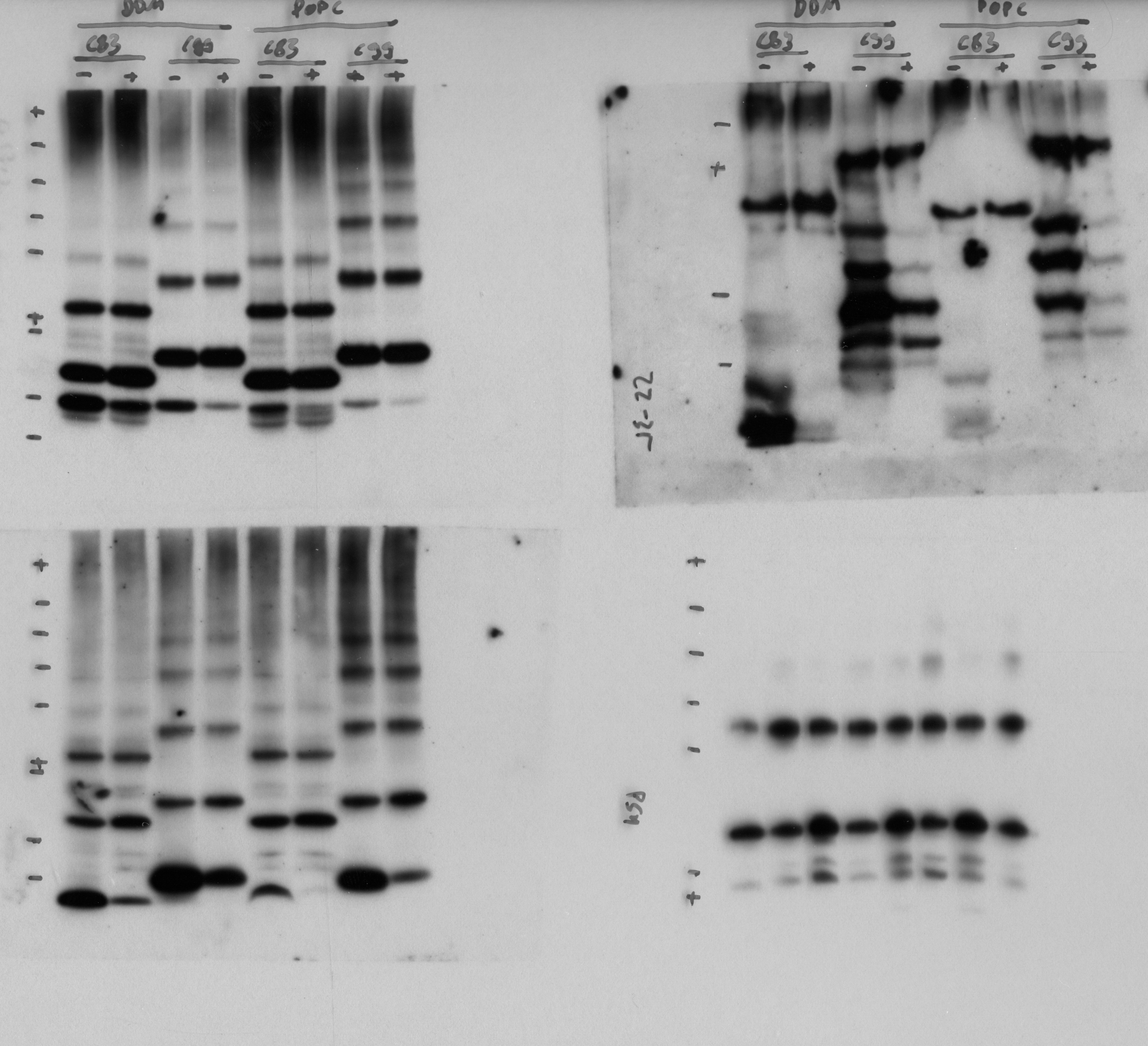

Supplement: Figure 3—source data 1. [file elife-76090-fig3-data1.zip › Figure3-source data1/Figure3A-Abeta.tif]

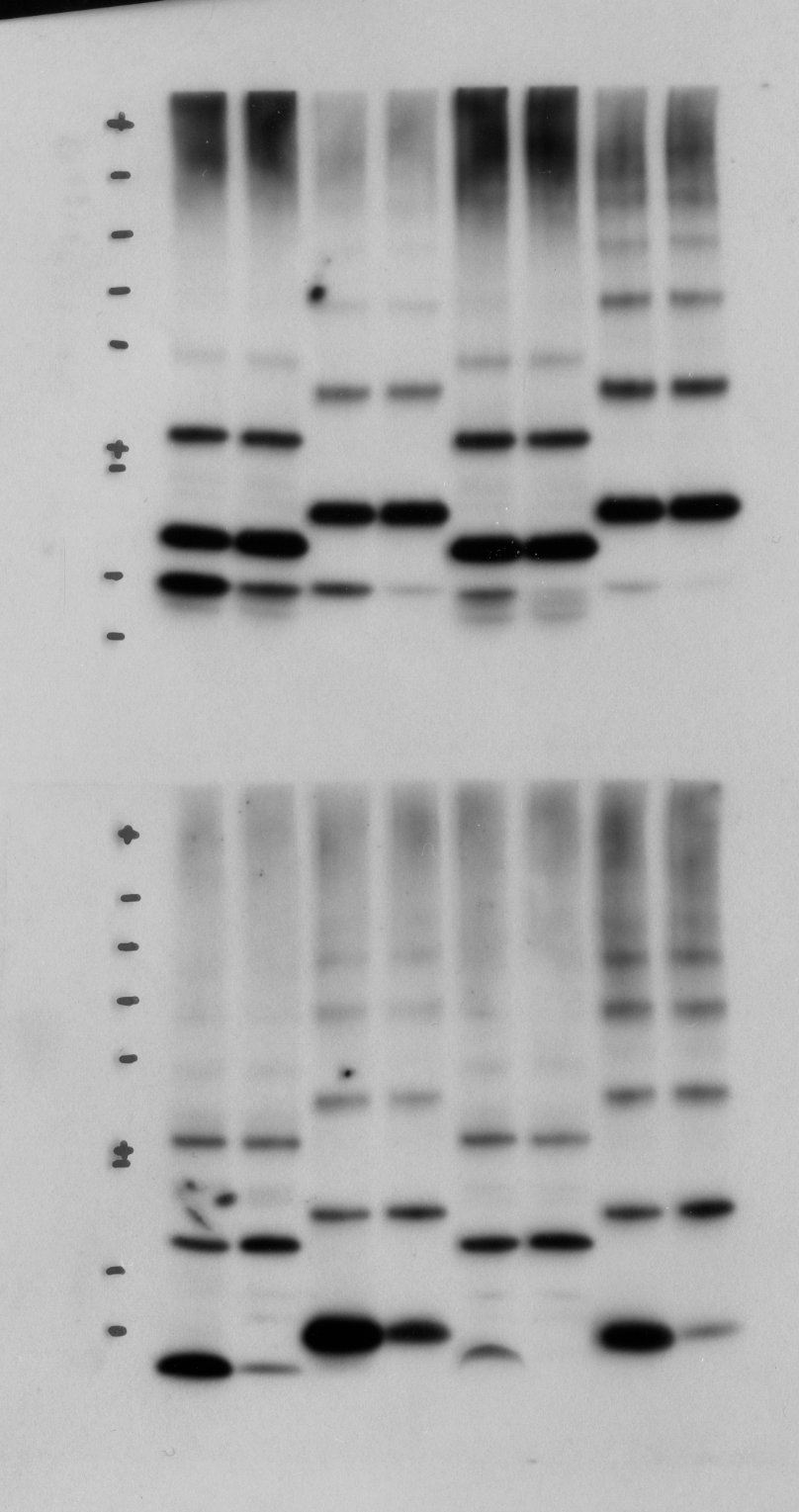

Supplement: Figure 3—source data 1. [file elife-76090-fig3-data1.zip › Figure3-source data1/Figure3A-AICD.tif]

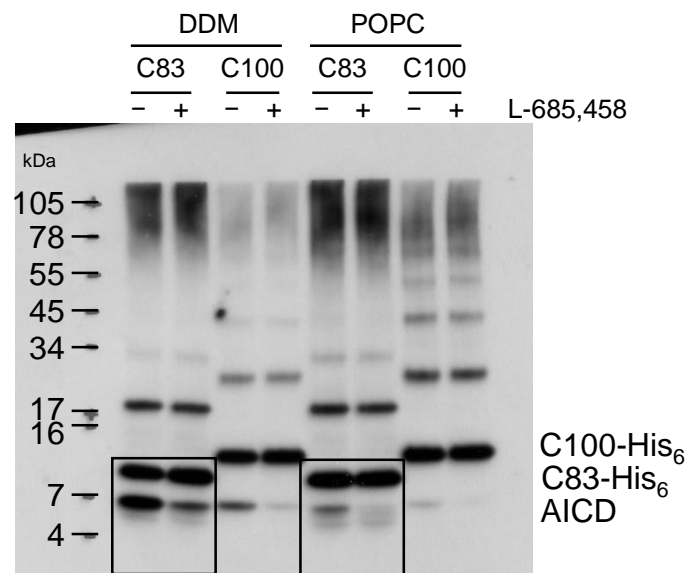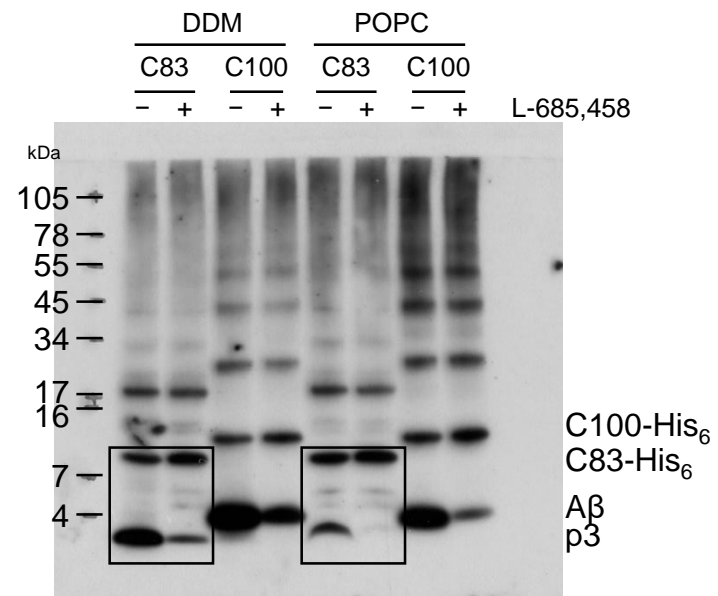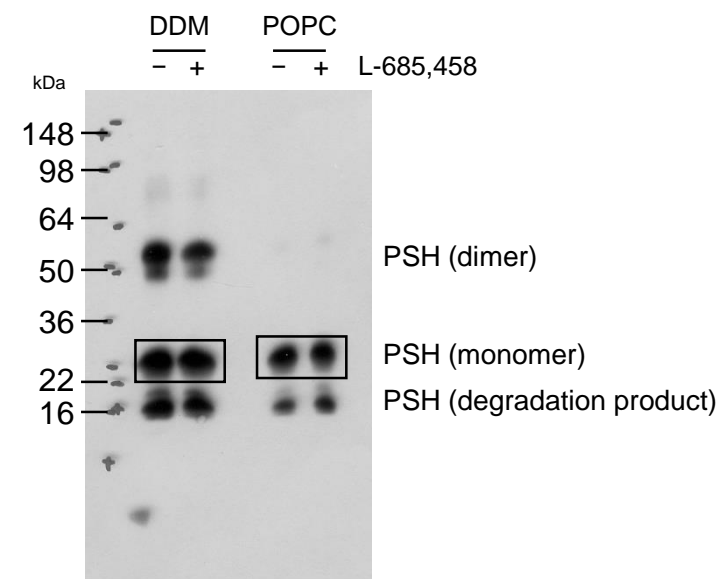

Supplement: Figure 3—source data 1. [file elife-76090-fig3-data1.zip › Figure3-source data1/Figure3A-annotated blots.pdf]

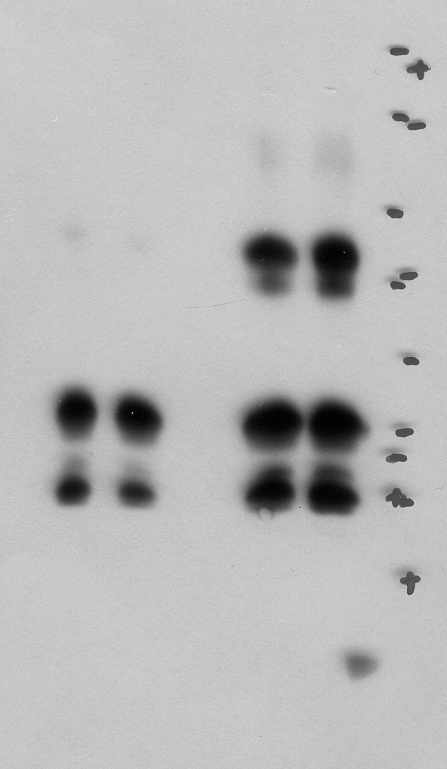

Supplement: Figure 3—source data 1. [file elife-76090-fig3-data1.zip › Figure3-source data1/Figure3A-PSH.tif]

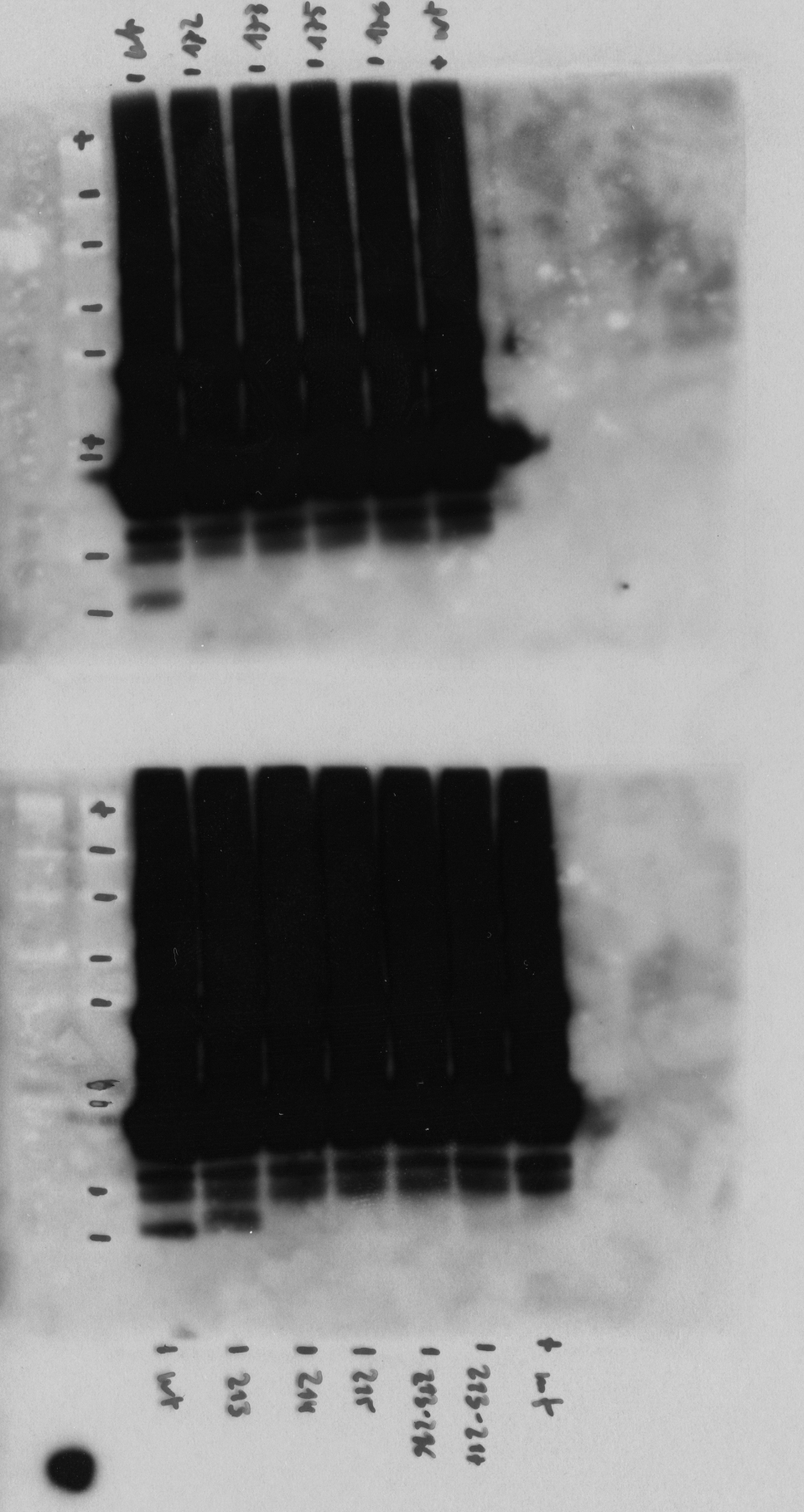

Supplement: Figure 4—source data 1. [file elife-76090-fig4-data1.zip › Figure4-source data1/Figure4E-Abeta-POPC-DDM.tif]

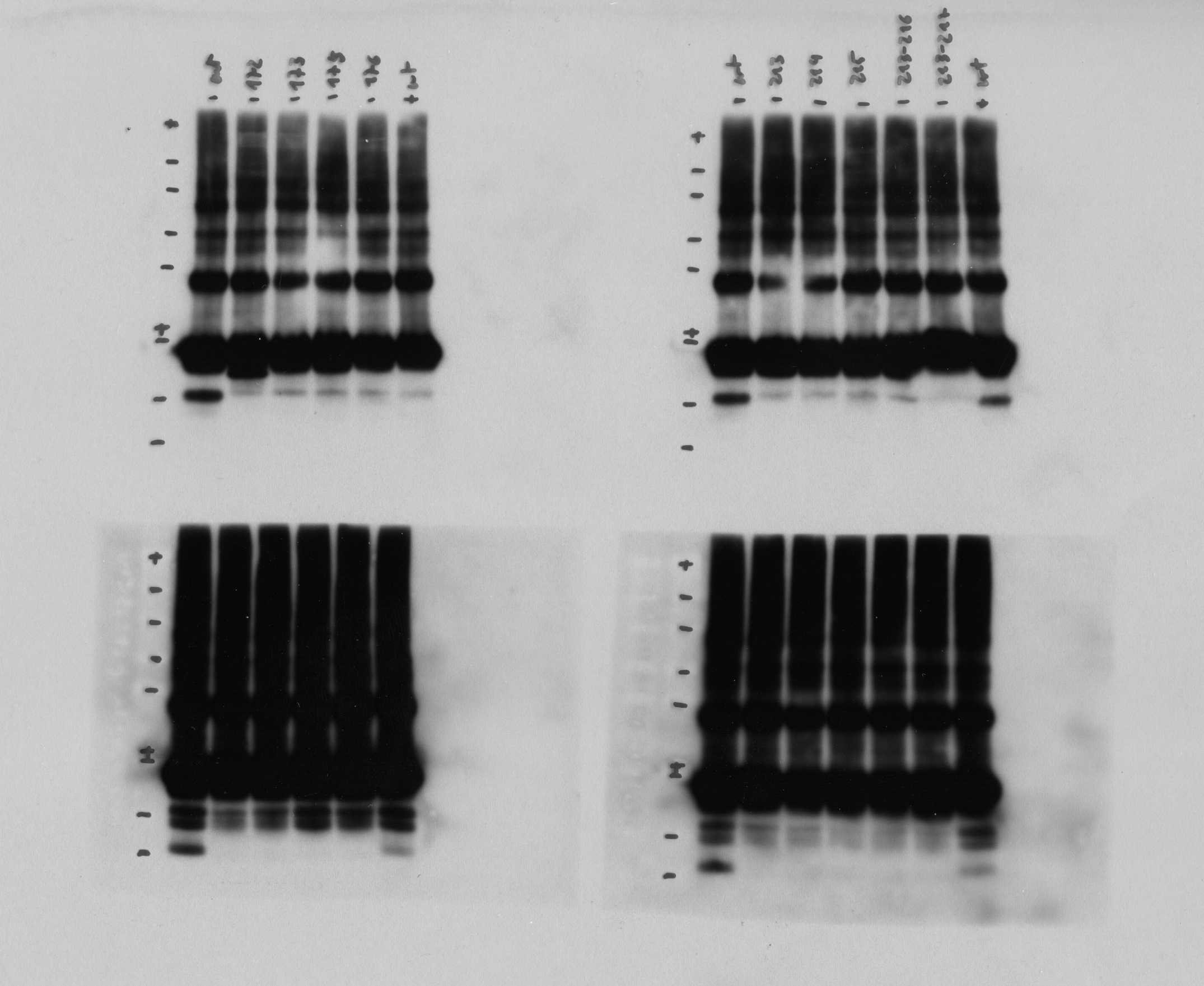

Supplement: Figure 4—source data 1. [file elife-76090-fig4-data1.zip › Figure4-source data1/Figure4E-AICD-Abeta-DDM.tif]

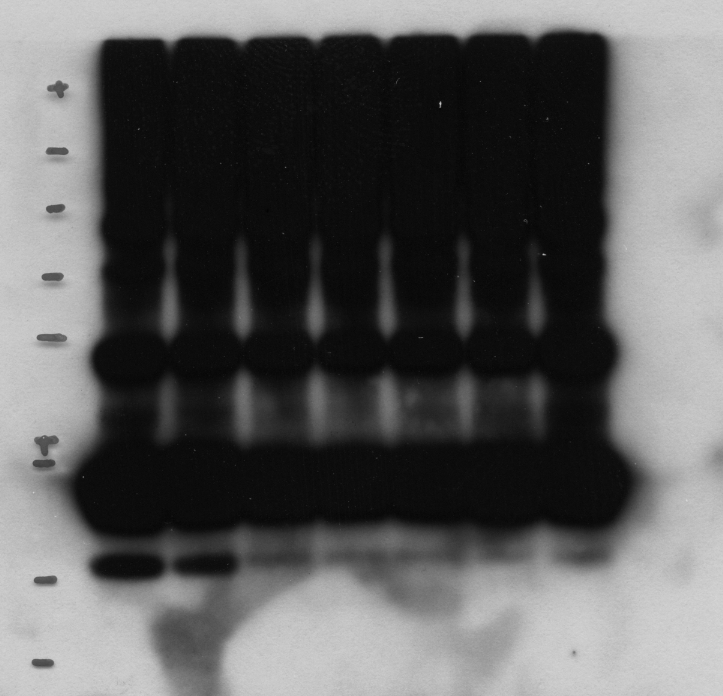

Supplement: Figure 4—source data 1. [file elife-76090-fig4-data1.zip › Figure4-source data1/Figure4E-AICD-POPC.tif]

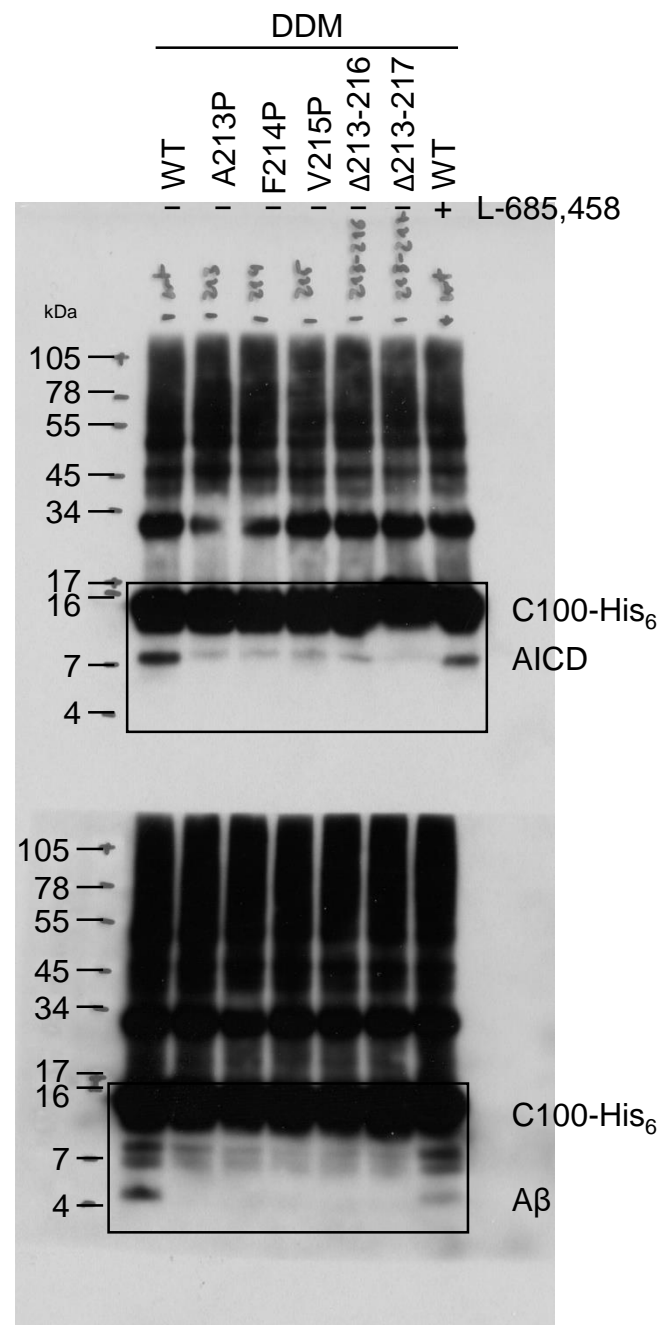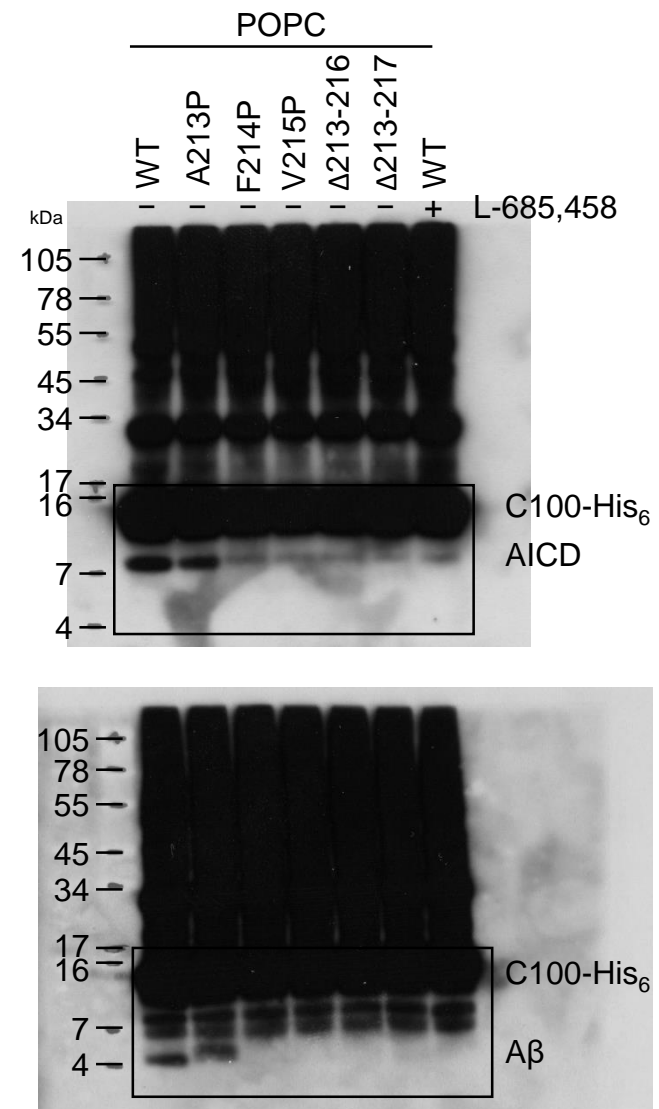

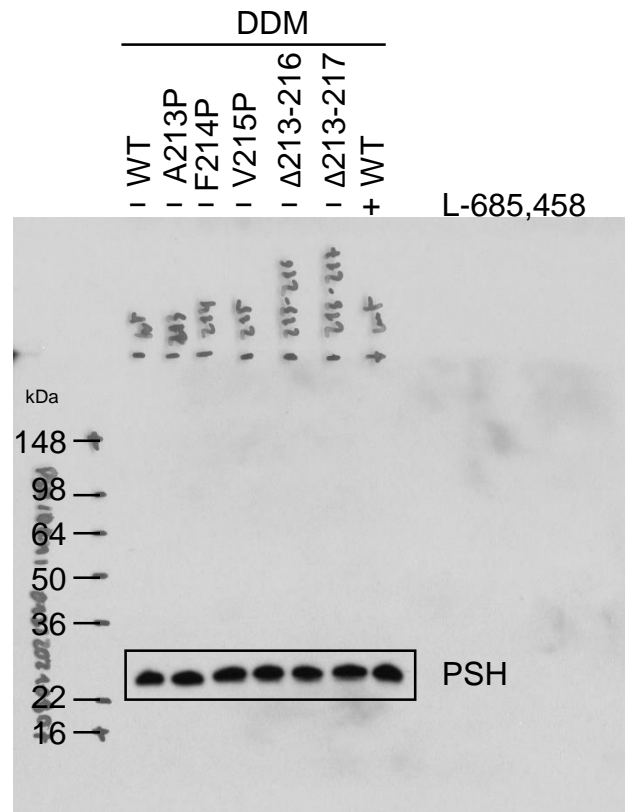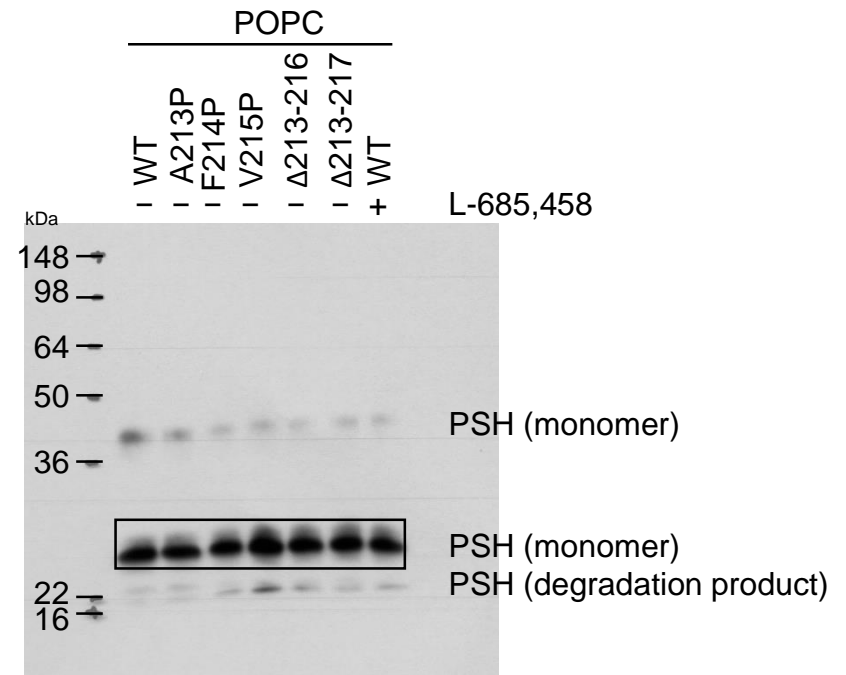

Supplement: Figure 4—source data 1. [file elife-76090-fig4-data1.zip › Figure4-source data1/Figure4E-annotated blots.pdf]

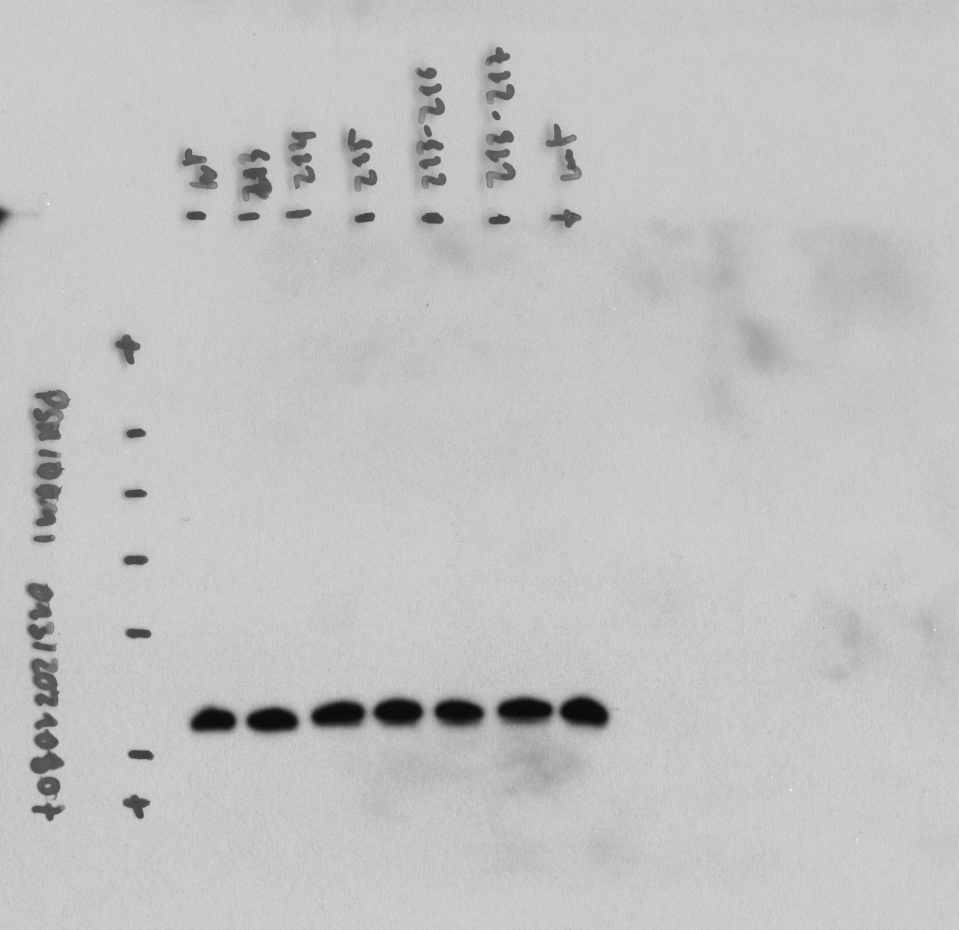

Supplement: Figure 4—source data 1. [file elife-76090-fig4-data1.zip › Figure4-source data1/Figure4E-PSH-DDM.tif]

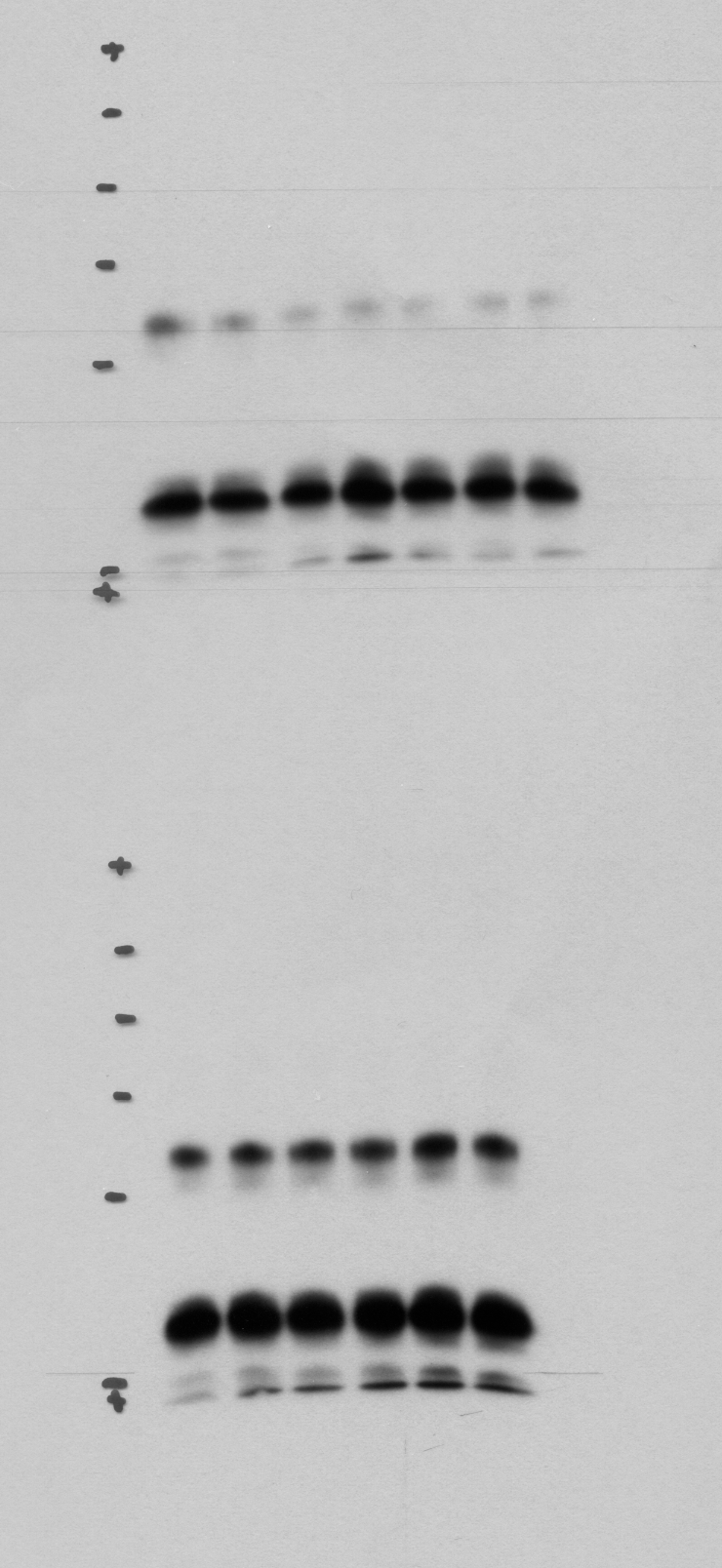

Supplement: Figure 4—source data 1. [file elife-76090-fig4-data1.zip › Figure4-source data1/Figure4E-PSH-POPC.tif]

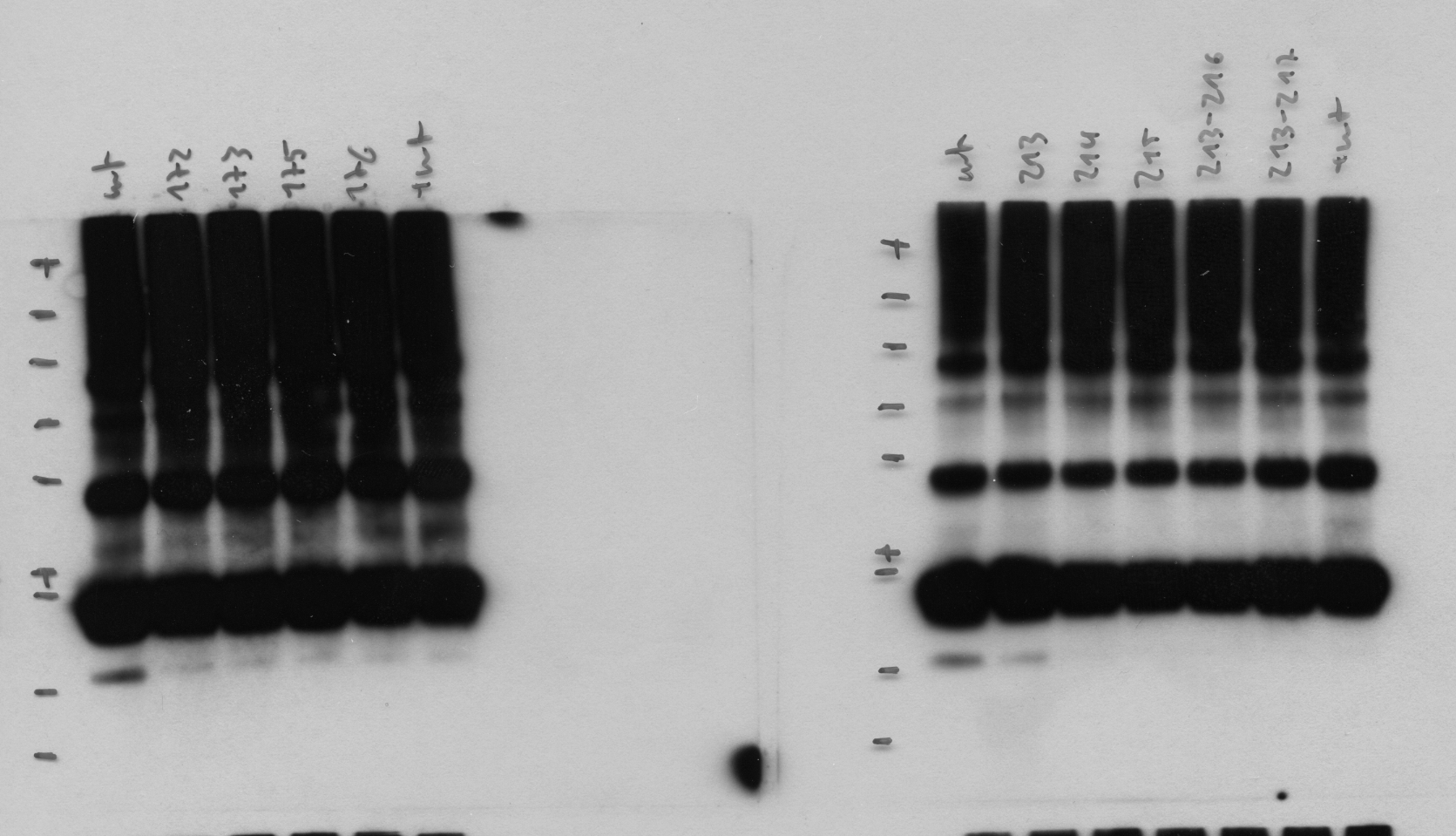

Supplement: Figure 7—source data 2. [file elife-76090-fig7-data2.zip › Figure7-source data2/Figure7F-AICD-POPC.tif]

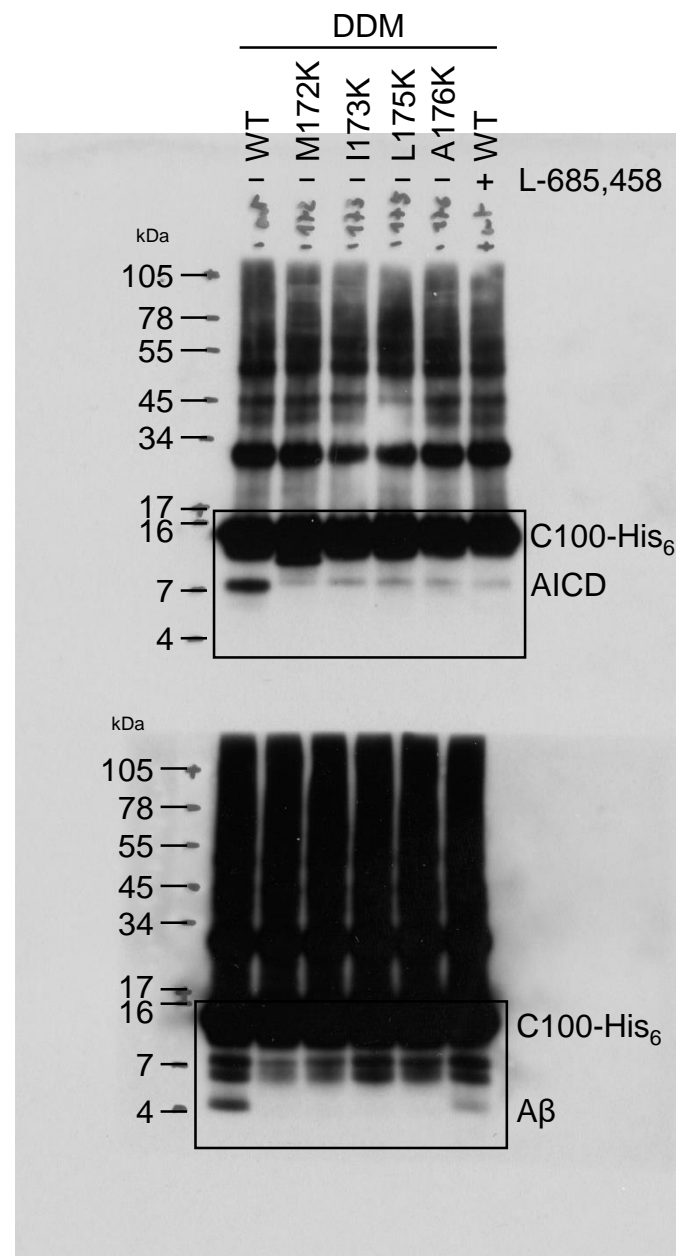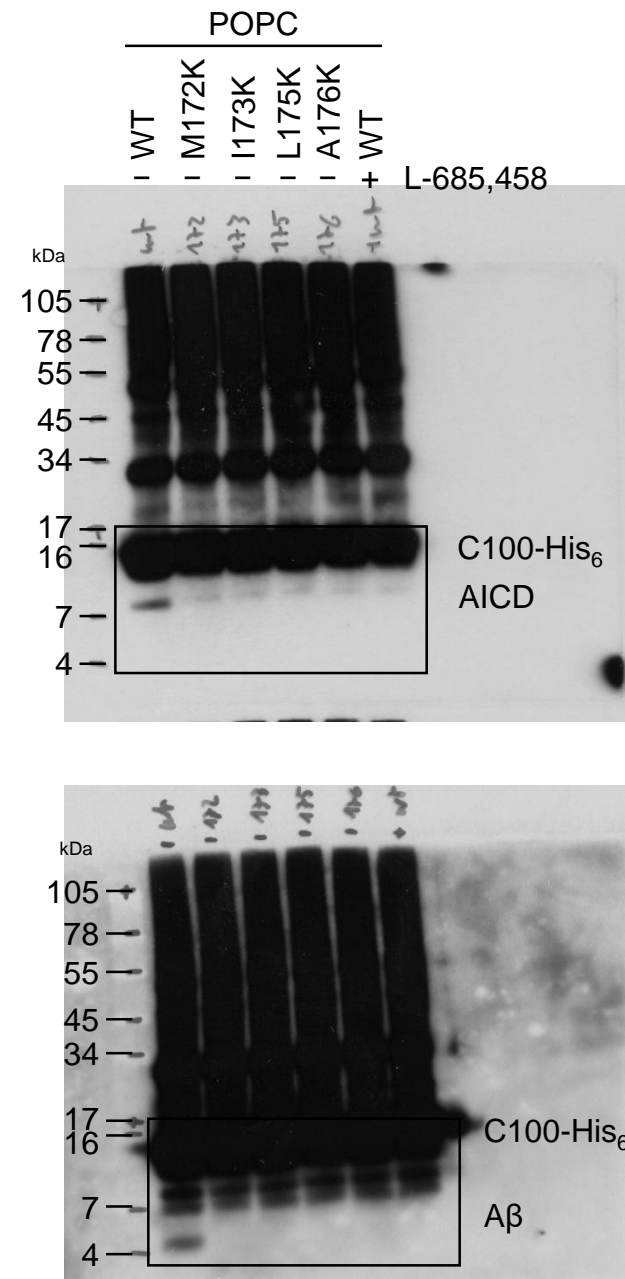

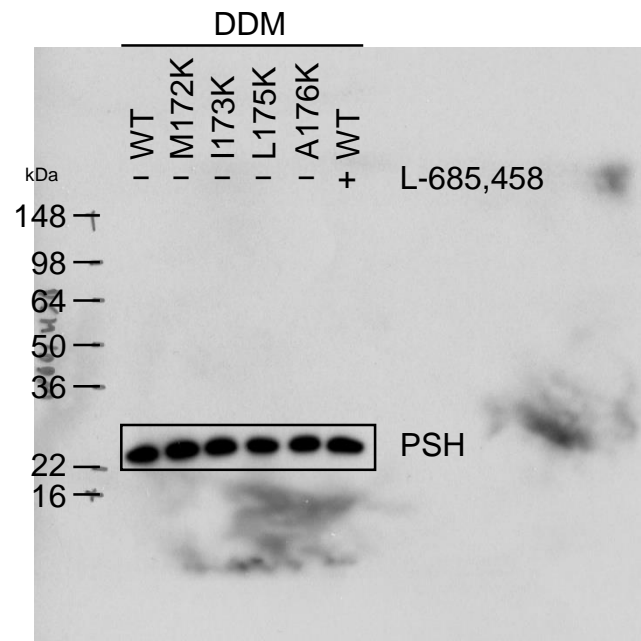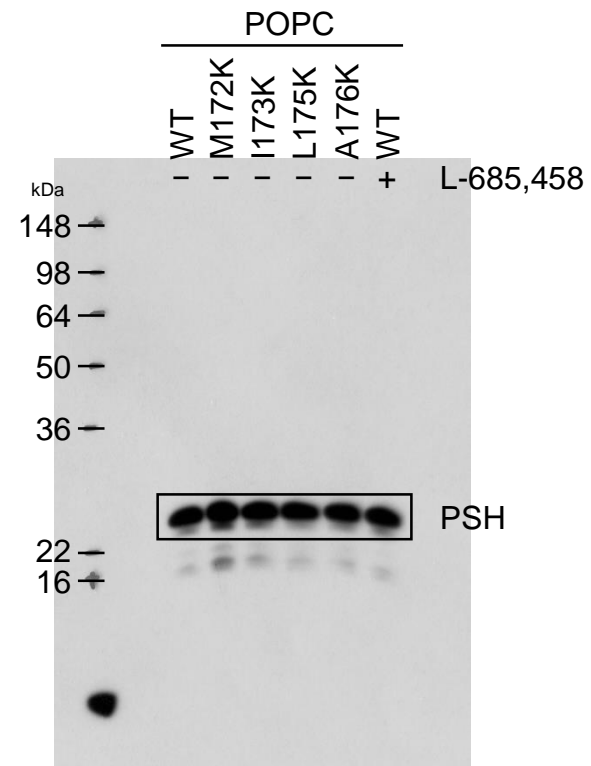

Supplement: Figure 7—source data 2. [file elife-76090-fig7-data2.zip › Figure7-source data2/Figure7F-annotated blots.pdf]

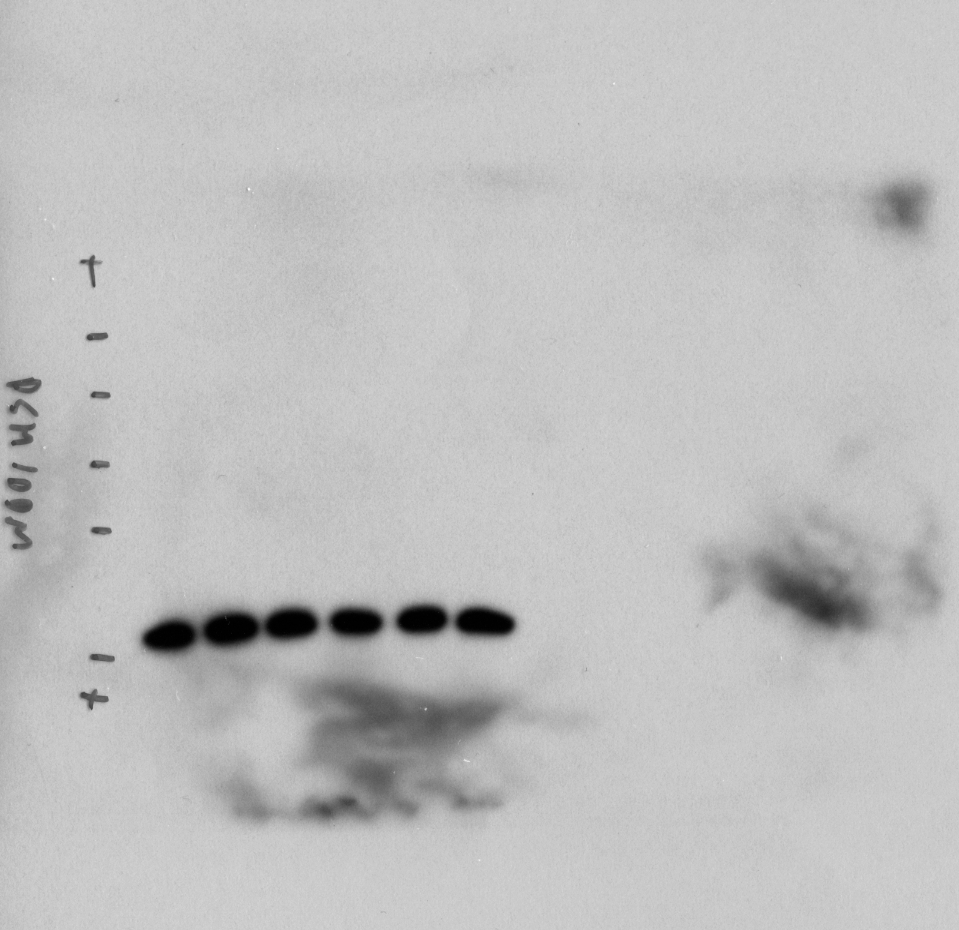

Supplement: Figure 7—source data 2. [file elife-76090-fig7-data2.zip › Figure7-source data2/Figure7F-PSH-DDM.tif]

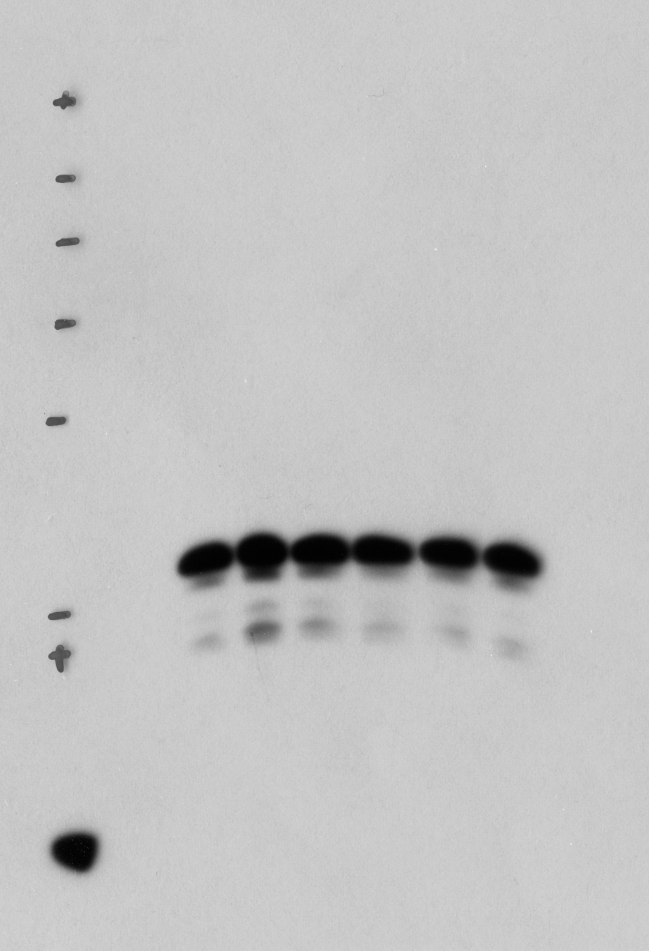

Supplement: Figure 7—source data 2. [file elife-76090-fig7-data2.zip › Figure7-source data2/Figure7F-PSH-POPC.tif]

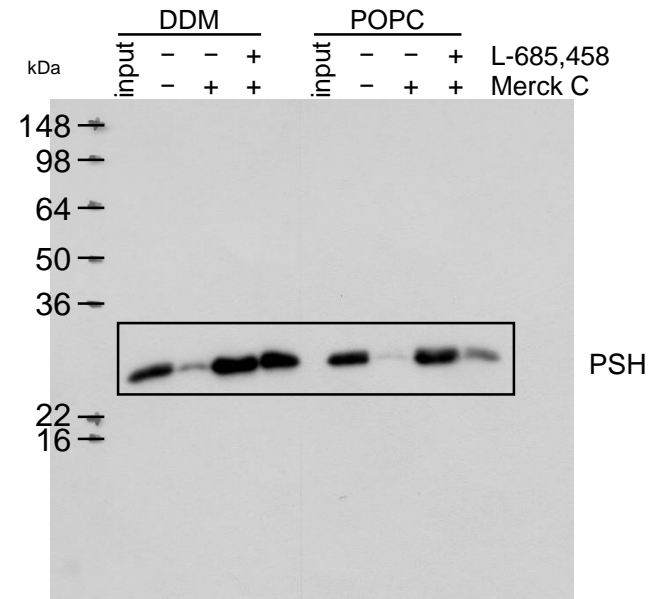

Supplement: Figure 8—source data 2. [file elife-76090-fig8-data2.zip › Figure8-source data2/Figure8C/Figure8C-annotated blots.pdf]

## Slide 1
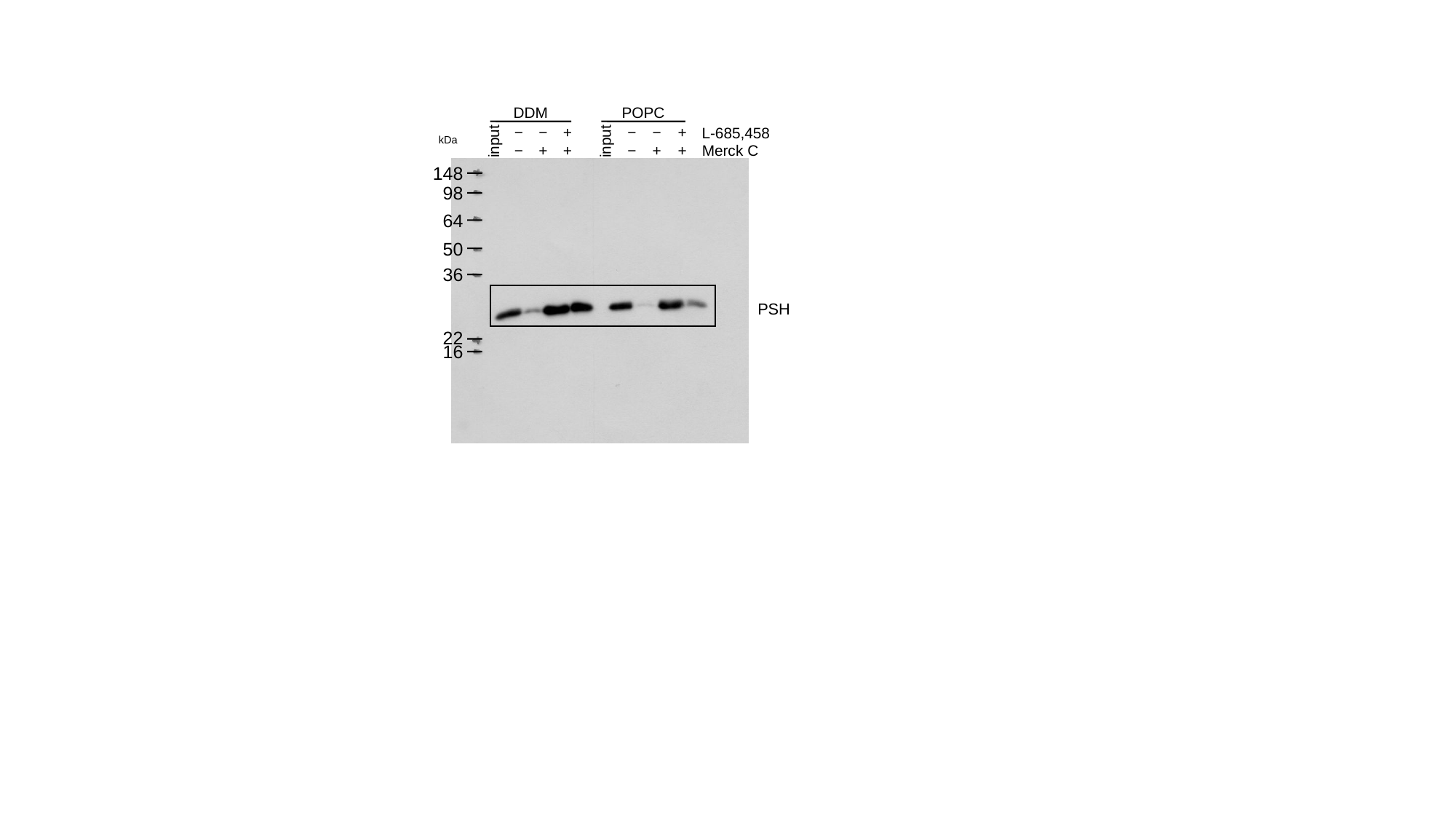

DDM
POPC
−
−
+
−
−
+
L-685,458
kDa
input
input
−
+
+
−
+
+
Merck C
148
98
64
50
36
PSH
22
16

Supplement: Figure 8—source data 2. [file elife-76090-fig8-data2.zip › Figure8-source data2/Figure8C/Figure8C-annotated blots.pptx]

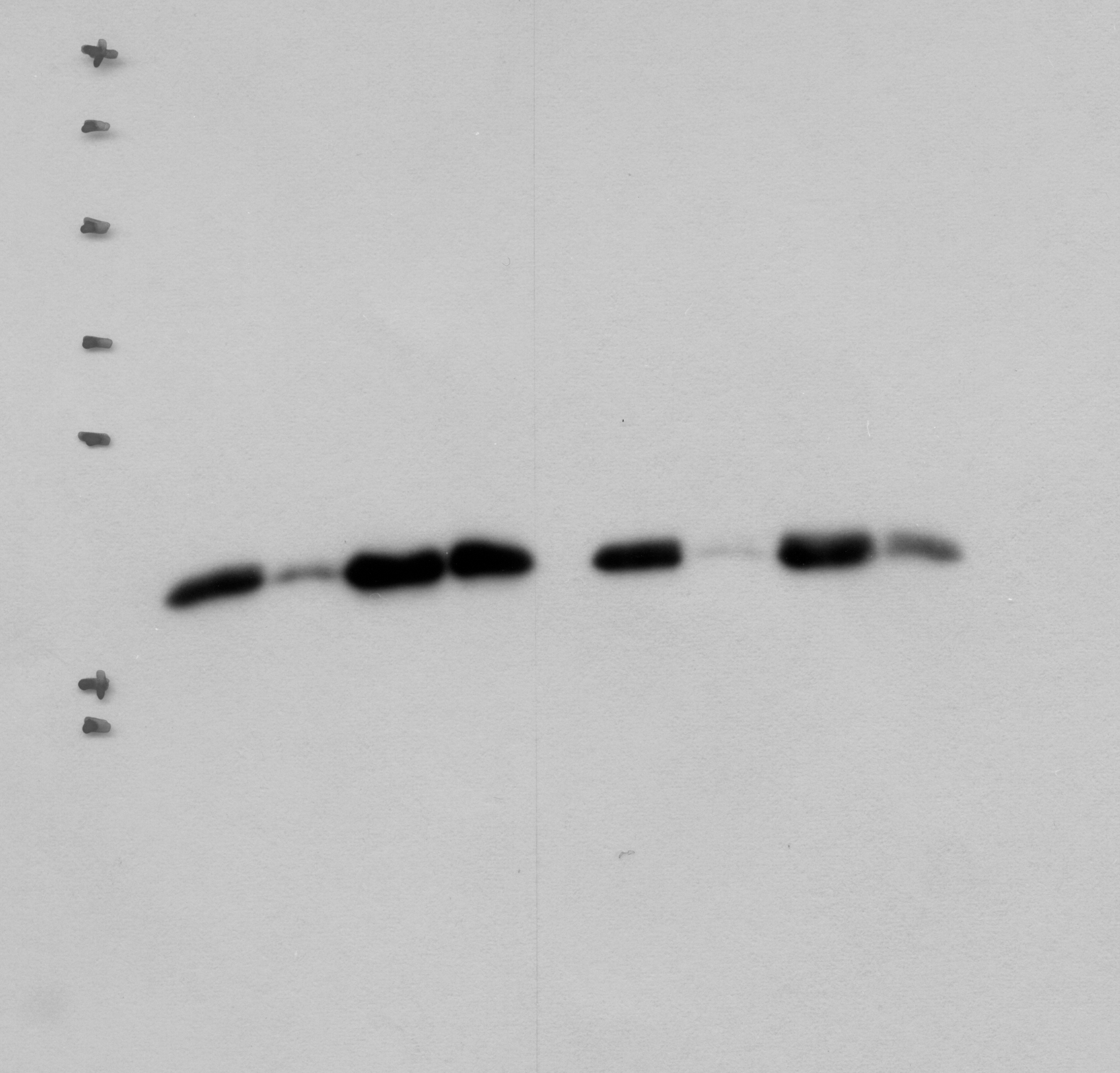

Supplement: Figure 8—source data 2. [file elife-76090-fig8-data2.zip › Figure8-source data2/Figure8C/Figure8C-PSH.tif]

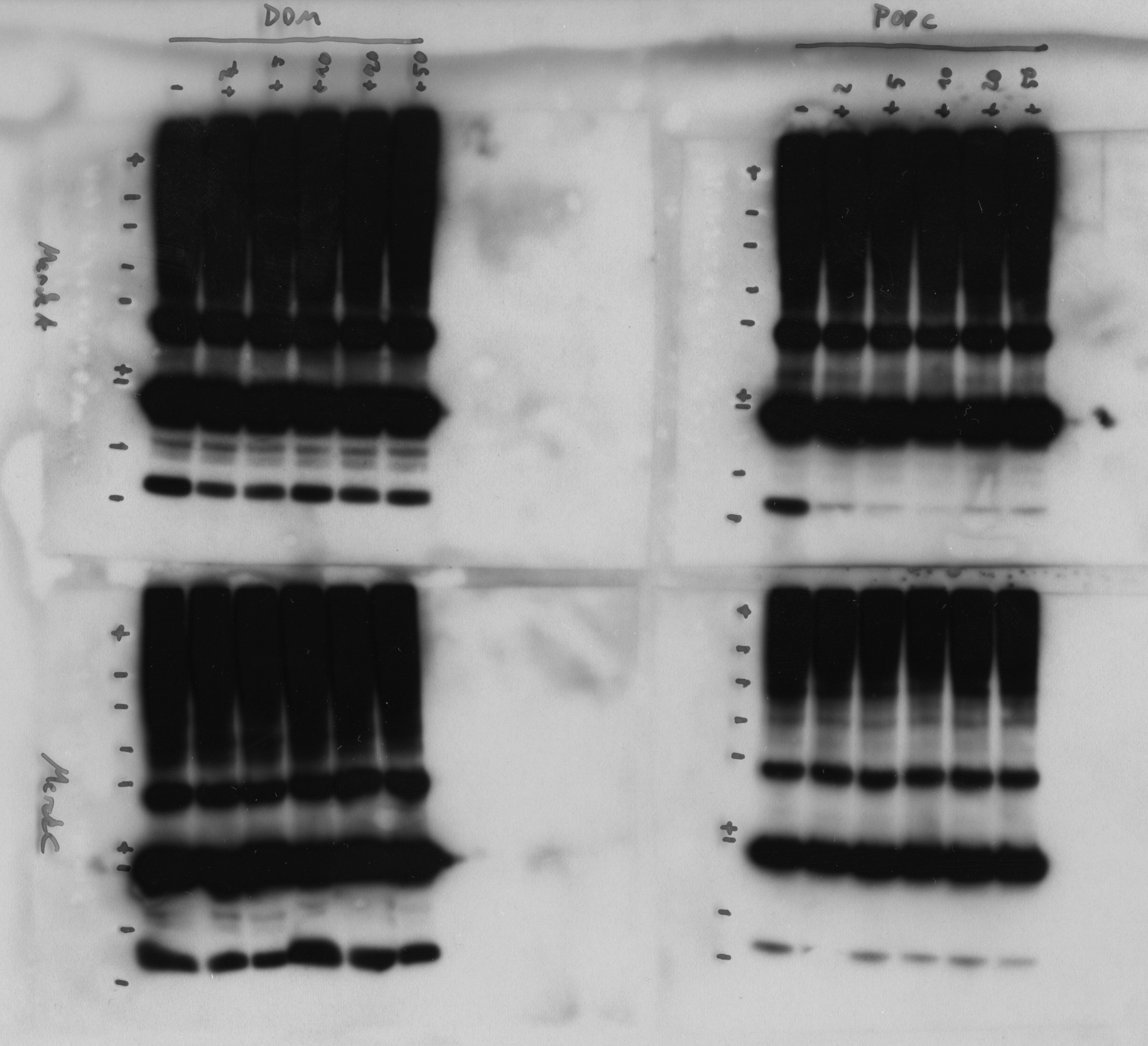

Supplement: Figure 8—source data 2. [file elife-76090-fig8-data2.zip › Figure8-source data2/Figure8E/Figure8E-Abeta-DDM-POPC.tif]

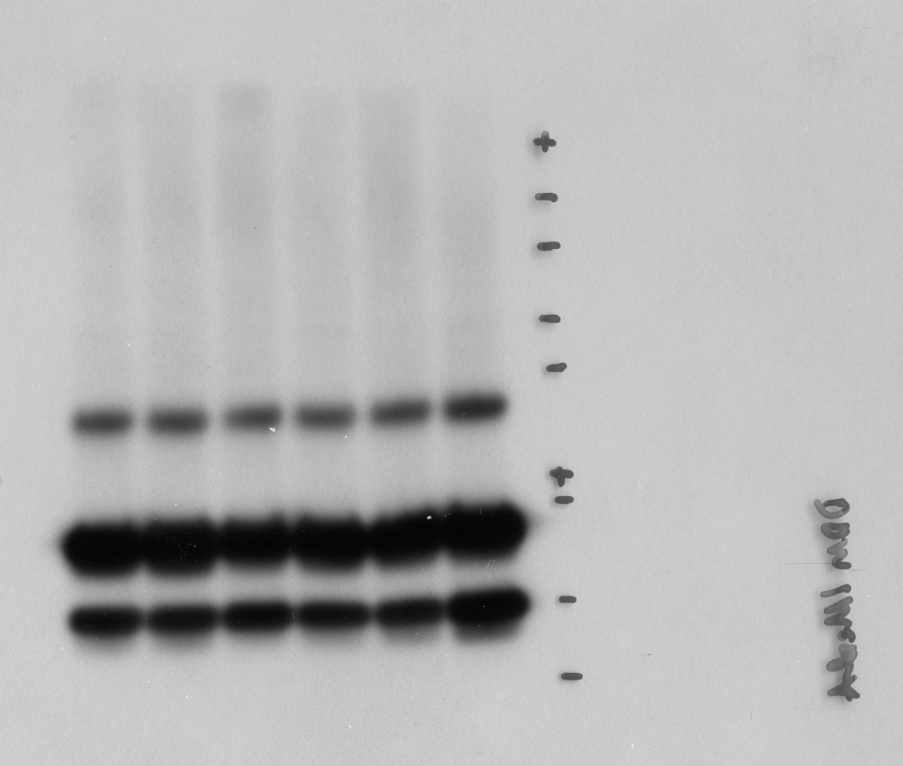

Supplement: Figure 8—source data 2. [file elife-76090-fig8-data2.zip › Figure8-source data2/Figure8E/Figure8E-AICD-DDM.tif]

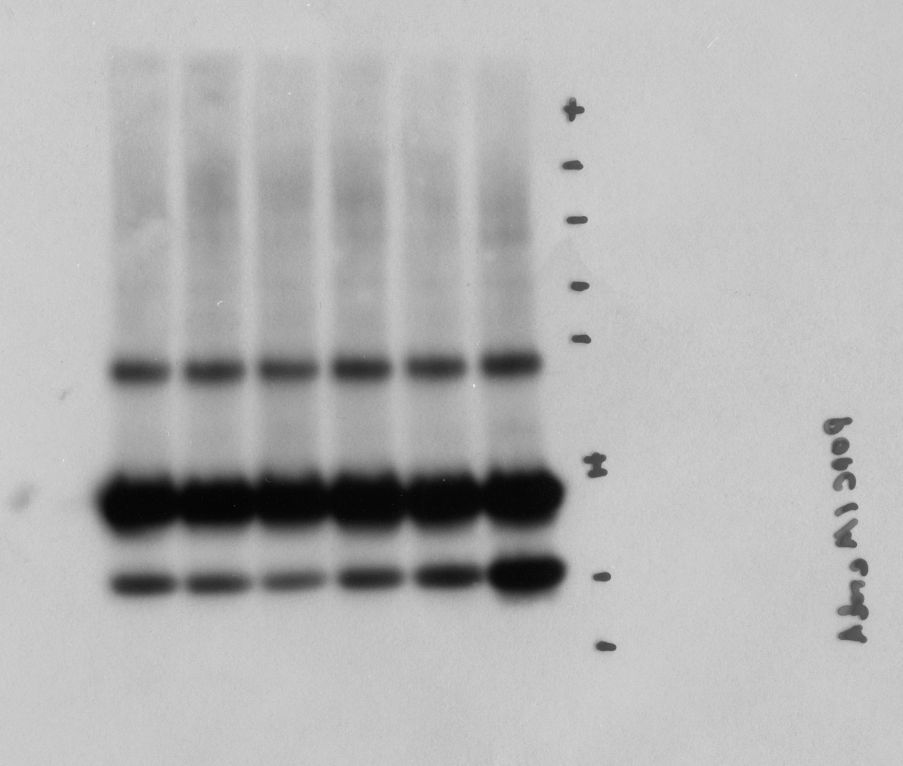

Supplement: Figure 8—source data 2. [file elife-76090-fig8-data2.zip › Figure8-source data2/Figure8E/Figure8E-AICD-POPC.tif]

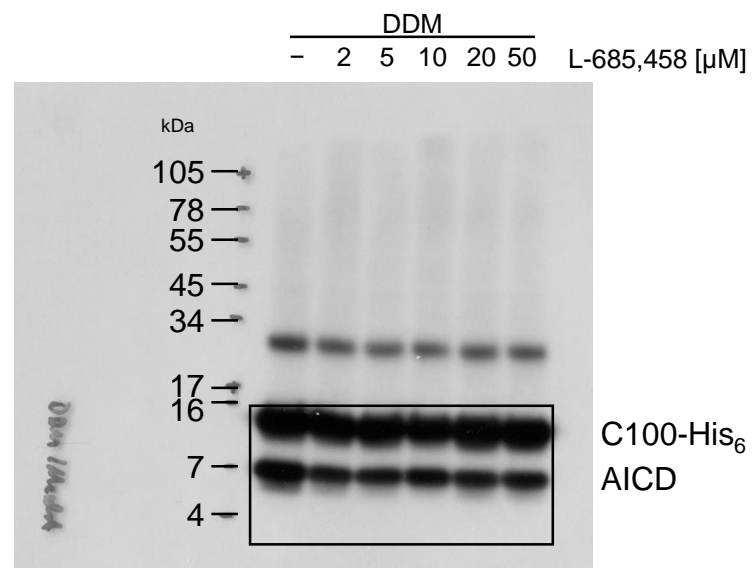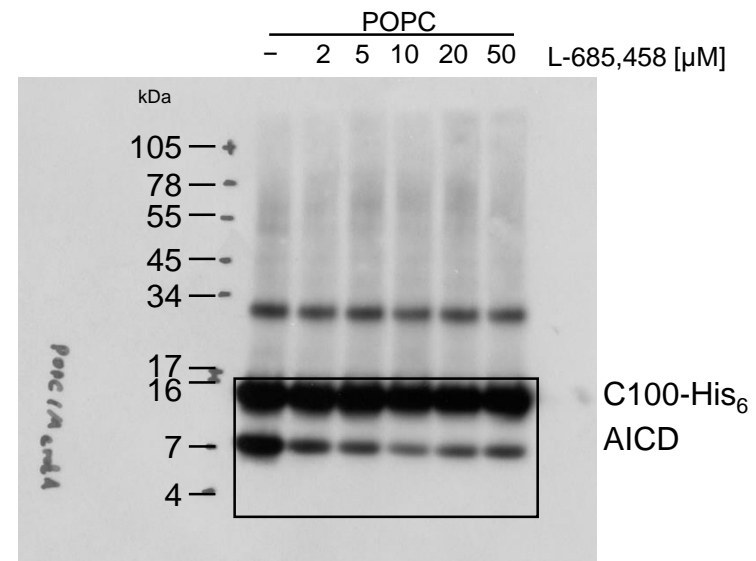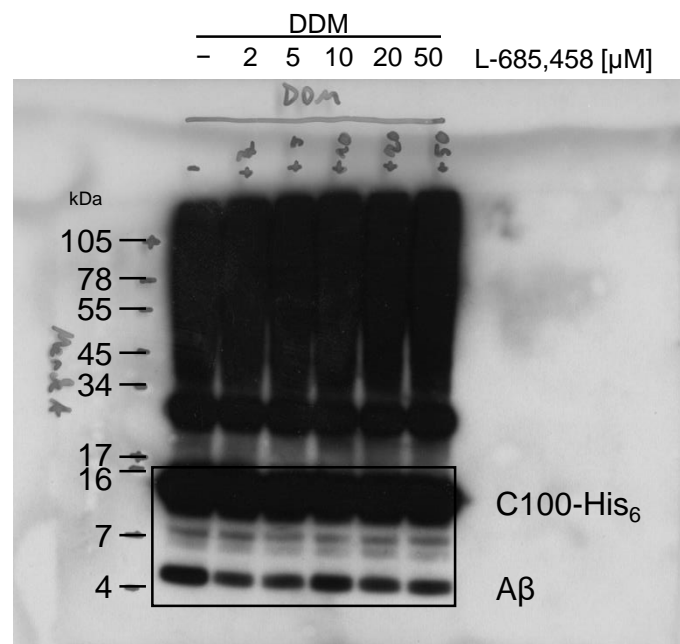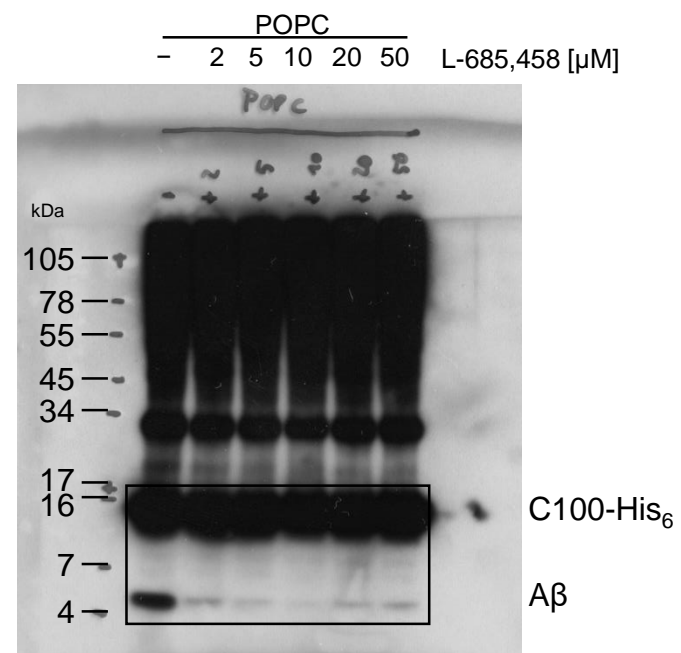

Supplement: Figure 8—source data 2. [file elife-76090-fig8-data2.zip › Figure8-source data2/Figure8E/Figure8E-annotated blots.pdf]

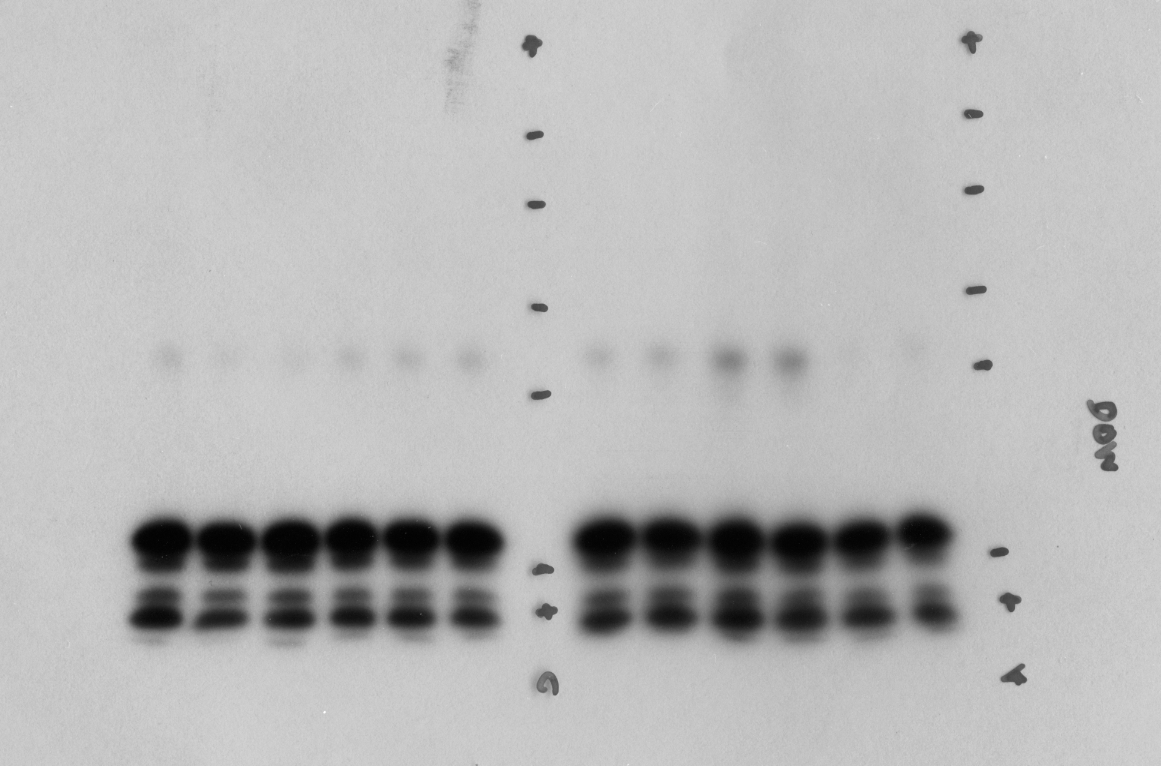

Supplement: Figure 8—source data 2. [file elife-76090-fig8-data2.zip › Figure8-source data2/Figure8E/Figure8E-PSH-DDM.tif]

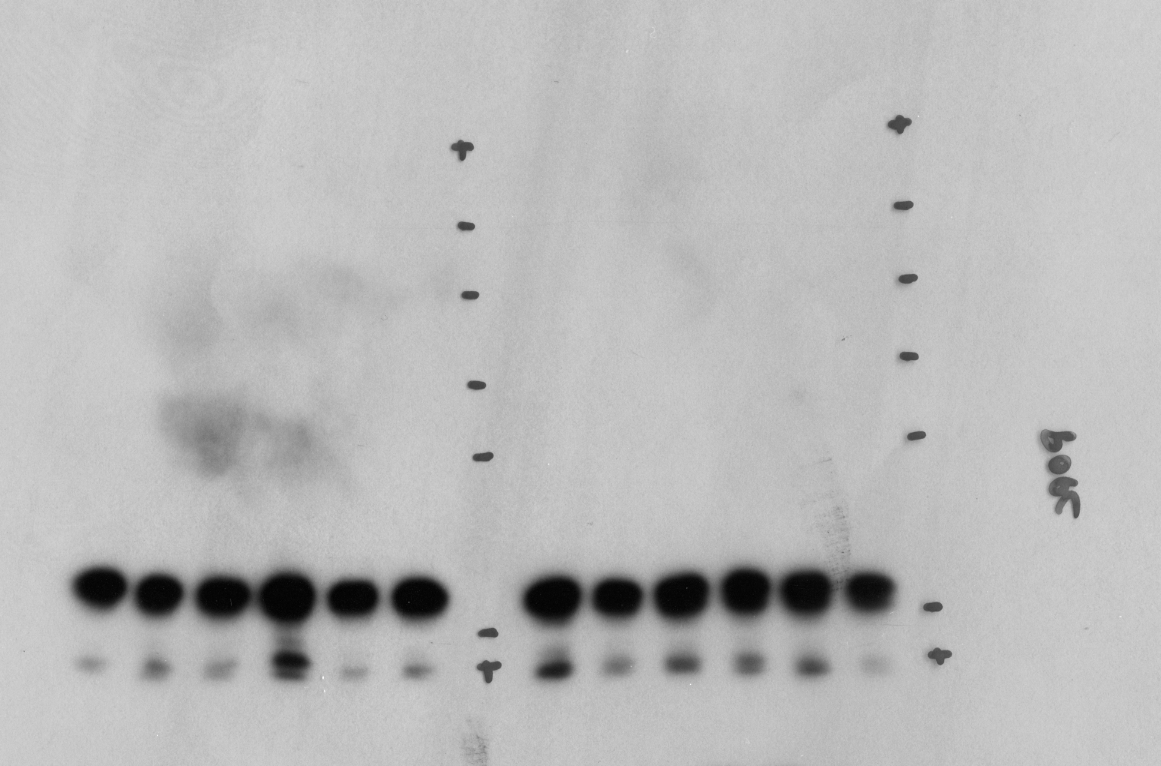

Supplement: Figure 8—source data 2. [file elife-76090-fig8-data2.zip › Figure8-source data2/Figure8E/Figure8E-PSH-POPC.tif]

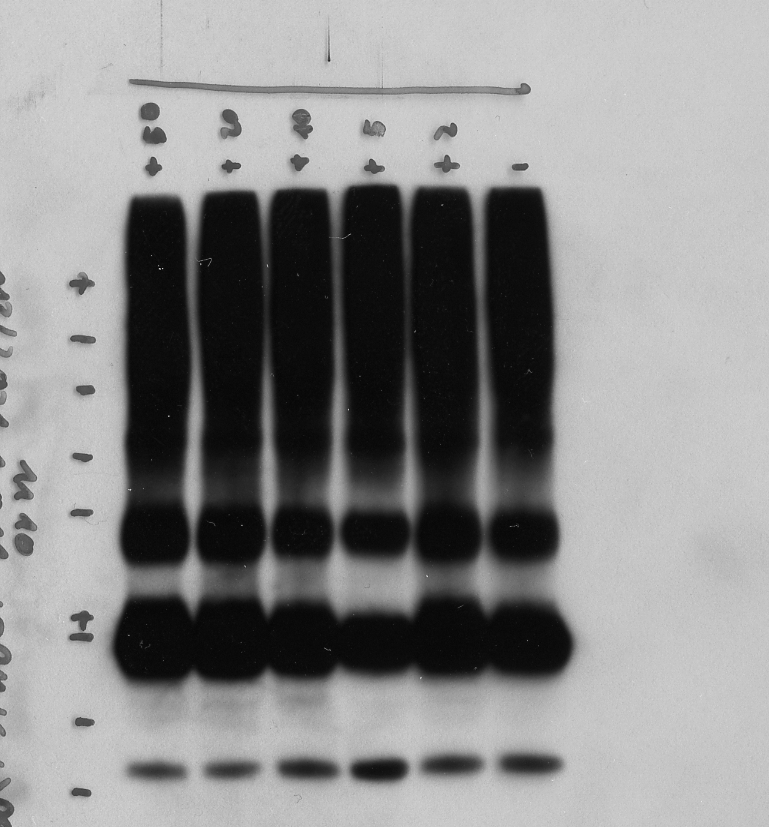

Supplement: Figure 8—source data 2. [file elife-76090-fig8-data2.zip › Figure8-source data2/Figure8F/Figure8F-Abeta-DDM.tif]

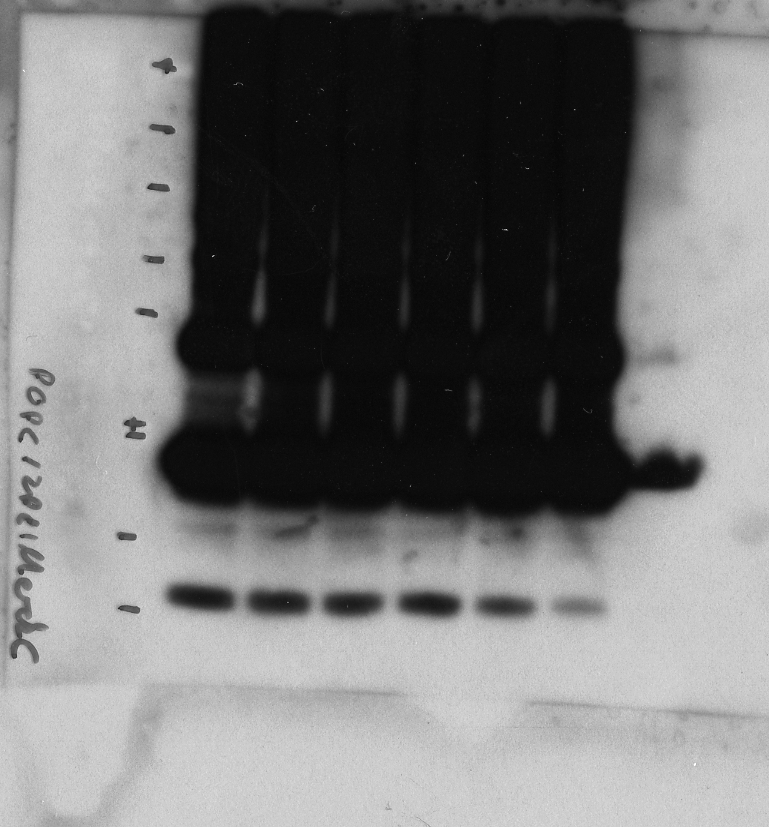

Supplement: Figure 8—source data 2. [file elife-76090-fig8-data2.zip › Figure8-source data2/Figure8F/Figure8F-Abeta-POPC.tif]

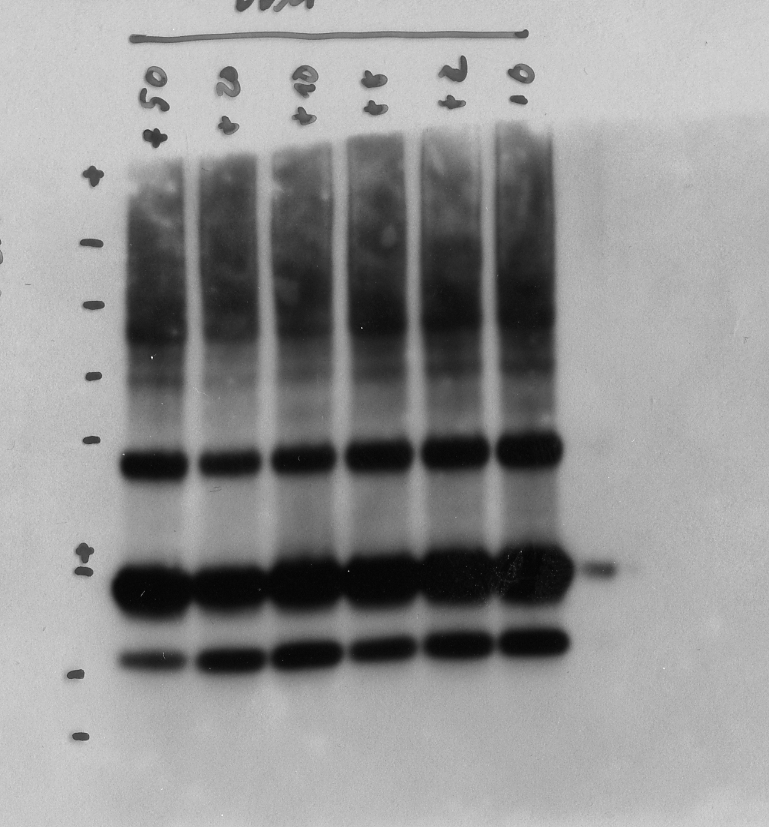

Supplement: Figure 8—source data 2. [file elife-76090-fig8-data2.zip › Figure8-source data2/Figure8F/Figure8F-AICD-DDM.tif]

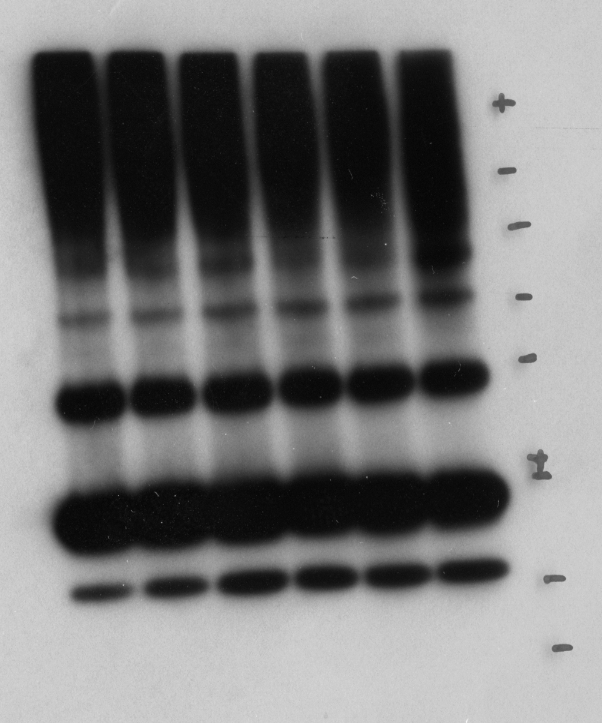

Supplement: Figure 8—source data 2. [file elife-76090-fig8-data2.zip › Figure8-source data2/Figure8F/Figure8F-AICD-POPC.tif]

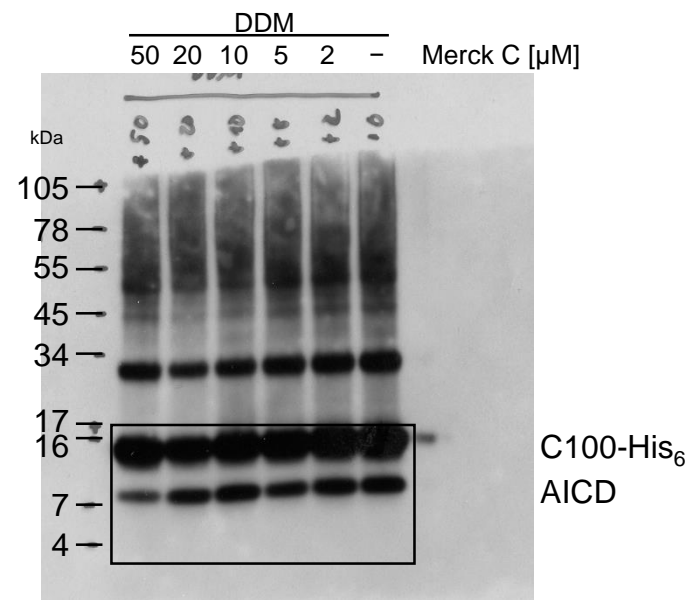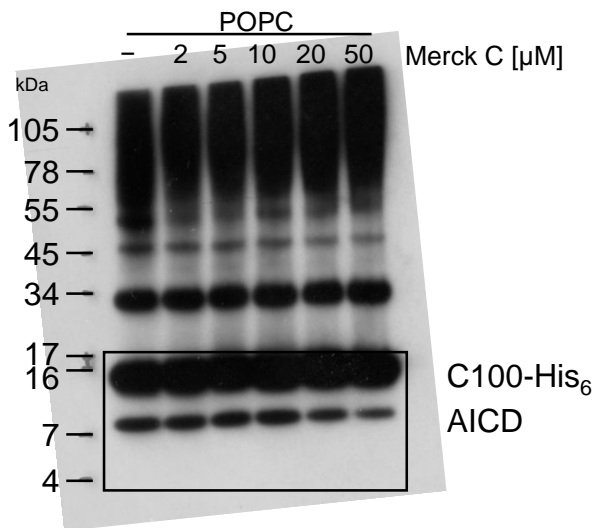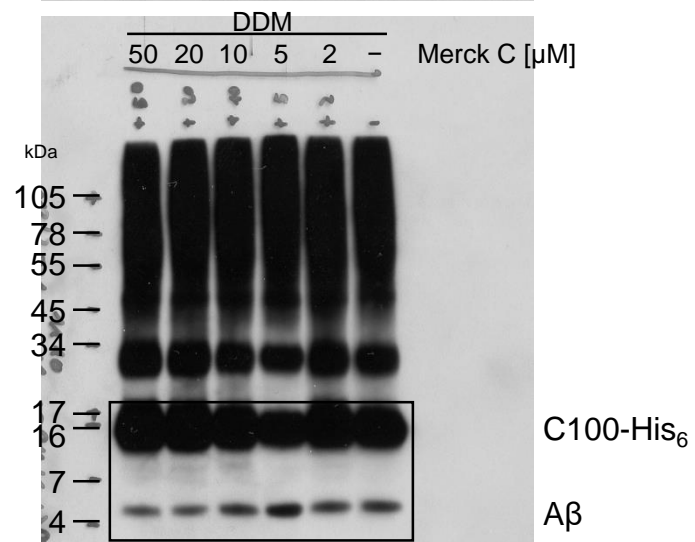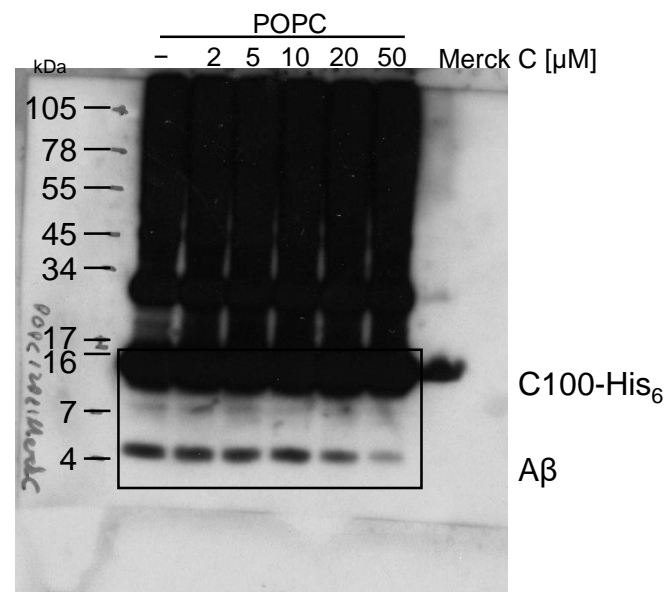

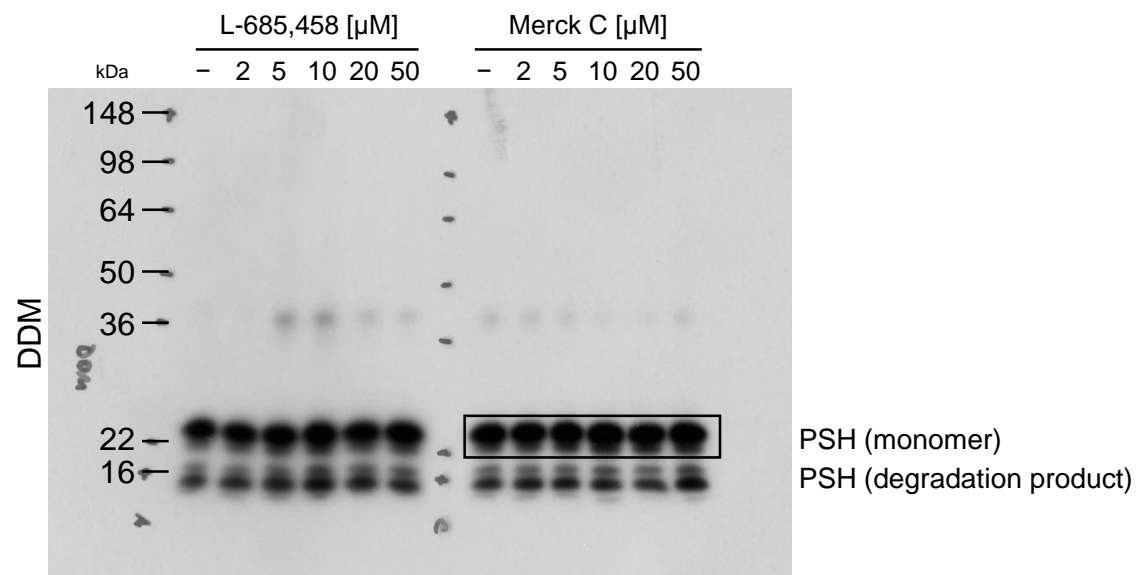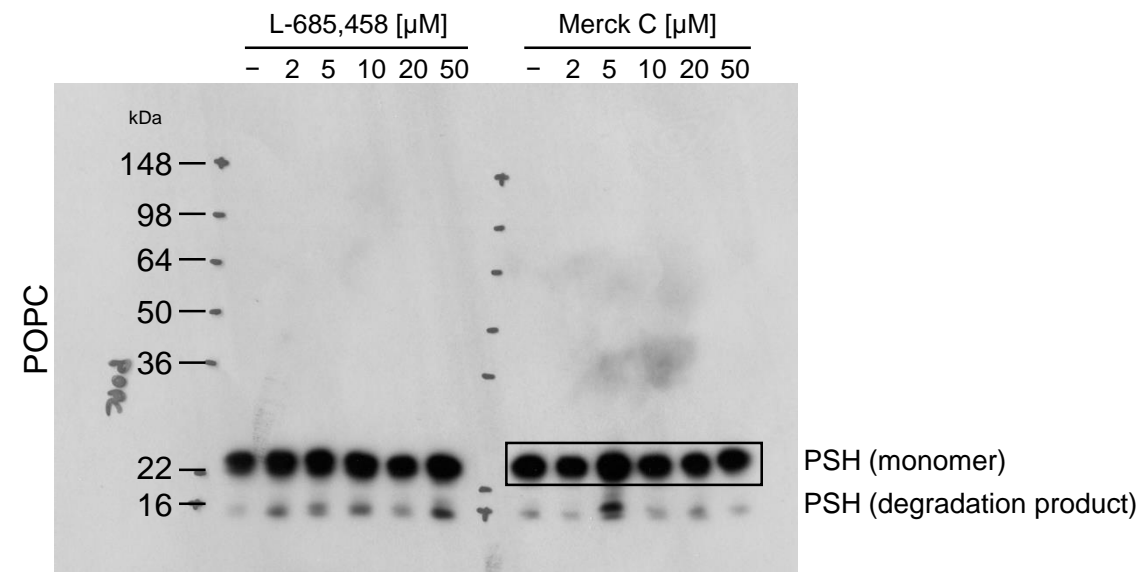

Supplement: Figure 8—source data 2. [file elife-76090-fig8-data2.zip › Figure8-source data2/Figure8F/Figure8F-annotated blots.pdf]

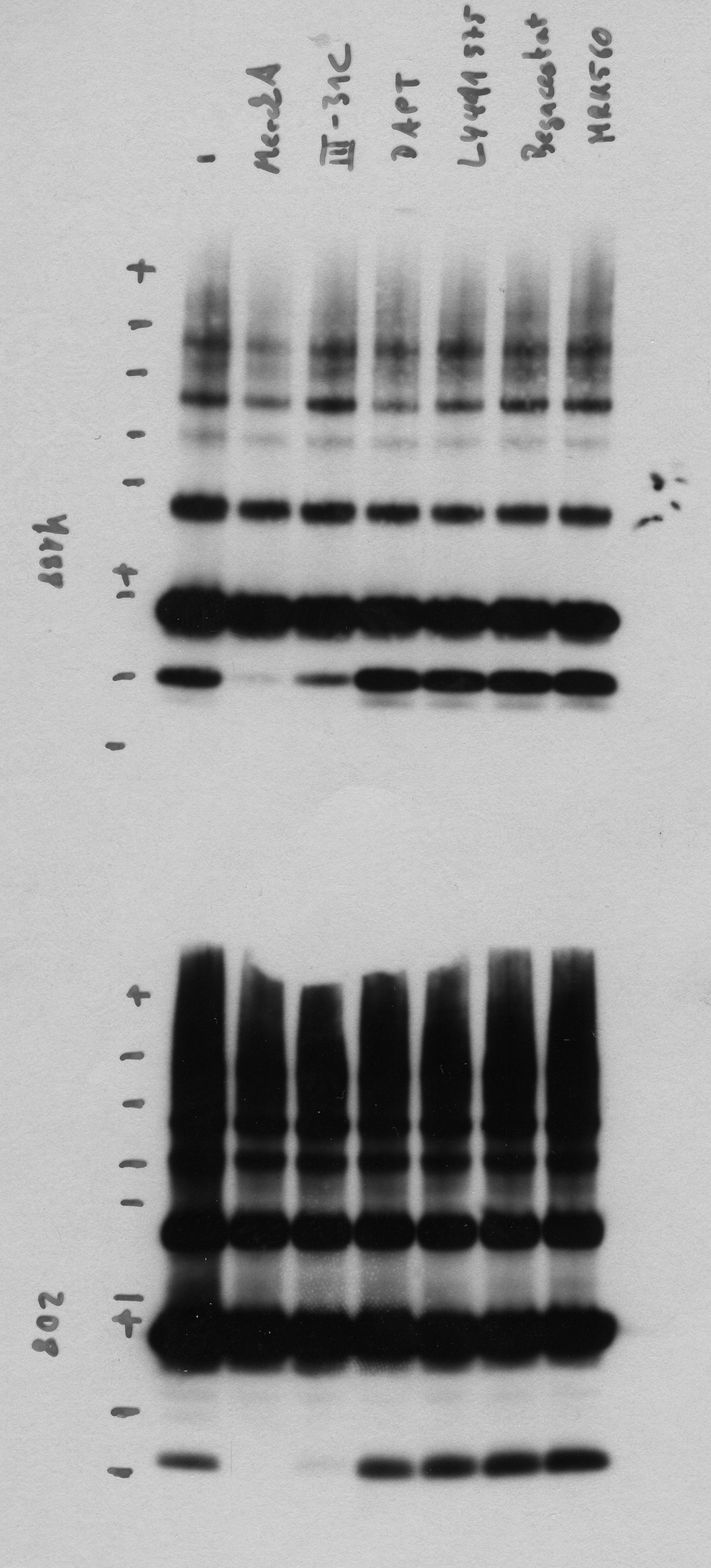

Supplement: Figure 8—source data 2. [file elife-76090-fig8-data2.zip › Figure8-source data2/Figure8G/Figure8G-Abeta-AICD.tif]

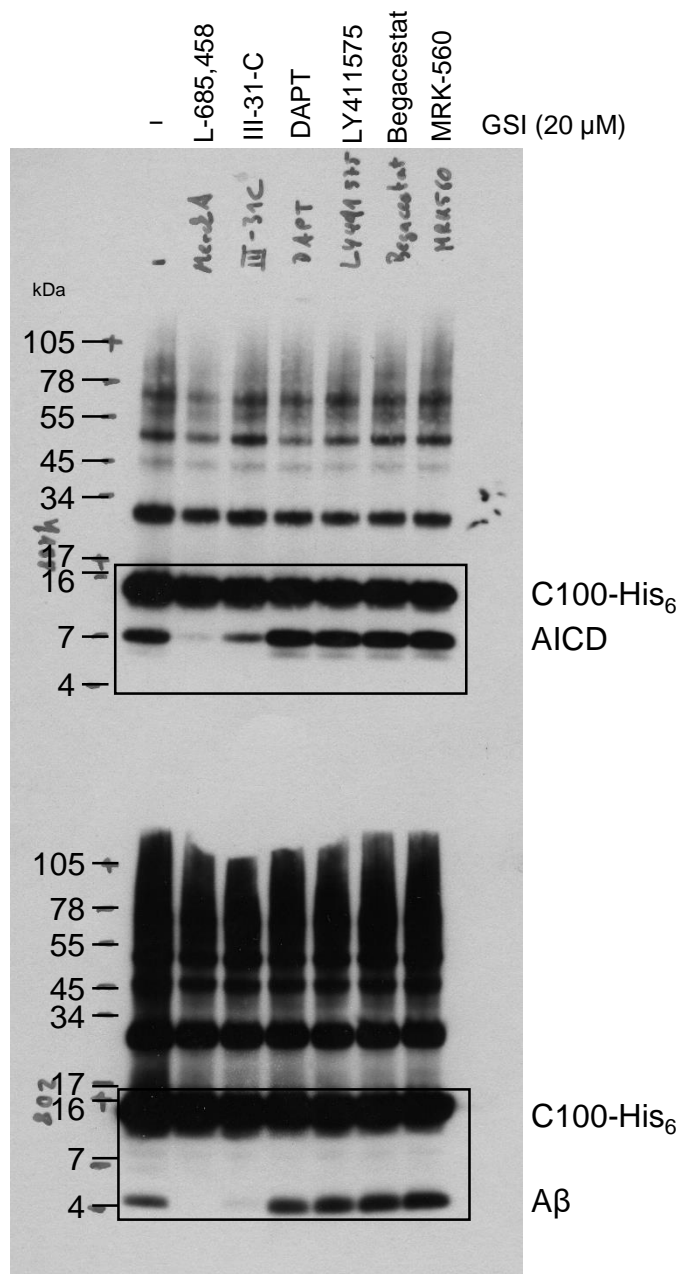

Supplement: Figure 8—source data 2. [file elife-76090-fig8-data2.zip › Figure8-source data2/Figure8G/Figure8G-annotated blots.pdf]

## Slide 1
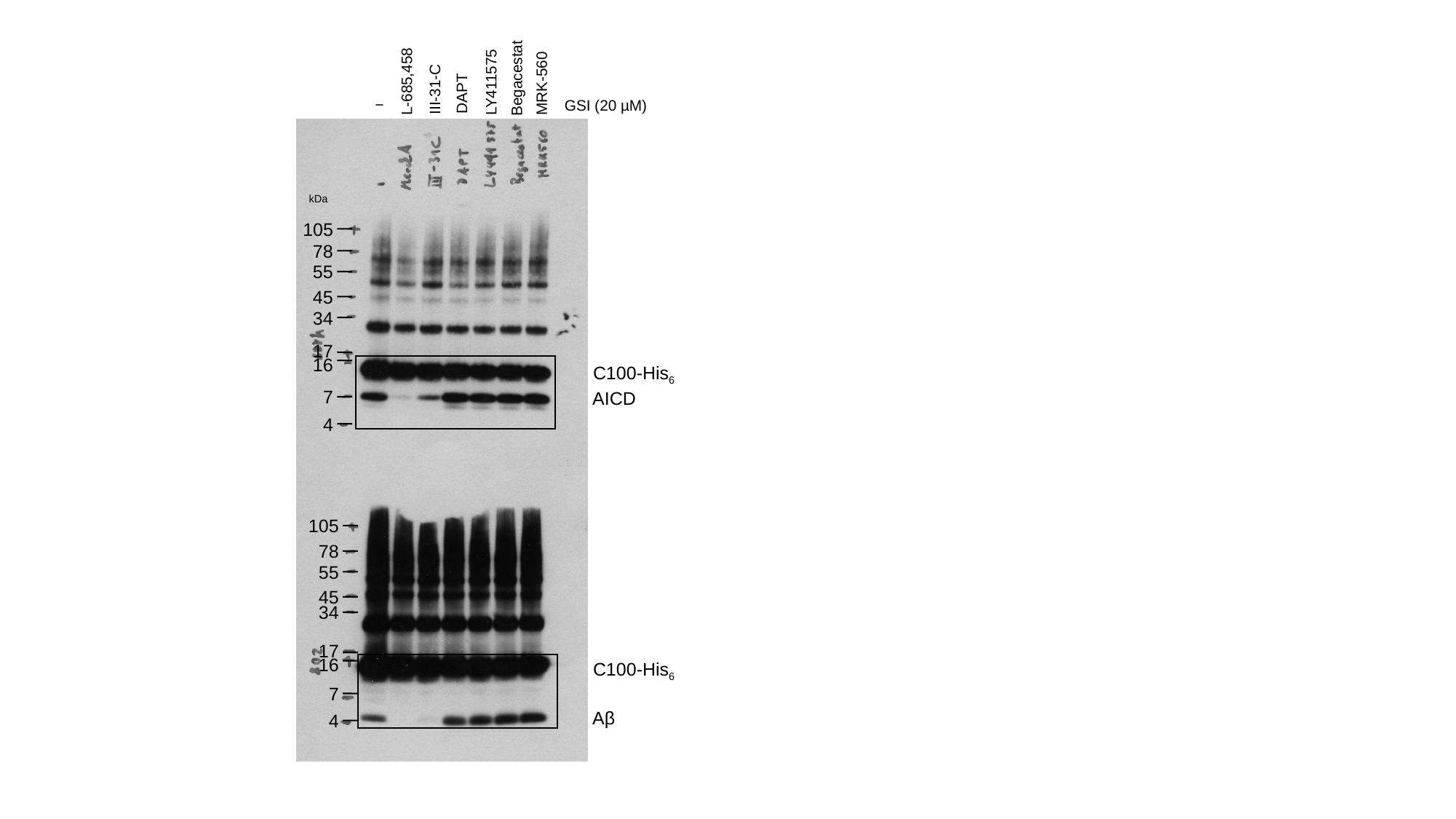

Begacestat
L-685,458
LY411575
MRK-560
III-31-C
DAPT
−
GSI (20 µM)
kDa
105
78
55
45
34
17
16
C100-His6
7
AICD
4
105
78
55
45
34
17
16
C100-His6
7
Aβ
4

Supplement: Figure 8—source data 2. [file elife-76090-fig8-data2.zip › Figure8-source data2/Figure8G/Figure8G-annotated blots.pptx]
