# Supplementary material for: Active site geometry stabilization of a presenilin homolog by the lipid bilayer promotes intramembrane proteolysis
Source: eLife. 2022 May 17;11:e76090. doi: 10.7554/eLife.76090 (PMC9282858; doi:10.7554/eLife.76090)
Supplement: Figure 2—source data 1. [file elife-76090-fig2-data1.zip › Figure2-source data1/Figure2A/Figure2A-annotated blots.pptx]

## Slide 1
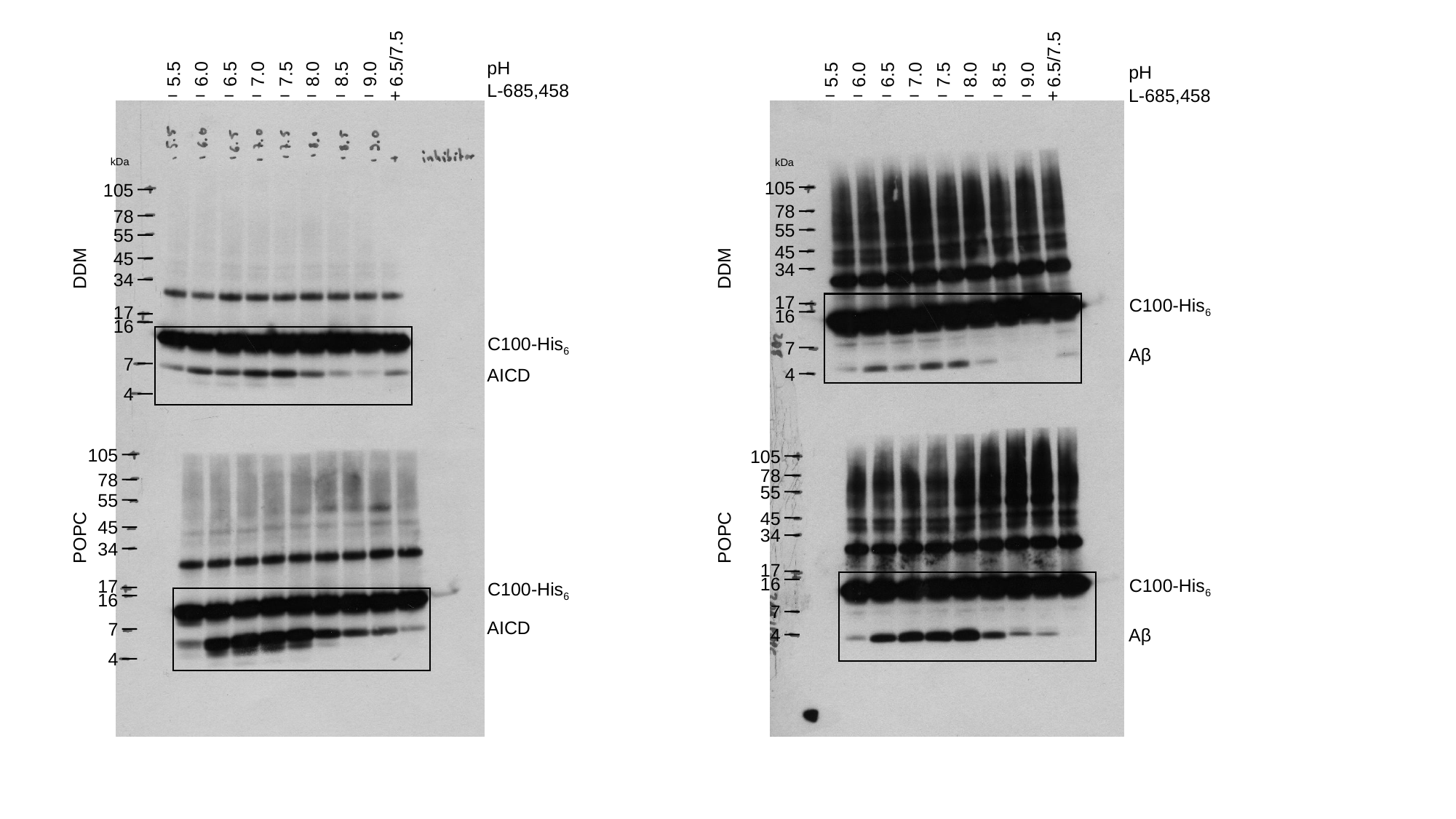

6.5/7.5
6.5/7.5
pH
pH
5.5
6.0
6.5
7.0
7.5
8.0
8.5
9.0
5.5
6.0
6.5
7.0
7.5
8.0
8.5
9.0
−
−
−
−
−
−
−
−
+
−
−
−
−
−
−
−
−
+
L-685,458
L-685,458
kDa
kDa
105
105
78
78
55
55
45
45
34
DDM
DDM
34
17
C100-His6
17
16
16
C100-His6
7
Aβ
7
4
AICD
4
105
105
78
78
55
55
45
45
34
POPC
POPC
34
17
16
C100-His6
17
C100-His6
16
7
AICD
7
Aβ
4
4

## Slide 2
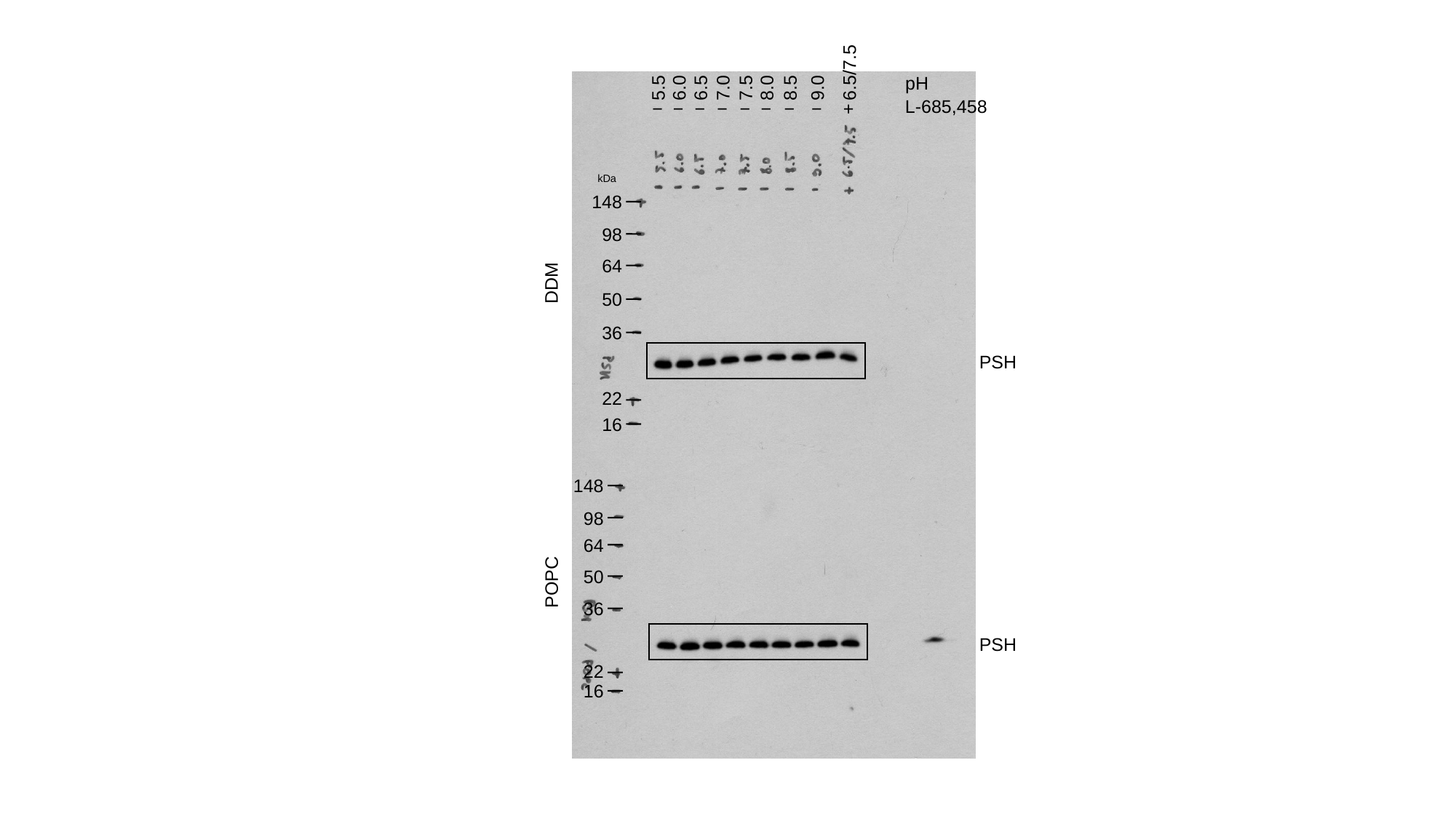

6.5/7.5
pH
5.5
6.0
6.5
7.0
7.5
8.0
8.5
9.0
−
−
−
−
−
−
−
−
+
L-685,458
kDa
148
98
64
DDM
50
36
PSH
22
16
148
98
64
50
POPC
36
PSH
22
16
