# Supplementary material for: Active site geometry stabilization of a presenilin homolog by the lipid bilayer promotes intramembrane proteolysis
Source: eLife. 2022 May 17;11:e76090. doi: 10.7554/eLife.76090 (PMC9282858; doi:10.7554/eLife.76090)
Supplement: Figure 3—source data 1. [file elife-76090-fig3-data1.zip › Figure3-source data1/Figure3A-annotated blots.pptx]

## Slide 1
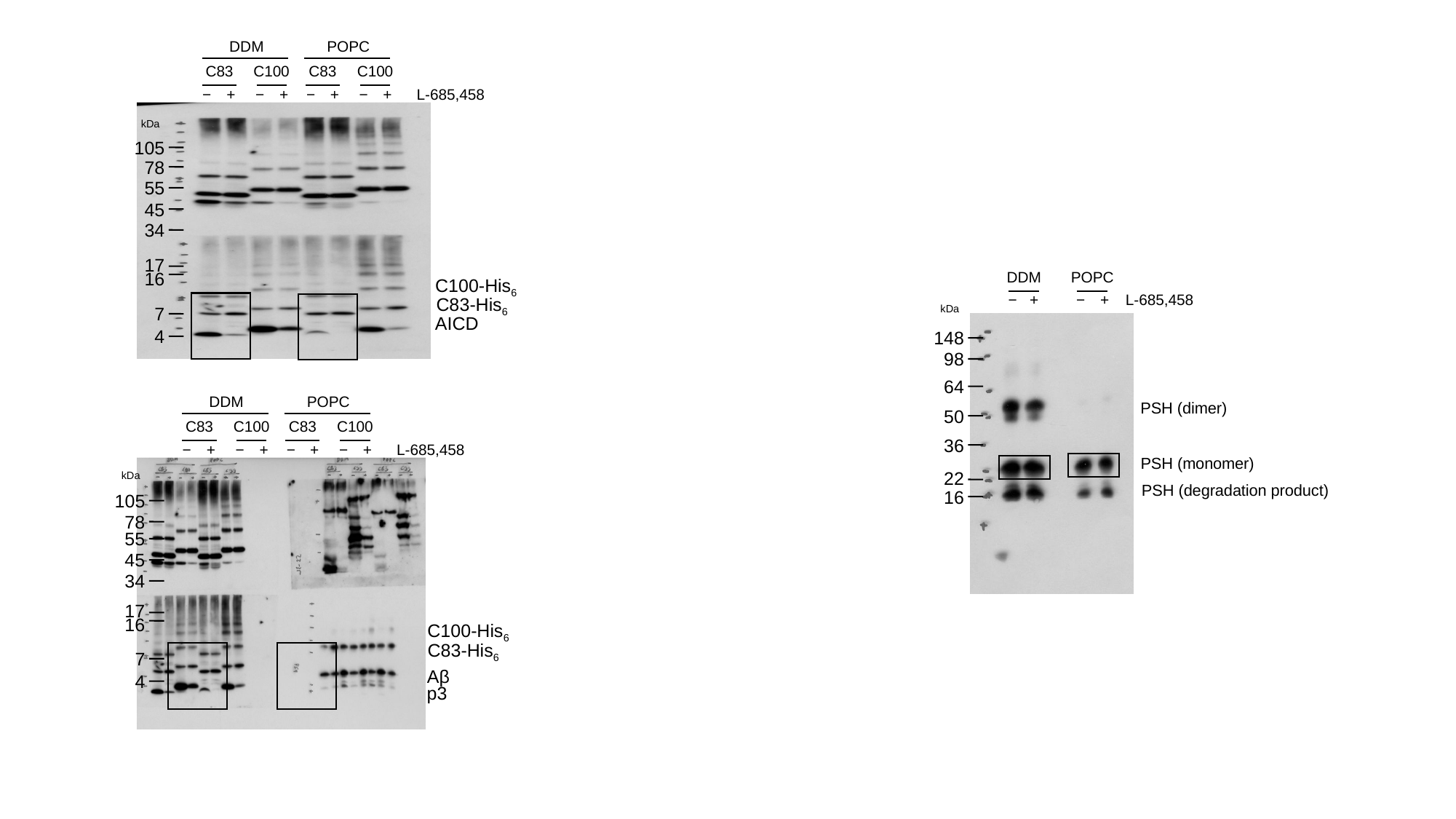

DDM
POPC
C83
C100
C83
C100
−
+
−
+
−
+
−
+
L-685,458
kDa
105
78
55
45
34
17
16
DDM
POPC
C100-His6
−
+
−
+
L-685,458
C83-His6
kDa
7
AICD
4
148
98
64
DDM
POPC
C83
C100
C83
C100
−
+
−
+
−
+
−
+
L-685,458
PSH (dimer)
50
36
PSH (monomer)
kDa
22
PSH (degradation product)
16
105
78
55
45
34
17
16
C100-His6
C83-His6
7
Aβ
4
p3
