# Supplementary material for: Active site geometry stabilization of a presenilin homolog by the lipid bilayer promotes intramembrane proteolysis
Source: eLife. 2022 May 17;11:e76090. doi: 10.7554/eLife.76090 (PMC9282858; doi:10.7554/eLife.76090)
Supplement: Figure 4—source data 1. [file elife-76090-fig4-data1.zip › Figure4-source data1/Figure4E-annotated blots.pptx]

## Slide 1
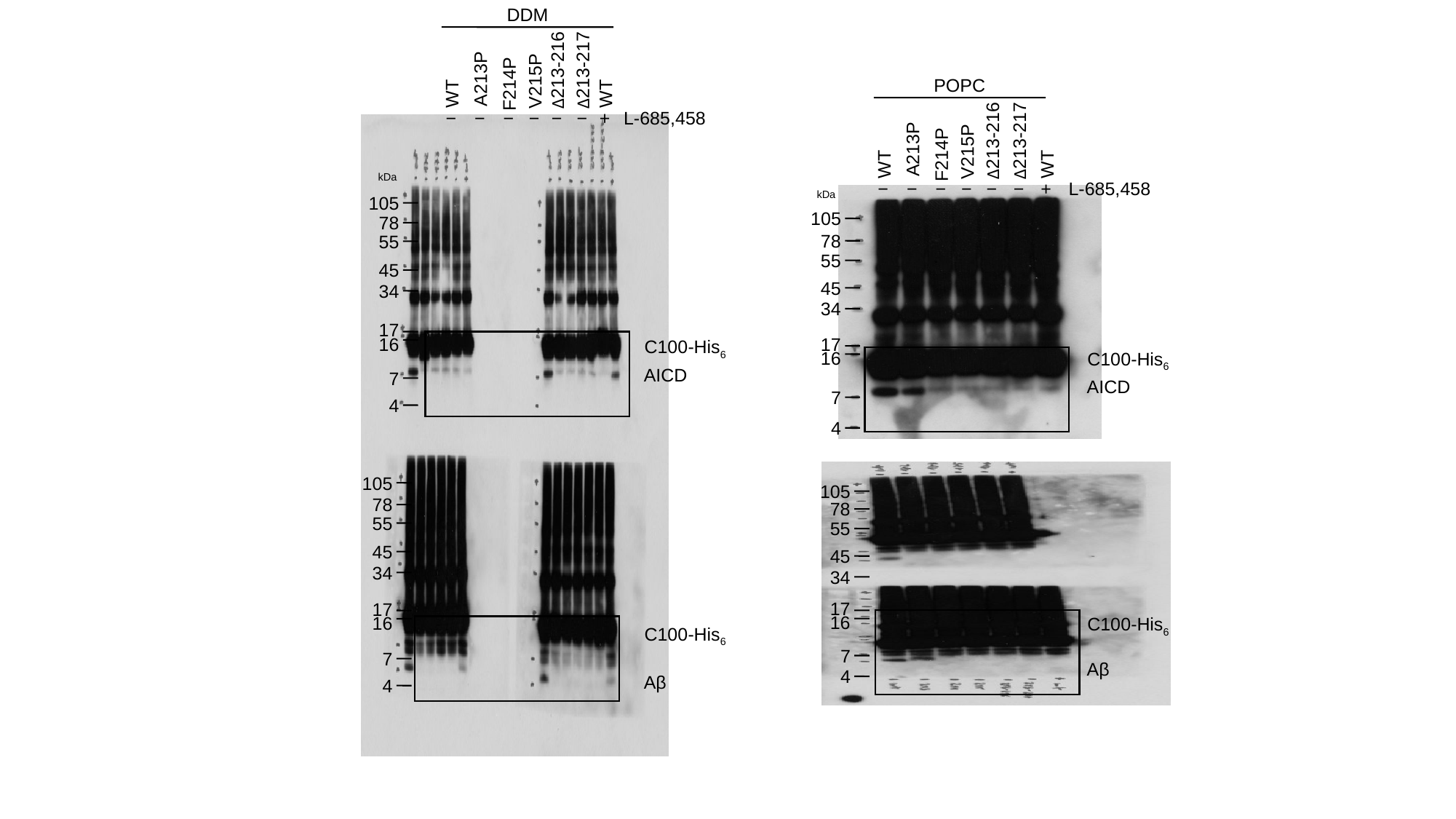

DDM
Δ213-216
Δ213-217
A213P
V215P
F214P
POPC
WT
WT
−
−
−
−
−
−
+
L-685,458
Δ213-216
Δ213-217
A213P
V215P
F214P
WT
WT
kDa
−
−
−
−
−
−
+
L-685,458
kDa
105
105
78
78
55
55
45
45
34
34
17
16
17
C100-His6
16
C100-His6
AICD
7
AICD
7
4
4
105
105
78
78
55
55
45
45
34
34
17
17
16
16
C100-His6
C100-His6
7
7
Aβ
4
Aβ
4

## Slide 2
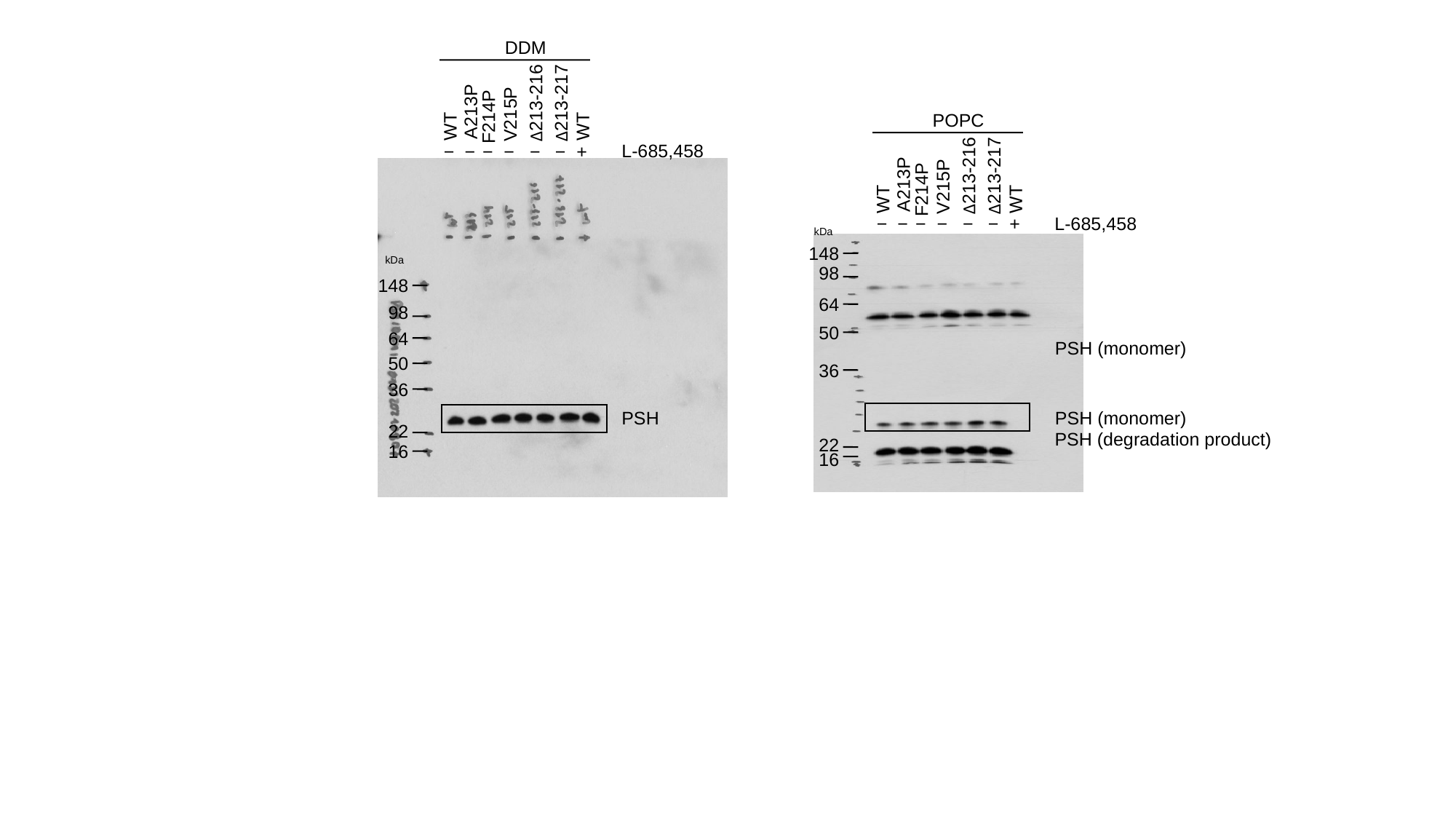

DDM
Δ213-216
Δ213-217
A213P
V215P
F214P
POPC
WT
WT
−
−
−
−
−
−
+
L-685,458
Δ213-216
Δ213-217
A213P
V215P
F214P
WT
WT
−
−
−
−
−
−
+
L-685,458
kDa
148
kDa
98
148
64
98
50
64
PSH (monomer)
50
36
36
PSH
PSH (monomer)
22
PSH (degradation product)
22
16
16
