# Supplementary material for: Active site geometry stabilization of a presenilin homolog by the lipid bilayer promotes intramembrane proteolysis
Source: eLife. 2022 May 17;11:e76090. doi: 10.7554/eLife.76090 (PMC9282858; doi:10.7554/eLife.76090)
Supplement: Figure 7—source data 2. [file elife-76090-fig7-data2.zip › Figure7-source data2/Figure7F-annotated blots.pptx]

## Slide 1
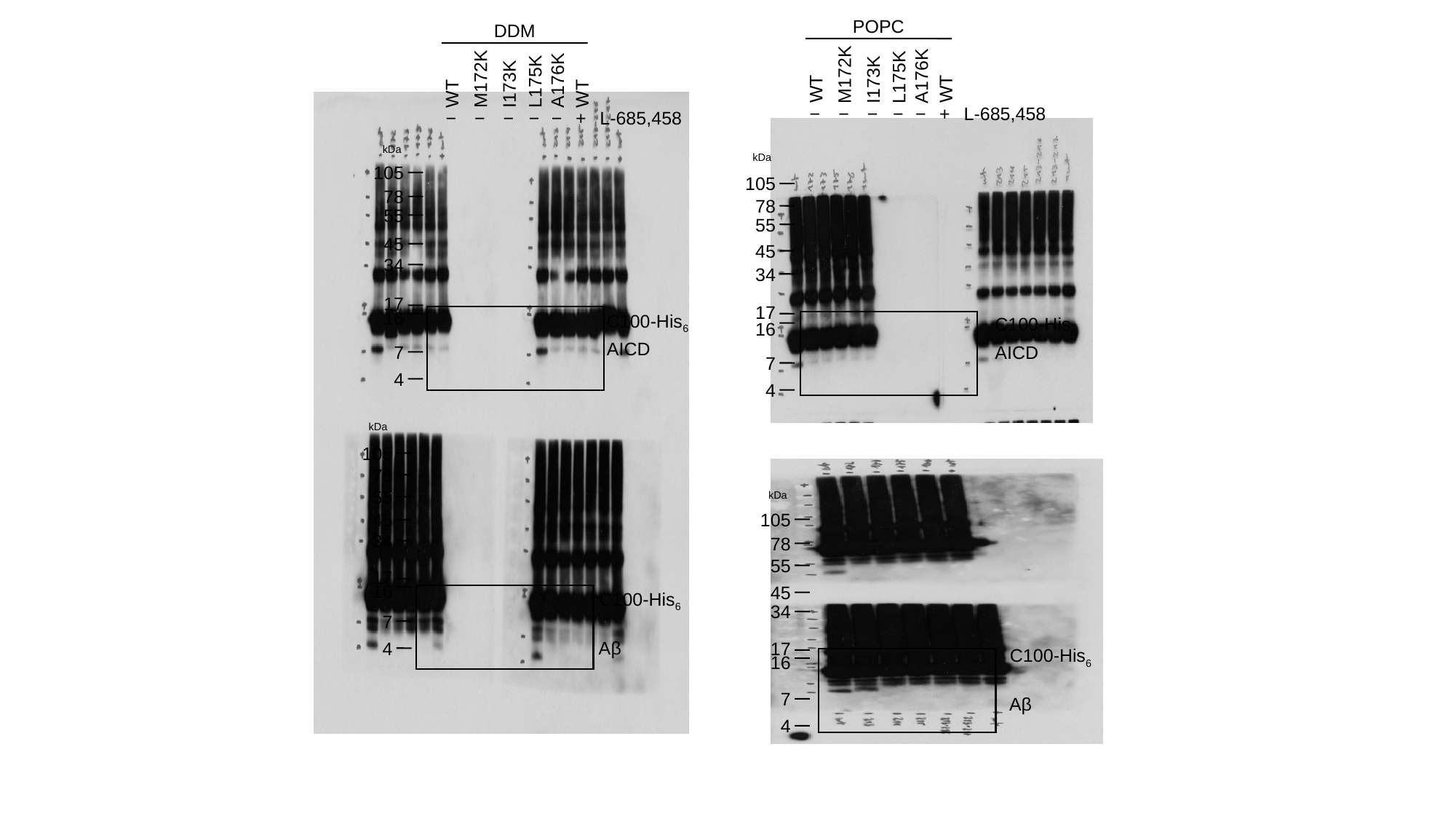

POPC
DDM
M172K
A176K
L175K
M172K
I173K
A176K
L175K
I173K
WT
WT
WT
WT
−
−
−
−
−
+
−
−
−
−
−
+
L-685,458
L-685,458
kDa
kDa
105
105
78
78
55
55
45
45
34
34
17
17
16
C100-His6
C100-His6
16
AICD
7
AICD
7
4
4
kDa
105
78
kDa
55
105
45
34
78
55
17
16
45
C100-His6
34
7
Aβ
4
17
C100-His6
16
7
Aβ
4

## Slide 2
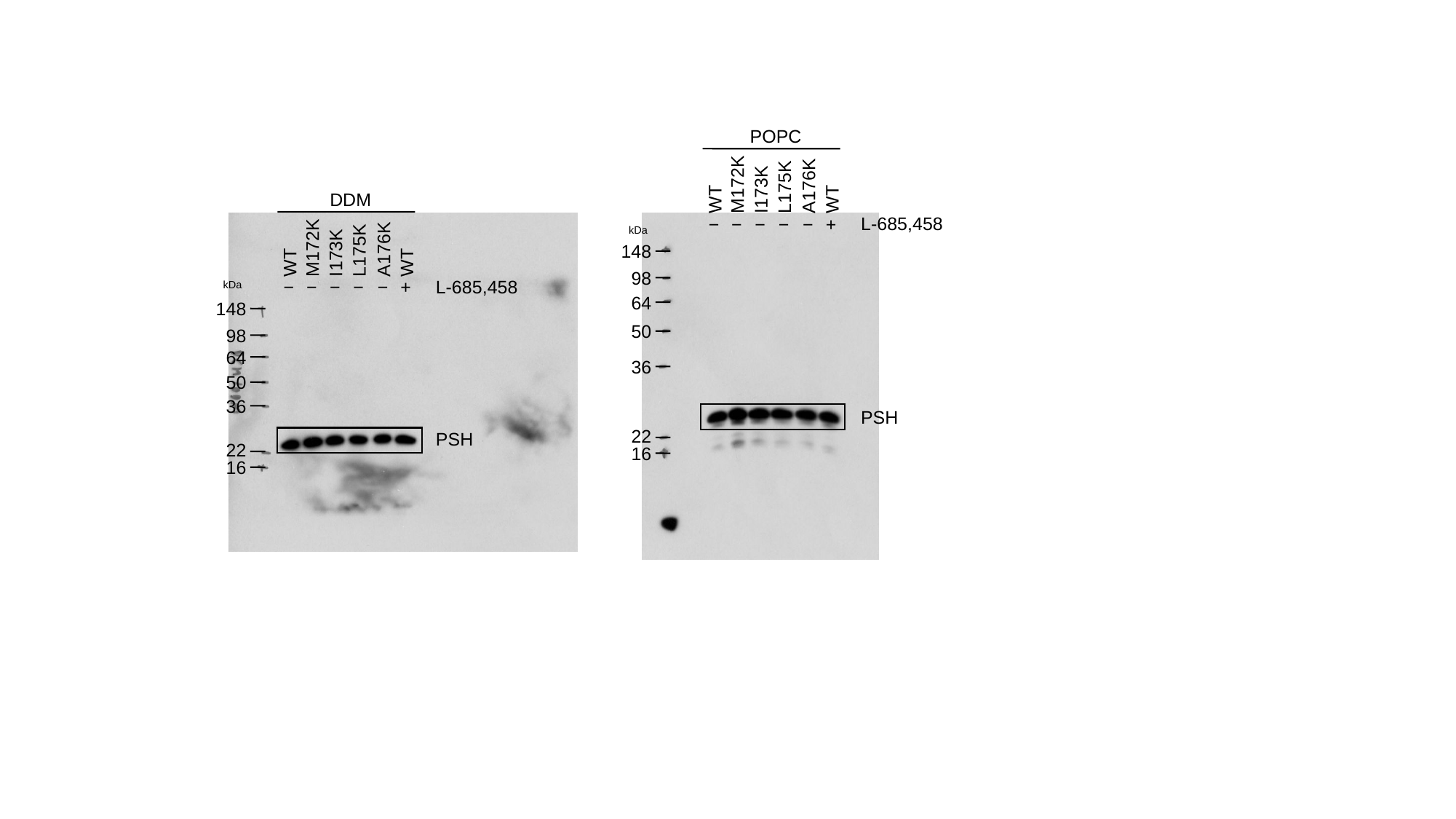

POPC
M172K
A176K
L175K
I173K
WT
WT
DDM
−
−
−
−
−
+
L-685,458
kDa
M172K
A176K
L175K
148
I173K
WT
WT
98
−
−
−
−
−
+
kDa
L-685,458
64
148
50
98
64
36
50
36
PSH
22
PSH
22
16
16
