# Supplementary material for: Active site geometry stabilization of a presenilin homolog by the lipid bilayer promotes intramembrane proteolysis
Source: eLife. 2022 May 17;11:e76090. doi: 10.7554/eLife.76090 (PMC9282858; doi:10.7554/eLife.76090)
Supplement: Figure 8—source data 2. [file elife-76090-fig8-data2.zip › Figure8-source data2/Figure8E/Figure8E-annotated blots.pptx]

## Slide 1
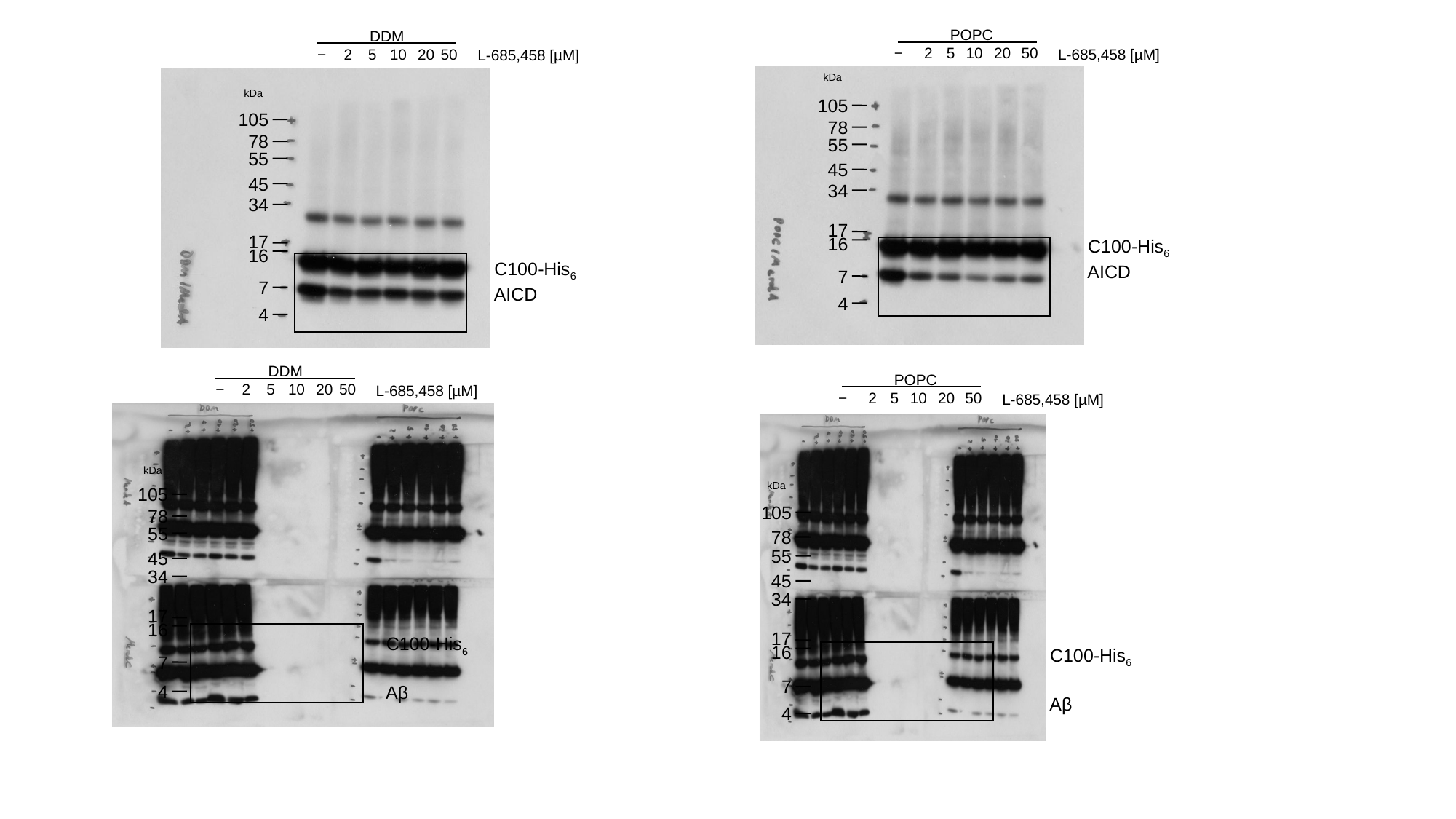

POPC
−
2
5
10
20
50
L-685,458 [µM]
105
78
55
45
34
17
16
C100-His6
AICD
7
4
DDM
−
2
5
10
20
50
L-685,458 [µM]
kDa
kDa
105
78
55
45
34
17
16
C100-His6
7
AICD
4
DDM
−
2
5
10
20
50
L-685,458 [µM]
105
78
55
45
34
17
16
C100-His6
7
4
Aβ
POPC
−
2
5
10
20
50
L-685,458 [µM]
105
78
55
45
34
17
16
C100-His6
7
Aβ
4
kDa
kDa

## Slide 2
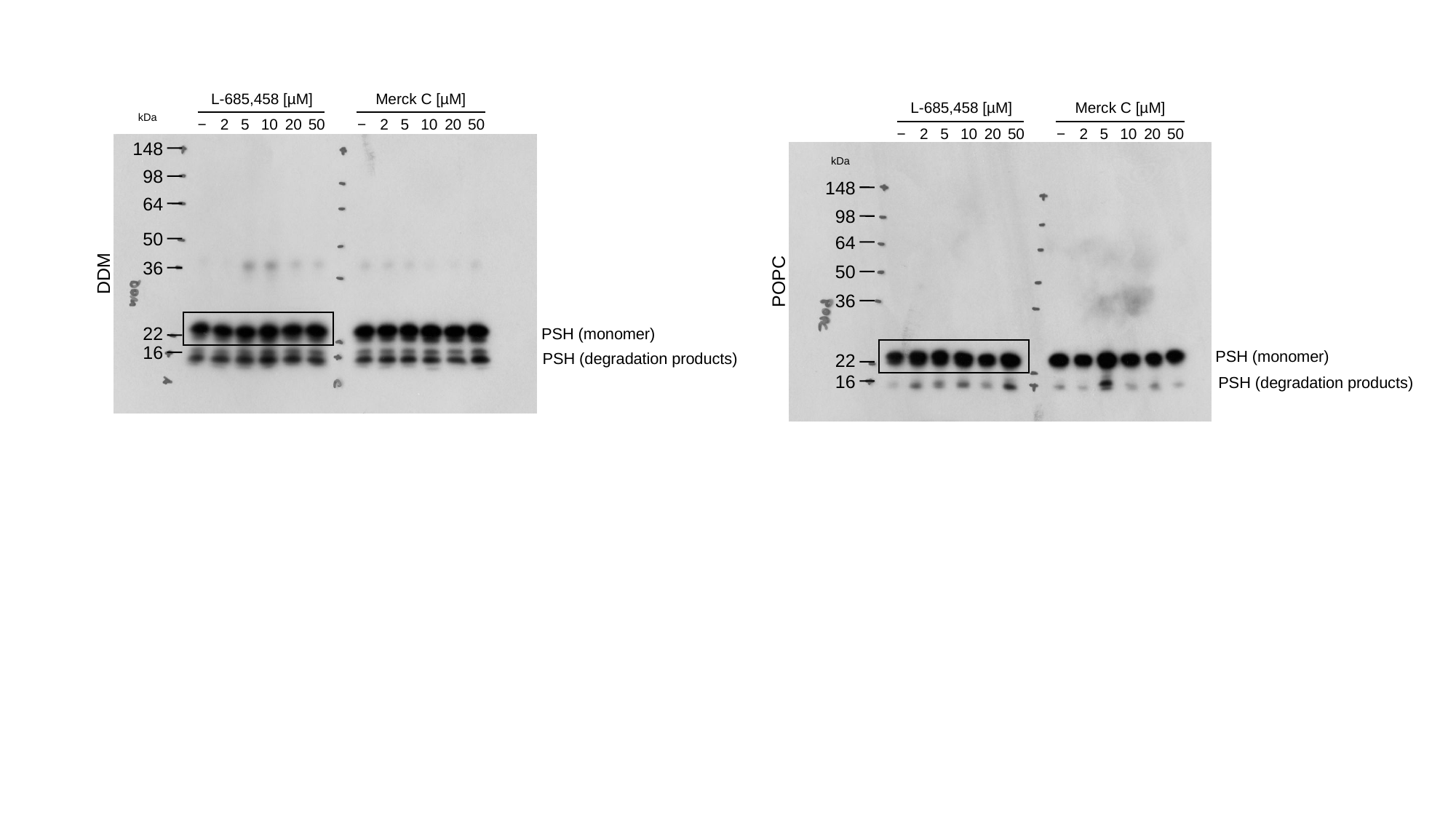

L-685,458 [µM]
Merck C [µM]
L-685,458 [µM]
Merck C [µM]
−
2
5
10
20
50
−
2
5
10
20
50
148
98
64
50
POPC
36
PSH (monomer)
22
16
kDa
−
2
5
10
20
50
−
2
5
10
20
50
148
kDa
98
64
50
36
DDM
22
PSH (monomer)
16
PSH (degradation products)
PSH (degradation products)
