# Supplementary material for: Active site geometry stabilization of a presenilin homolog by the lipid bilayer promotes intramembrane proteolysis
Source: eLife. 2022 May 17;11:e76090. doi: 10.7554/eLife.76090 (PMC9282858; doi:10.7554/eLife.76090)
Supplement: Figure 8—source data 2. [file elife-76090-fig8-data2.zip › Figure8-source data2/Figure8F/Figure8F-annotated blots.pptx]

## Slide 1
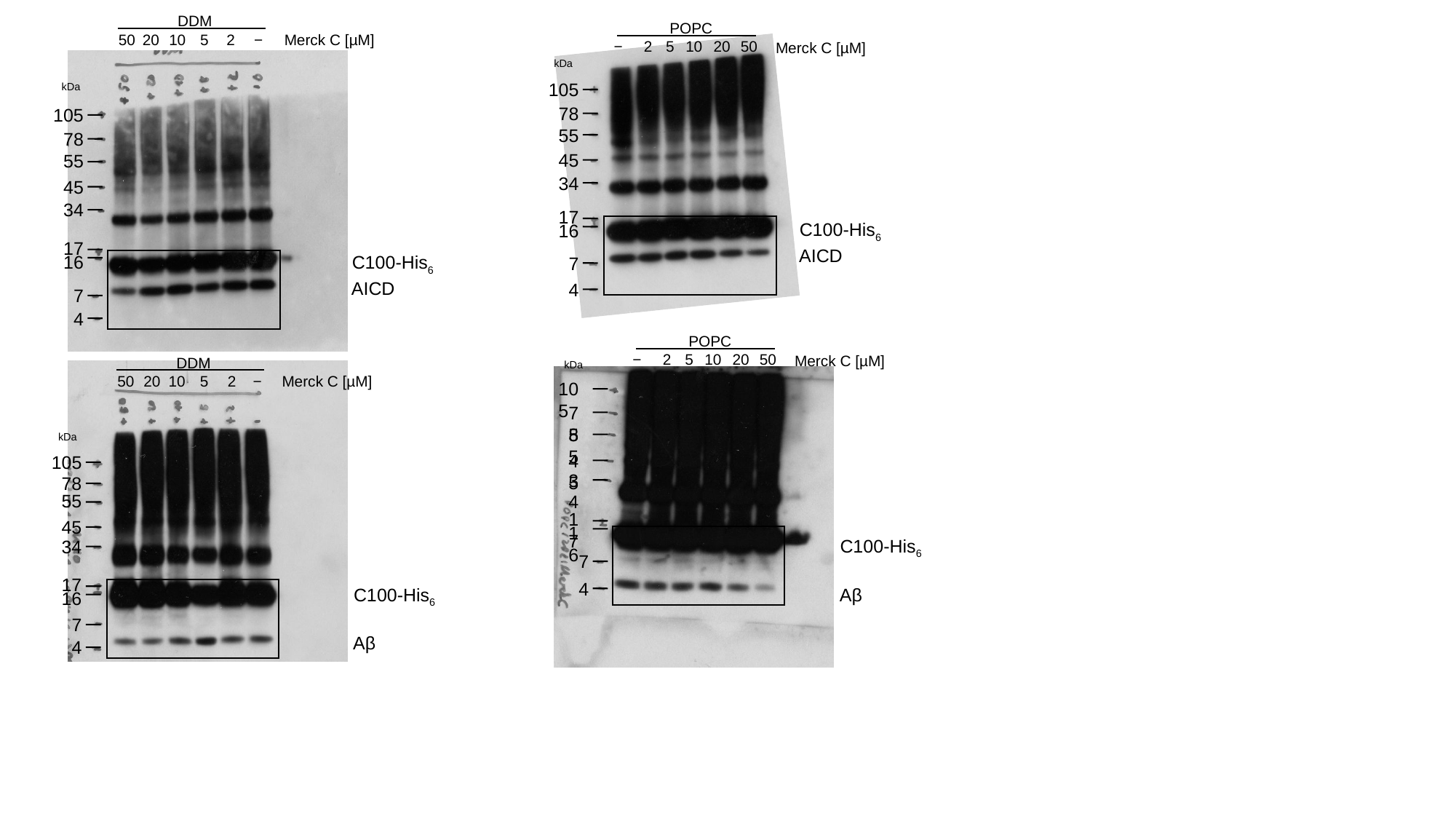

DDM
POPC
50
20
10
5
2
−
Merck C [µM]
−
2
5
10
20
50
Merck C [µM]
kDa
kDa
105
78
105
55
78
45
55
34
45
34
17
C100-His6
16
17
AICD
16
C100-His6
7
AICD
4
7
4
POPC
−
2
5
10
20
50
Merck C [µM]
kDa
DDM
50
20
10
5
2
−
Merck C [µM]
105
78
55
kDa
45
105
34
78
55
17
45
16
C100-His6
34
7
17
4
C100-His6
Aβ
16
7
Aβ
4

## Slide 2
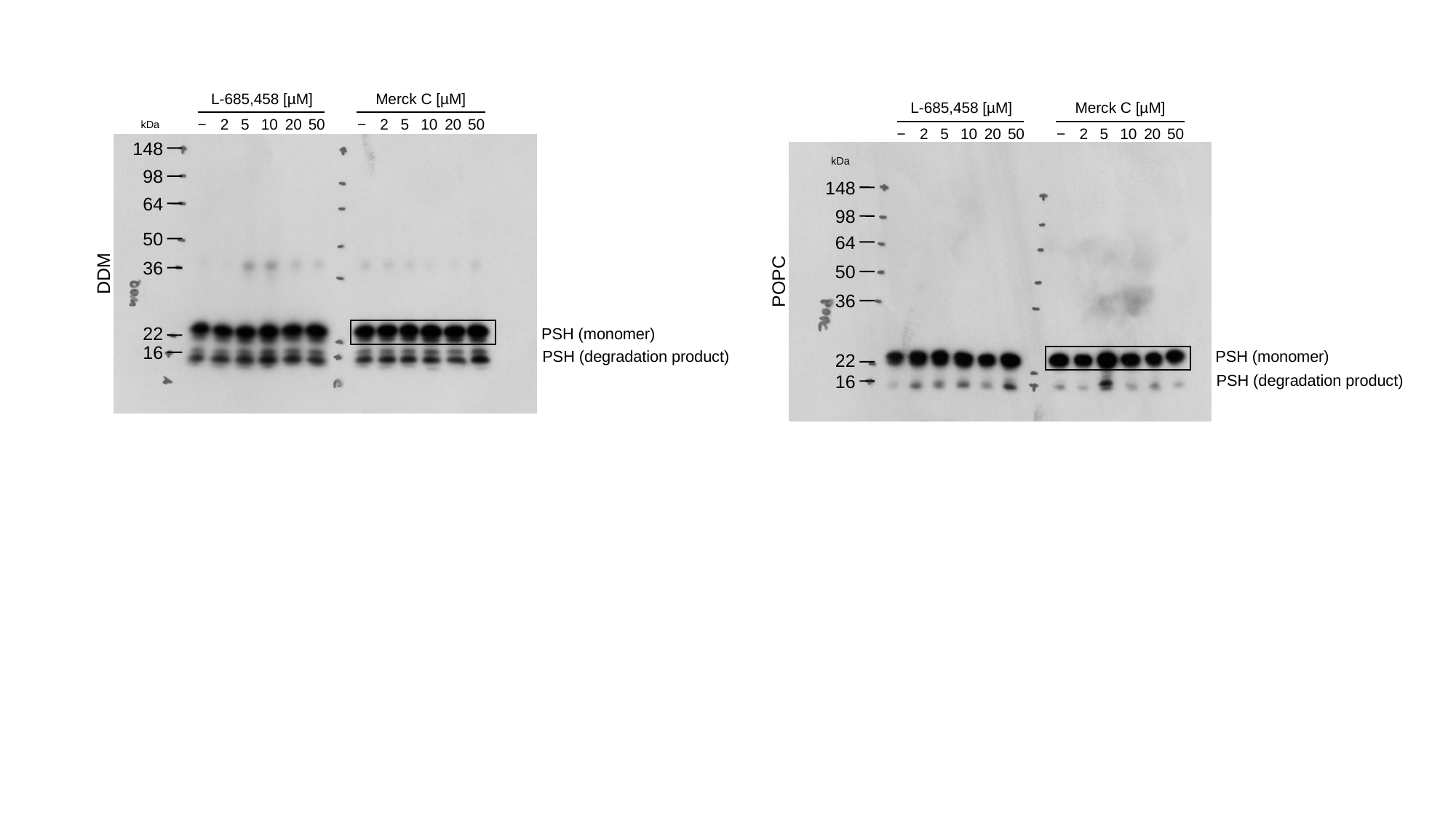

L-685,458 [µM]
Merck C [µM]
L-685,458 [µM]
Merck C [µM]
−
2
5
10
20
50
−
2
5
10
20
50
148
98
64
50
POPC
36
22
16
−
2
5
10
20
50
−
2
5
10
20
50
kDa
148
kDa
98
64
50
36
DDM
22
PSH (monomer)
16
PSH (monomer)
PSH (degradation product)
PSH (degradation product)
